# A Snf1-related nutrient-responsive kinase antagonizes endocytosis in yeast

**DOI:** 10.1371/journal.pgen.1008677

**Published:** 2020-03-19

**Authors:** Jessica M. Tumolo, Nathaniel L. Hepowit, Samika S. Joshi, Jason A. MacGurn

**Affiliations:** Department of Cell and Developmental Biology, Vanderbilt University, Nashville, Tennessee, United States of America; The University of North Carolina at Chapel Hill, UNITED STATES

## Abstract

Endocytosis is regulated in response to changing environmental conditions to adjust plasma membrane (PM) protein composition for optimal cell growth. Protein networks involved in cargo capture and sorting, membrane sculpting and deformation, and vesicle scission have been well-characterized, but less is known about the networks that sense extracellular cues and relay signals to trigger endocytosis of specific cargo. Hal4 and Hal5 are yeast Snf1-related kinases that were previously reported to regulate nutrient transporter stability by an unknown mechanism. Here we demonstrate that loss of Hal4 and Hal5 activates endocytosis of many different kinds of PM proteins, including Art1-mediated and Art1-independent endocytic events. Acute inhibition of Hal5 in the absence of Hal4 triggers rapid endocytosis, suggesting that Hal kinases function in a nutrient-sensing relay upstream of the endocytic response. Interestingly, Hal5 localizes to the PM, but shifts away from the cell surface in response to stimulation with specific nutrients. We propose that Hal5 functions as a nutrient-responsive regulator of PM protein stability, antagonizing endocytosis and promoting stability of endocytic cargos at the PM in nutrient-limiting conditions.

## Author summary

Cellular homeostasis, a fundamental requirement for all living organisms, is maintained in part through evolutionarily conserved mechanisms that regulate the abundance and activity of ion and nutrient transporters at the cell surface. These mechanisms often incorporate signaling networks that sense changes in the environment and relay signals to alter protein composition at the plasma membrane, often by inducing endocytosis of specific transporters in order to adjust and optimize transport activities at the cell surface. Here, we investigate two kinases in yeast–Hal4 and Hal5 –that are related to the yeast and human AMP sensing kinases. Loss of both Hal4 and Hal5 was previously reported to result in destabilization of ion and nutrient transporters by an unknown mechanism. Our data indicates that Hal kinases function broadly in the regulation of many different classes of endocytic cargo. Hal5 localizes to the plasma membrane in a manner that is responsive to nutrient availability and acute loss of Hal5 activity triggers rapid internalization of endocytic cargo. By uncovering a role for Hal5 as a nutrient-responsive regulator of endocytosis, this research sheds light on how signaling molecules regulate membrane trafficking events to coordinate adaptive growth responses.

## Introduction

Complex signaling networks sense and integrate information about the extracellular environment to coordinate diverse biological processes as part of the adaptive growth response. This involves multiple classes of integral membrane proteins, including signaling receptors, nutrient transporters, and ion channels, which all mediate complex transactions between a cell and its environment. On a systems level, coordinated action of the plasma membrane (PM) proteome dictates a cell’s response to its environment as well as interactions with neighboring cells in multicellular organisms, which are critical for regulation of cellular homeostasis in the face of changing environmental conditions.

The steady-state abundance of PM proteins is achieved through balance of multiple membrane trafficking pathways, including secretion, endocytosis, and endosomal recycling. A major way eukaryotic cells downregulate receptor, transporter, and ion channel activity is by endocytosis. Frequently, substrate (or ligand) engagement with cognate transporters (or receptors) selectively stimulates internalization by endocytosis and subsequent sorting on endosomes for delivery to the lysosome (or vacuole) for degradation. This type of endocytic downregulation has been described for many major facilitator family nutrient transporters in *Saccharomyces cerevisiae*, including Mup1 (a high-affinity methionine transporter) [1, 2], Can1 (a high-affinity arginine transporter) [3, 4], and Fur4 (a high-affinity uracil transporter) [5–8]. In each case, these proteins are stably expressed at the PM in the absence of their respective substrates, but are rapidly and selectively internalized and trafficked to the vacuole for degradation in the presence of specific substrates. Endocytic downregulation of these transporters is selective and ubiquitin-dependent [9–11]. Rsp5, the lone Nedd4 family E3 ubiquitin ligase in yeast, mediates ubiquitylation of endocytic cargo, often via interaction with an extensive network of arrestin-related adaptor proteins (ARTs) which target Rsp5 substrate selection in a context-dependent manner [9–11]. Cargo ubiquitylation is sufficient for capture by ubiquitin-binding elements in the endocytic machinery, and therefore understanding how extracellular cues are sensed and signals are relayed to trigger ubiquitylation of specific cargo at the PM will be critical for understanding the molecular basis for specificity in endocytic responses [12–14].

Snf1 is perhaps the best-characterized example of a nutrient-sensing regulator of endocytosis in *S*. *cerevisiae*. Snf1 and its human counterpart, AMPK, are evolutionarily conserved kinases that sense insufficient ATP levels through detection of increased intracellular concentrations of AMP and ADP [15]. Snf1 and AMPK regulate downstream effector pathways that coordinate catabolic processes to control energy homeostasis in the cell and thus are required for eukaryotic cells to adapt to various nutrient restrictive conditions [15]. In yeast, Snf1 functions as the catalytic subunit of a multi-protein kinase complex that undergoes regulation by subcellular localization and glucose availability [16, 17]. Part of Snf1 function involves regulation of endocytosis. Specifically, glucose availability regulates Snf1-mediated phosphorylation of the Rsp5 adaptor Art4 (Rod1), which contributes to the endocytic downregulation of the lactate transporter Jen1 [18] as well as hexose transporters Hxt1, Hxt3, and Hxt6 [19, 20]. Importantly, the activity of AMPK in human cells also regulates the stability and trafficking of the GLUT1 and GLUT4 glucose transporters by regulating the activity of TXNIP, an arrestin domain containing protein similar to the ART adaptors in yeast [21, 22]. These findings point to evolutionarily conserved cellular strategies for sensing nutrients and relaying signals to adjust the abundance of specific transporters at the PM.

Several kinases related to Snf1 [23, 24] are also reported to regulate membrane trafficking and nutrient metabolism in yeast. This family of kinases is characterized by sequence conservation in the catalytic domains and divergent N-terminal regions of uncharacterized function. One such kinase is Npr1, which has a catalytic domain comprising approximately half of the protein at the C-terminus, and an N-terminal domain that is heavily phosphorylated and regulated by the TORC1 kinase complex [25–29]. In a manner that is TORC1-sensitive, Npr1 can phosphorylate and inhibit the Rsp5 adaptor protein Art1, specifically by antagonizing Art1 localization to the PM and therefore stabilizing the arginine transporter, Can1, at the cell surface [27]. Similarly, endocytic downregulation of the yeast general amino acid transporter Gap1 is stimulated by TORC1 signaling through release of Npr1-mediated phosphoinhibition of the Rsp5 adaptors Bul1 and Bul2 [30, 31]. Thus, the Npr1 kinase provides an effector mechanism for TORC1 to regulate endocytosis of specific nutrient transporters at the PM. Ptk2, another kinase related to Snf1, is reported to regulate the activity of the essential yeast proton pump Pma1 in response to glucose availability and pH stress, although it remains unclear if this regulation occurs at the level of catalysis, or stability and trafficking [32–35]. These examples suggest that the broader family of Snf1-related kinases may generally function in the regulation of endocytosis, although many members of this family remain largely uncharacterized.

Hal4 and Hal5, two additional members of the Snf1-related family of kinases in yeast, are known to regulate nutrient uptake and metabolism [23] and have been best characterized for their redundant role in stabilizing two yeast potassium transporters, Trk1 and Trk2 [36–38]. In addition to defective ion and potassium homeostasis, *Δhal4Δhal5* (or *hal*) double mutant cells exhibit decreased steady state abundance of a variety of nutrient transporters including Mup1, Gap1, Can1, Fur4, Hxt1, and Tat2 [39, 40]. Not surprisingly, these mutants are broadly defective for nutrient uptake, and exist in a constitutively starved state due to lack of nutrients, as indicated by upregulation of the GCN transcriptional response pathway [40].

The phenotypes reported for *hal* double mutant cells suggest they play a role in stabilizing expression of nutrient transporters, although what process is specifically regulated by Hal kinases, the mechanism of action, and their relative contributions to cargo stabilization remain unknown. Here, we report that Hal kinases negatively regulate both Art1-mediated and Art1-independent trafficking of a broad spectrum of endocytic cargo, and that in some cases Hal4 and Hal5 exhibit distinct functions. We find that the previously uncharacterized N-terminal region of Hal5 is critical for regulation of cargo endocytosis as well as localization of Hal5 to the PM. Importantly, Hal5 localization to the PM is responsive to nutrient stimulation, as addition of specific nutrients triggered reduced Hal5 PM association. Taken together, our results indicate that Hal5 is a nutrient-responsive kinase that antagonizes endocytosis of several different classes of endocytic cargo, ultimately promoting their stability at the PM.

## Results

### Hal4 and Hal5 are yeast Snf1-related kinases

**Fig 1:**
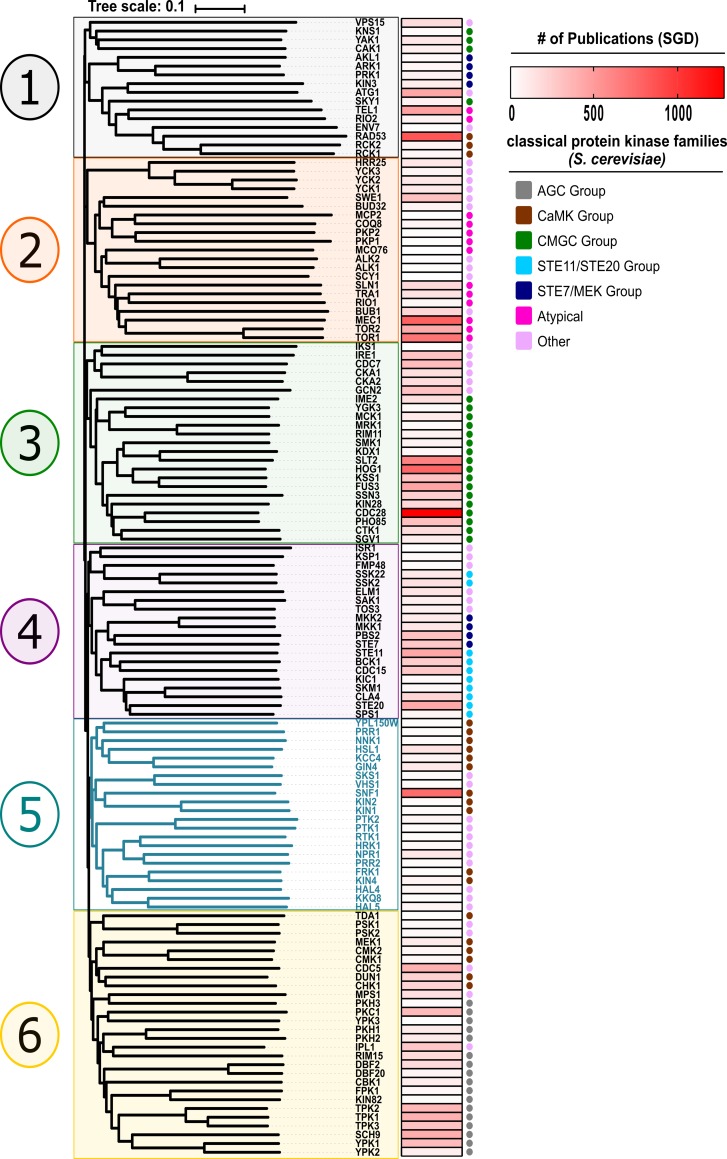
Hal4 and Hal5 cluster in a family of kinases that includes Snf1. A multiple sequence alignment of all 130 known protein kinases in yeast was performed using Clustal Omega and visualized as a scaled, rooted phylogenetic tree using iTOL. The protein kinases cluster into 6 major clades, which have been arbitrarily numbered and color-coded for simplicity and ease of viewing across different figures. A heat-map to the right of the phylogenetic tree conveys the number of publications annotated in SGD per kinase. Dots to the right of the heat map indicate the classical family assignment for each kinase.

**Fig 2:**
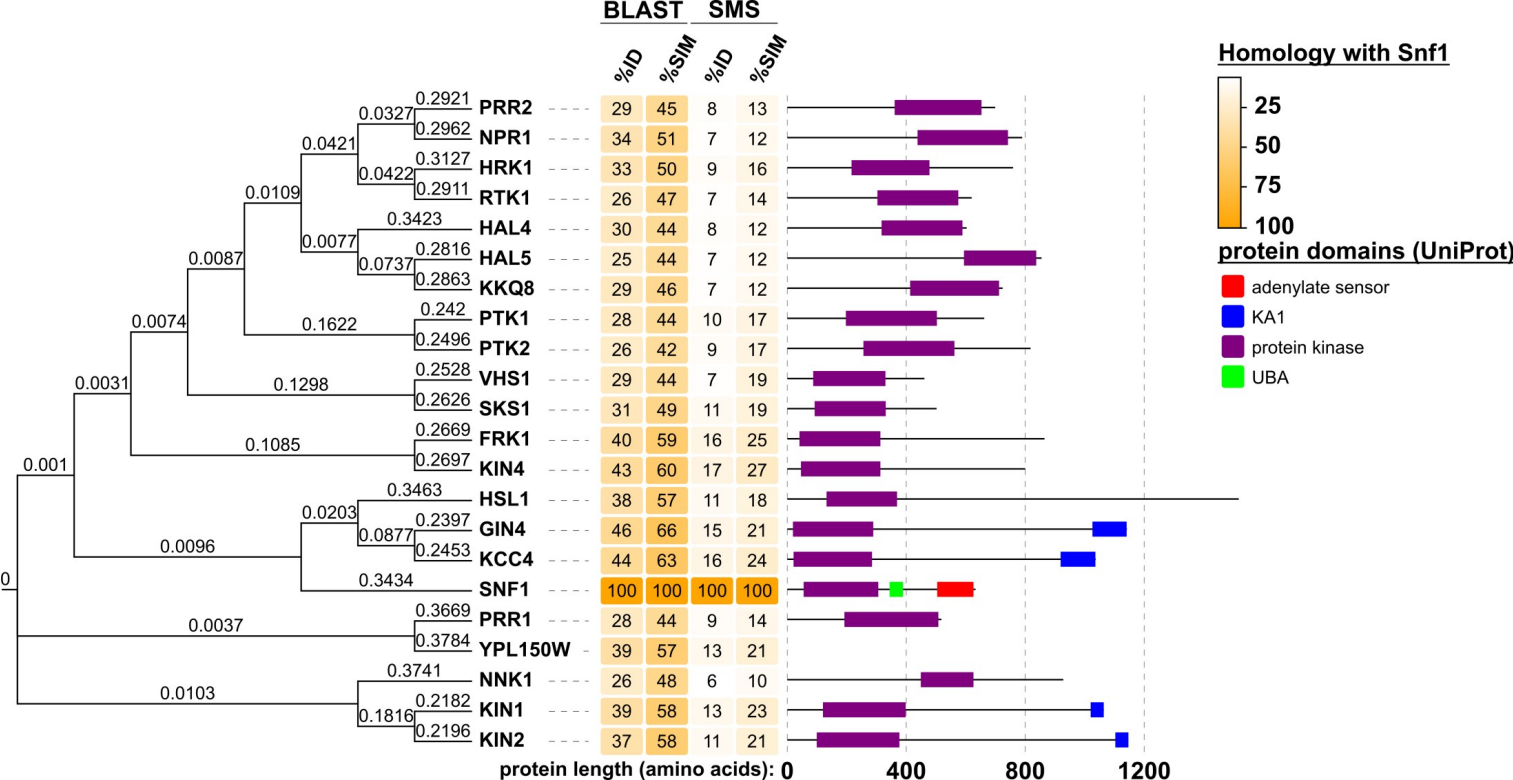
Kinase clustering with Snf1 is driven primarily by catalytic domain similarity. A multiple sequence alignment of the kinases that clustered with Snf1 in yeast was performed using Clustal Omega and visualized as a scaled, rooted phylogenetic tree using EvolView. To the right of the phylogenetic tree, sequence homology for each kinase with Snf1 is displayed as a 4-column heat-map divided into two sections. In the first section labeled BLAST, percent identity (%ID) or percent similarity (%SIM) was calculated by aligning the two sequences using NCBI-BLAST. In the second section labeled SMS percent identity (%ID) or percent similarity (%SIM) was calculated by aligning the two sequences using Sequence Manipulation Suite [107]. Both programs were used due to having different methods for calculating homology. BLAST calculates homology based on only the aligned region, which in every case is restricted to mostly the catalytic domains. While SMS also aligns sequences, its calculation accounts for the entirety of the protein sequences, demonstrating lower sequence homology outside of the catalytic domains. To the right of the sequence homology heat-map are protein architecture maps for each kinase drawn to scale. These were automatically generated in EvolView from data available for each kinase in UniProt. YPL150W is not annotated in UniProt, and therefore does not have a protein architecture map.

To better understand how Hal4 and Hal5 relate to other kinases in the yeast kinome, we performed a multiple sequence alignment of all 130 known protein kinases in yeast. Compared to previous analysis [23], our analysis differed in several ways, including ***(i)*** use of full-length primary sequence (as opposed to only catalytic domains), ***(ii)*** exclusion of known metabolic and lipid kinases, and ***(iii)*** use of the Clustal Omega sequence alignment tool [41]. Consistent with previous studies, our analysis (rooted in **Fig 1** and unrooted in **S1 Fig**) revealed six major kinase clades, with Hal4 and Hal5 clustering in a clade containing the yeast AMPK homolog Snf1. Snf1 is the best-studied member of this family while other members of this family are comparatively under-studied (based on number of publications, **Fig 1**). Of the 22 yeast kinases that cluster in this family, 11 were previously classified as belonging to the CaMK group and 11 (including Hal4 and Hal5) were classified as “Other” [23] (**Fig 1**, clade 5 (colored in blue)). We performed multiple sequence alignment of full-length primary sequence for each kinase in this family, and we observed a high degree of sequence conservation within the kinase domain but little homology outside the kinase domain (**Fig 2**). Interestingly, several kinases in this clade have accessory domains, such as the lipid-binding KA1 domain which has previously been shown to be important for localization to membranes [42] (**Fig 2**).

The Snf1 kinase in yeast is an ortholog of mammalian AMPK, and mammalian kinases related to AMPK have been sub-classified into the AMPK-related kinases (ARKs) and the Snf1-related kinases (SRKs), based on multiple criteria (see Discussion for a detailed explanation of these criteria). To better understand the clade of yeast kinases containing Snf1, we used SGD YeastMine (data populated by SGD, or *Saccharomyces* Genome Database, and powered by InterMine) to predict orthologs for each kinase across evolution (**S1 Table**) and found that 82% of orthologs predicted in other species correspond to AMPK, ARKs, or SRKs (**S2 Fig**). Although these ortholog predictions are not definitive, they suggest that the yeast family of kinases that cluster with Snf1 are related to the human family of kinases that cluster with AMPK. Interestingly, a multiple sequence alignment of activation loops for these kinases revealed that nine members of the yeast family (including Snf1) contain a conserved threonine in the activation loop, while the remaining members of this family lack this conserved threonine (**S3 Fig**). Hal4 and Hal5 kinases fall into the latter category, suggesting they are related to other kinases lacking a conserved activation loop threonine like Npr1, which has an established role in the regulation of endocytic trafficking [27, 31]. This association—along with previous reports that Hal4 and Hal5 are important regulators of nutrient transporter stability–led us to hypothesize that Hal4 and Hal5 may have functions similar to Npr1, prompting us to investigate whether or not Hal4 and Hal5 kinases are *bona fide* regulators of endocytic trafficking.

### Hal kinases regulate Art1-mediated and Art1-independent endocytosis

**Fig 3:**
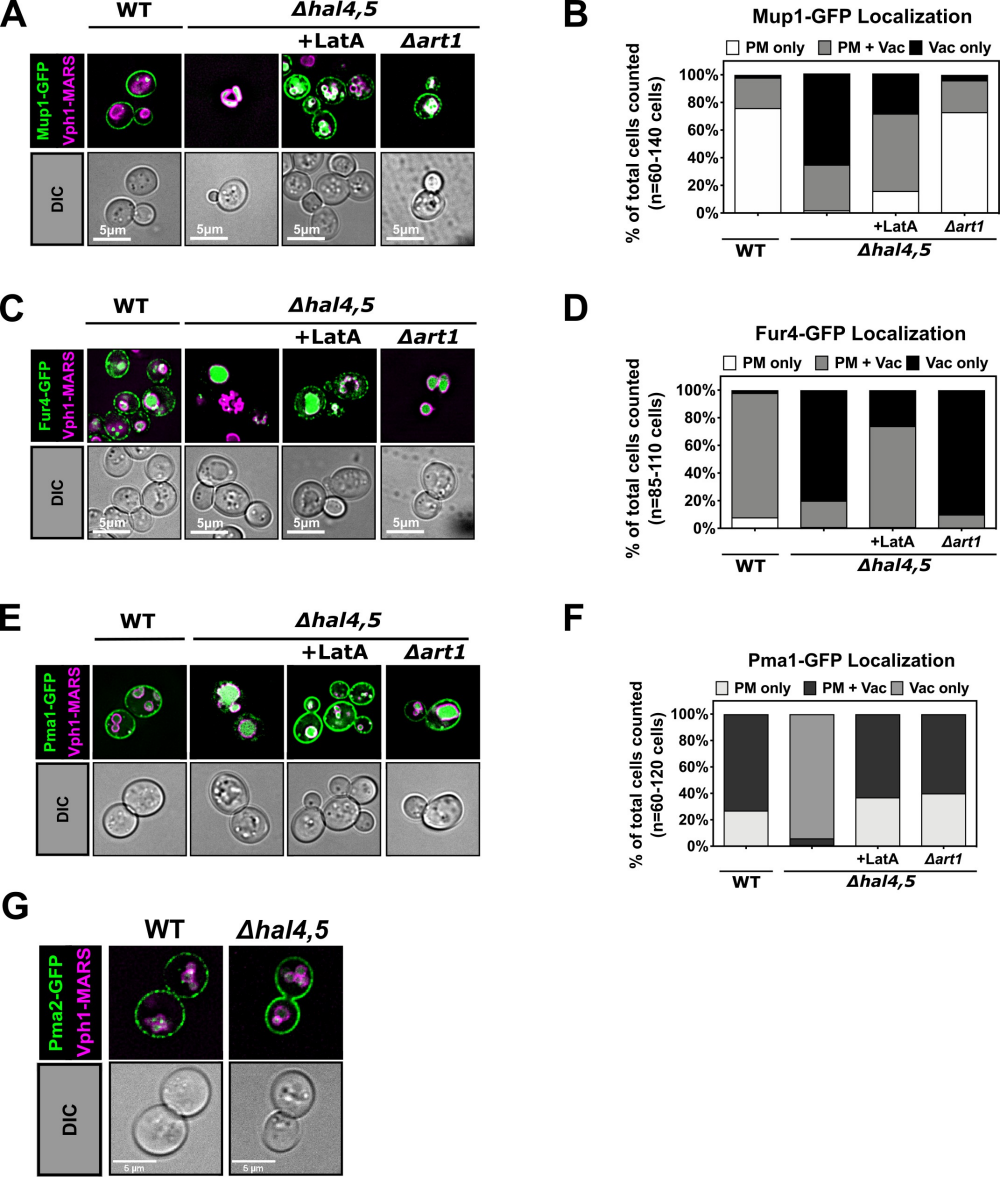
Hal kinases contribute to regulation of Art1-mediated and Art1-independent endocytosis. **(A)** Representative images of Mup1-GFP expressed from a centromeric plasmid under native promoter control in the presence of endogenously MARS tagged Vph1, a marker for the limiting membrane of the vacuole. WT, *Δhal4Δhal5* cells, or *Δhal4Δhal5Δart1* cells were cultured to mid-log phase in selective media and treated with LatA for 1 hour where indicated. **(B)** Quantification of Mup1-GFP localization in (A) performed by binning cells into localization categories as indicated. **(C)** Representative images of Fur4-GFP under conditions previously described in (A). **(D)** Quantification of Fur4-GFP localization in (C) performed as described in (B). **(E)** Representative images of Pma1-GFP under conditions previously described in (A). **(F)** Quantification of Pma1-GFP localization in (E) performed as described in (B). **(G)** Representative images of Pma2-GFP under conditions previously described in (A).

In *hal* double mutant cells many major facilitator family nutrient transporters—including Mup1, Gap1, Can1, Fur4, Hxt1, and Tat2—are mis-localized to the vacuole [39, 40]. Consistent with prior reports, we observed aberrant vacuolar localization for several of these transporters–including Mup1 (**Fig 3A and 3B**), Can1 (**S4 Fig**), and Fur4 (**Fig 3C and 3D**). To better define the precise trafficking step regulated by Hal kinases, we tested if the aberrant vacuolar trafficking observed in *hal* mutant cells requires endocytosis. Methionine-induced endocytic downregulation of Mup1 requires Art1, an adaptor protein for the E3 ubiquitin ligase Rsp5 [1]. Importantly, we found that loss of Art1 stabilized Mup1 at the PM in the absence of Hal kinases (**Fig 3A and 3B**), indicating that Art1-mediated endocytosis is required for vacuolar trafficking in *hal* mutant cells. In contrast, we found that loss of Art1 did not restore Fur4-GFP or Can1-GFP to the PM (**Fig 3C and 3D** and **S4 Fig**). However, treatment with Latrunculin A (LatA), an actin polymerization inhibitor known to block endocytosis [43–45], restored PM stability of both Fur4-GFP and Mup1-GFP in *hal* mutant cells (**Fig 3A–3D**). Taken together, these results indicate that different major facilitator family transporters undergo aberrant trafficking to the vacuole in *hal* mutant cells via endocytosis that can be Art1-mediated or Art1-independent.

To test if the scope of Hal kinase regulation is limited to major facilitator family transporters, we expanded our analysis to include other types of integral PM proteins in yeast. We observed that Smf1 –an Nramp family divalent metal ion transporter that traffics to the vacuole independent of Art1 [46]–exhibits aberrant vacuolar localization that is Art1-independent in *hal* mutant cells (**S4 Fig**). We also found that Pma1 –a P2-type proton pump and one of the most abundant proteins in the yeast plasma membrane–localized primarily to the vacuole in *hal* mutant cells and that this mis-localization could be suppressed either by addition of LatA or by deletion of *ART1* (**Fig 3E and 3F**). Previous studies reported aberrant vacuolar trafficking of Pma1 in v-ATPase mutants which was independent of Art1 but dependent on Art9 (Rim8) [47]. Importantly, loss of Hal kinases did not result in aberrant vacuolar trafficking of Pma2 (**Fig 3G**)–another P2-type proton pump and a paralog of Pma1 (86% identical). As with Pma2, we found that loss of Hal kinases did not affect the localization of the peripheral plasma membrane protein Pil1—a BAR domain protein and a core structural component of eisosomes [48, 49]. This analysis revealed no defects in Pil1-GFP localization or morphology in *hal* mutant cells (**S4 Fig**). Taken together with previous reports that *hal* mutant cells do not exhibit defects in localization of Sur7 (another component of eisosomes in yeast) [39], we conclude that some PM-associated proteins, like structural components of eisosomes, are not impacted by loss of Hal kinases.

**Fig 4:**
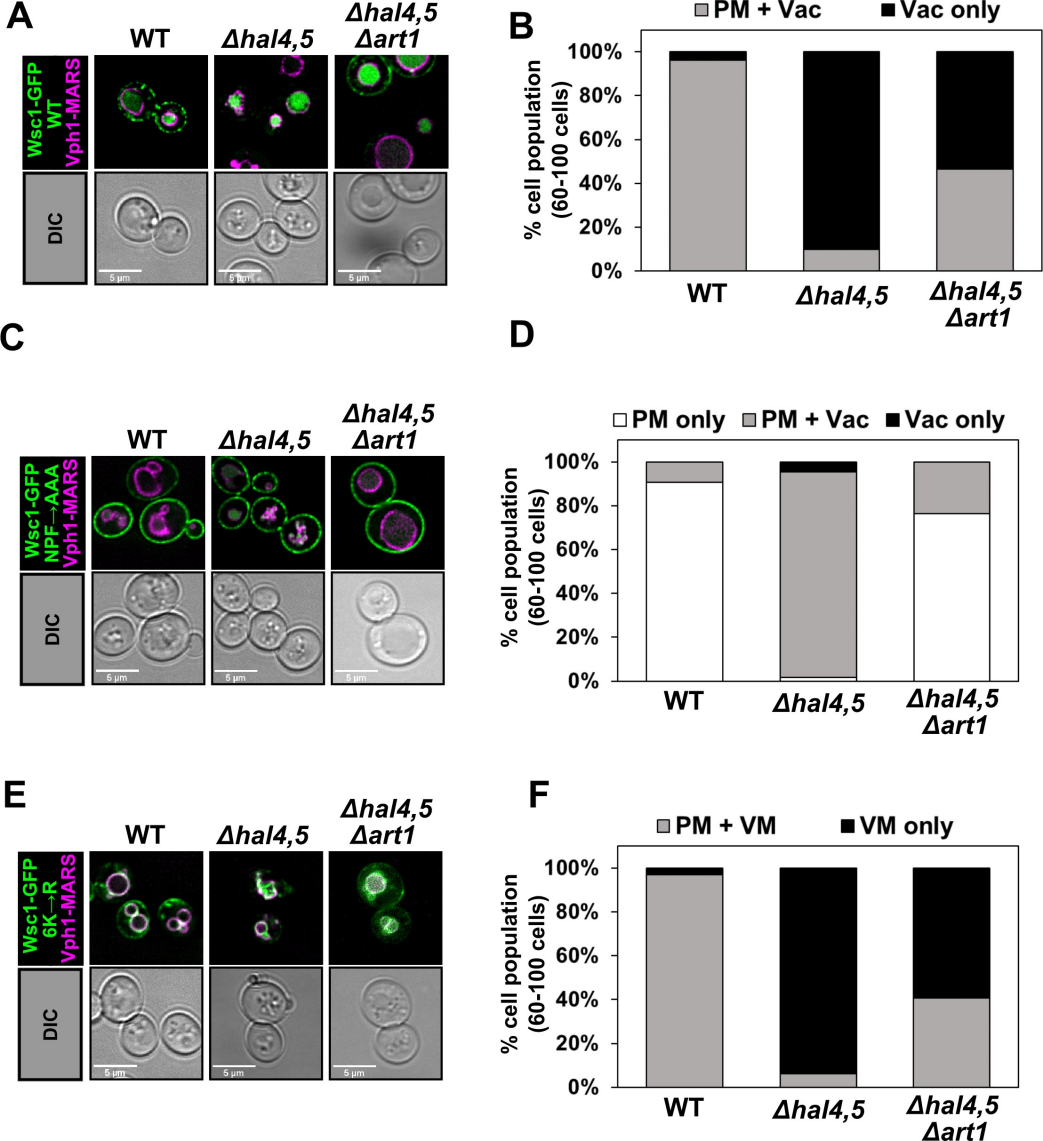
Hal kinases regulate the PM stability of the cell wall integrity sensor Wsc1. **(A)** Representative images of Wsc1-GFP expressed from a centromeric plasmid under native promoter control in the presence of endogenously MARS tagged Vph1, a marker for the limiting membrane of the vacuole. WT, *Δhal4Δhal5* cells, or *Δhal4Δhal5Δart1* cells were cultured to mid-log phase in selective media. **(B)** Quantification of Wsc1-GFP localization in (A) performed by binning cells into localization categories as indicated. **(C)** Representative images of a GFP-tagged Wsc1^NPF→AAA^ variant under conditions previously described in (A). **(D)** Quantification of Wsc1^NPF→AAA^ localization in (C) performed as described in (B). **(E)** Representative images of a GFP-tagged Wsc1^6K→R^ variant under conditions previously described in (A). **(F)** Quantification of Wsc1^6K→R^ localization in (E) performed as described in (B). VM indicates vacuole membrane localization.

We next examined the trafficking of Wsc1—a single transmembrane pass signal transducer in the cell wall integrity pathway known to undergo ubiquitin-independent endocytosis [50, 51]. Importantly, endocytosis of Wsc1 is mediated by direct interaction between its NPFxD motif and the SHD domain of Sla1 [50]. Furthermore, ubiquitination of Wsc1 appears to be dispensable for its endocytosis, although it is required for post-endocytic sorting on the endosome [51]. We observed aberrant vacuolar trafficking of Wsc1 in the absence of Hal kinases, which was partially Art1-dependent (**Fig 4A and 4B**). Since Wsc1 endocytosis is ubiquitin-independent and involves direct engagement with Sla1 [50, 51], we analyzed the trafficking of a Wsc1 mutant defective for Sla1 binding (Wsc1^NPF→AAA^ [50]). Unexpectedly, we found that Wsc1^NPF→AAA^ was almost exclusively PM localized in wildtype yeast cells but exhibited increased vacuolar trafficking in *hal* mutant cells (**Fig 4C and 4D**), although not to the extent of wildtype Wsc1 (**Fig 4A and 4B**). Importantly, the aberrant vacuolar trafficking of Wsc1^NPF→AAA^ in *hal* mutant cells was mostly Art1-dependent (**Fig 4C and 4D**). Taken together, these results indicate that the aberrant vacuolar trafficking of Wsc1 in *hal* mutant cells occurs by endocytosis involving both direct Sla1 interaction and a bypass endocytic mechanism that requires Art1. Aberrant vacuolar trafficking was also observed for a mutant variant of Wsc1 (*wsc1*^*6K→R*^) that lacks lysine residues critical for post-endocytic sorting [51] (**Fig 4E and 4F**), indicating that loss of Hal kinase activity promotes endocytosis but does not promote bypass of a post-endocytic sorting requirement for ubiquitylation of Wsc1 on the endosome.

Although our data are consistent with Hal kinases regulating endocytosis, we considered the possibility that *hal* mutant phenotypes may arise from defects in endosomal recycling. To test this, we analyzed the localization of Snc1-GFP, a v-SNARE which normally cycles between the PM and endosomes but exhibits aberrant endosomal localization in mutants defective for endosomal recycling [52–54]. Importantly, we found that Snc1-GFP is not mis-localized upon loss of Hal kinases (**S4 Fig**), suggesting that Hal kinases do not regulate endocytic recycling. To explore this further, we assayed endosome-to-PM lipid recycling by measuring efflux of a lipophilic tracer dye (FM 4–64) into the media [55–57]. We detected no difference in lipid recycling upon loss of Hal kinases compared to WT cells, in contrast to *Δrcy1* mutant cells which are known to exhibit lipid recycling defects (**S4 Fig**) [53, 56]. Additionally, we found that GFP-tagged carboxypeptidase S (CPS-GFP) [58, 59] exhibited normal vacuolar localization in *hal* mutant cells (**S4 Fig**), indicating that Hal kinases are not required for the transport of proteases to the lumen of the vacuole. Thus, *hal* mutant cells exhibit aberrant vacuolar trafficking of multiple PM proteins while endosomal recycling and localization of resident vacuole lumen proteins are unaffected.

Taken together, our analysis combined with previous analysis of cargo trafficking in *hal* mutant cells [39, 40] indicates that Hal kinases regulate the PM stability of a diverse assortment of cargo, although not all PM proteins tested exhibited aberrant localization (**Table 1**). Aberrant vacuolar trafficking in *hal* mutant cells is dependent on endocytosis, with no detectable effect on endosomal recycling. Loss of Hal kinases triggered Art1-mediated trafficking in the case of some cargo, while other cargo trafficked to the vacuole in a manner that is Art1-independent and may require the function of other arrestin-like proteins (**Table 1**). Furthermore, while deletion of *ART1* suppressed the aberrant trafficking of some cargo (**Table 1**) it did not suppress the slow growth phenotype observed for *hal* double mutant cells (**S4 Fig**) indicating that Art1-mediated activities like the destabilization of Pma1 (**Fig 3E and 3F**) do not fully account for the broad growth and endocytic trafficking phenotypes observed in *hal* mutant cells.

**Table 1:** Summary of cargo trafficking phenotypes in *hal* double mutant cells.

**No aberrant mis-localization observed in *hal* mutant cells**	
Pma2	P2-type proton pump, 86% identical to Pma1
Pil1	BAR domain protein, component of eisosomes
Snc1	VAMP family R-SNARE, mediates fusion of secretory vesicles with PM
**Aberrant vacuolar trafficking observed in *hal* mutant cells (Art1-mediated)**	
Mup1	Major facilitator family, methionine transporter
Pma1	P2-type proton pump
Wsc1	Cell wall integrity sensor
**Aberrant vacuolar trafficking observed in *hal* mutant cells (Art1-independent)**	
Fur4	Major facilitator family, uracil transporter
Can1	Major facilitator family, arginine transporter
Smf1	Nramp family divalent metal ion transporter

### Hal4 and Hal5 function redundantly in the stabilization of endocytic cargo

**Fig 5:**
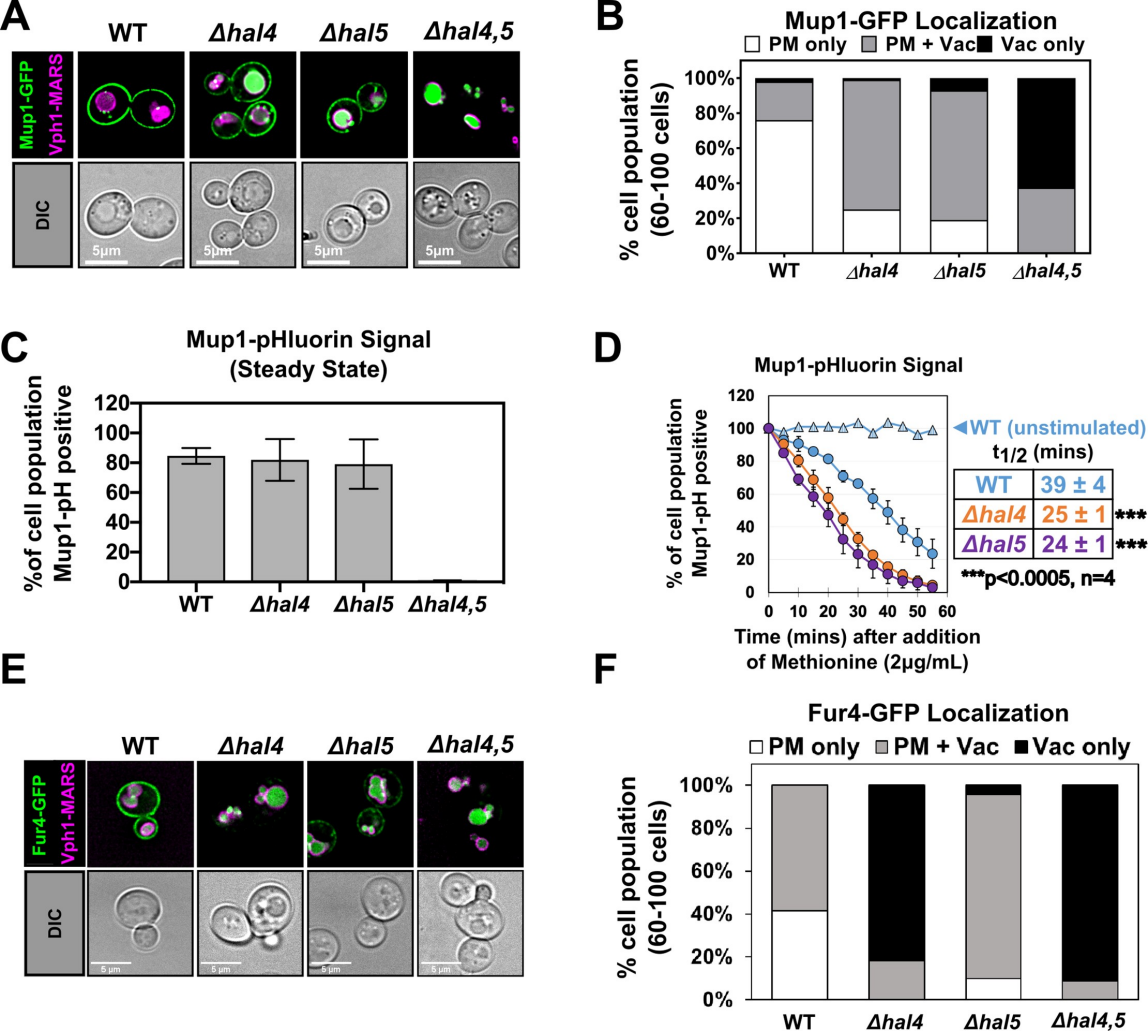
Hal4 and Hal5 exhibit redundant roles with respect to nutrient transporter localization. **(A)** Representative images of Mup1-GFP expressed from a centromeric plasmid under native promoter control in the presence of endogenously MARS tagged Vph1, a marker for the limiting membrane of the vacuole. WT, *Δhal4* or *Δhal5* single mutant cells or *hal* double mutant cells were cultured to mid-log phase in selective media. **(B)** Quantification of Mup1-GFP localization in (A) performed by counting a population of cells and binning each cell into a cargo localization category (PM only, PM + Vac, or Vac only). **(C)** Percentage of cell population expressing endogenously tagged Mup1-pHluorin as measured by cells that fall within a defined FITC gate (green fluorescence) by flow cytometry at steady state (10,000 cells counted per condition, n = 4 biological replicates). **(D)** Percentage of cell population expressing endogenously tagged Mup1-pHluorin as measured by cells that fall within a defined FITC gate (green fluorescence) by flow cytometry (10,000 cells counted per condition, n = 4 biological replicates) over time in the presence of excess methionine, an endocytic stimulant. Mup1-pH PM half-time (t_1/2_) was estimated based on initial and final time points and elapsed time. **(E)** Representative images of Fur4-GFP under conditions previously described in (A). **(F)** Quantification of Fur4-GFP localization in (E) performed as described in (B).

Although destabilization of nutrient transporters has been previously reported for *hal* double mutant cells, the relative contributions of Hal4 and Hal5 to these aberrant trafficking phenotypes have not been addressed. In contrast to *hal* double mutant cells, we found that *Δhal4* and *Δhal5* single mutant cells grew similarly to wildtype cells on solid or liquid media (**S5 Fig**), suggesting that Hal4 and Hal5 have redundant functions with respect to cell growth. Based on this observation, we hypothesized that Hal4 and Hal5 have redundant (or partially redundant) functions with respect to regulation of PM protein stability. Alternatively, the broad trafficking phenotypes observed might result from the sum of distinct cargo specificities. Our analysis of Mup1-GFP revealed that *Δhal4* and *Δhal5* single mutant cells exhibited increased vacuolar localization compared to wildtype cells (**Fig 5A and 5B**). However, unlike *hal* double mutant cells, *Δhal4* and *Δhal5* single mutant cells both exhibit significant Mup1 PM localization (**Fig 5A**), suggesting partially redundant functions with respect to Mup1 PM stability. To more specifically characterize Mup1 signal at the PM, we measured fluorescence of Mup1 tagged with pHluorin, a pH-sensitive GFP variant that quenches upon encountering acidic intracellular compartments [60]. Strikingly, we found that *hal* double mutant cells exhibited no Mup1-pHluorin signal at steady state (**Fig 5C**), consistent with fluorescence microscopy analysis that revealed vacuolar localization of Mup1 in these cells (**Fig 5A**). In contrast, *Δhal4* and *Δhal5* single mutant cells exhibited significant Mup1-pHluorin signal intensity at steady state (**Fig 5C**), consistent with fluorescence microscopy analysis revealing PM localization of Mup1-GFP in these cells (**Fig 5A**). Based on these measurements, we hypothesized that *Δhal4* and *Δhal5* single mutant cells may exhibit an increased rate of endocytic trafficking of Mup1. To test this, we measured the rate of internalization of Mup1-pHluorin from the plasma membrane in response to methionine [61] and found that Mup1 internalizes faster in *Δhal4* and *Δhal5* single mutant cells compared to wildtype cells (**Fig 5D**). These results indicate that both Hal4 and Hal5 contribute to the regulation of Mup1 endocytic trafficking, and loss of either kinase increases the rate of Mup1 delivery to the vacuole.

To further examine the relative contributions of Hal4 and Hal5 kinases to the regulation of endocytosis, we analyzed the subcellular localization of Fur4-GFP in *Δhal4* and *Δhal5* single mutant cells. In contrast to *Δhal5* single mutant cells, we found that *Δhal4* single mutant cells exhibited aberrant vacuolar trafficking of Fur4 similar to *hal* double mutant cells (**Fig 5E and 5F**), suggesting that Hal4 kinase activity is required (and at least partially sufficient) for Fur4 PM stability. Analysis of Can1-GFP localization revealed that *Δhal4* single mutant cells exhibited increased vacuolar localization compared to both wildtype and *Δhal5* single mutant cells, which were statistically indistinguishable (**S5 Fig**). Interestingly, analysis of sensitivity to canavanine (a toxic analog of arginine, which correlates with stability of Can1 at the PM [1]) revealed a striking canavanine resistance in *hal* double mutant cells, despite slight canavanine sensitivities observed for both *Δhal4* and *Δhal5* single mutant cells (**S5 Fig**). These results suggest redundant functions of the two kinases and complete clearance of Can1 from the PM in *hal* double mutant cells (as was observed in **S4 Fig**). Importantly, deletion of *ART1* did not suppress the canavanine resistance *hal* double mutant cells (**S5 Fig**), consistent with Art1-independent trafficking of Can1 to the vacuole in these cells (**S4 Fig**). Taken together, our results support redundancy of Hal4 and Hal5 functions in the regulation of Mup1 and Can1 PM stability, while aberrant vacuolar trafficking of Fur4 observed in *hal* double mutant cells can be attributed primarily to the loss of Hal4.

We also observed that *Δhal4* and *Δhal5* single mutant cells exhibited different sensitivities when exposed to a variety of stress conditions (**S5 Fig**). *Δhal5* single mutant cells were sensitive to tunicamycin, while *Δhal4* single mutant cells were sensitive to growth in low glucose conditions, suggesting distinct roles for Hal4 and Hal5 in different environmental conditions. Furthermore, *Δhal5* single mutant cells, but not *Δhal4* cells, exhibited sensitivity to metal ion (MnCl_2_) and salt (LiCl) stresses (**S5 Fig**). Both *Δhal4* and *Δhal5* single mutant cells exhibited growth comparable to WT cells in the presence of caffeine **(S5 Fig**). Overall, our phenotypic analysis reveals that Hal4 and Hal5 have distinct cellular functions, in addition to having some redundant functions with respect to stability of integral membrane proteins at the PM.

### Acute inhibition of Hal5 triggers rapid endocytic clearance of multiple cargo

To determine if Hal5 kinase activity is required for negative regulation of endocytosis, we first aligned the primary amino acid sequence and the predicted catalytic domain structure of Hal5 with Snf1 (**S6 Fig**). This analysis revealed residues predicted to be important for Hal5 kinase function, including the conserved ATP-coordinating lysine residue (K546) and a conserved aspartic acid residue critical for the catalytic mechanism (D688). We then generated and characterized a variant of Hal5 mutated at this conserved aspartic acid residue (*hal5-D688A*). While wildtype Hal5 (untagged and containing a C-terminal 6XHis-Tev-3XFLAG tag) complemented the aberrant trafficking of Mup1-GFP to the vacuole and the increased rate of endocytosis observed for Mup1-pHluorin, catalytic dead Hal5 failed to complement these phenotypes (**S7 Fig** and **S8 Fig**). However, immunoblot analysis revealed that the Hal5-D688A catalytic dead variant was not stably expressed (**S8C Fig**), suggesting that kinase activity is required for Hal5 stability. To explore this further, we analyzed a variant of Hal5 mutated at the conserved ATP-coordinating lysine residue (*hal5-K546R*) and found that this variant also exhibited loss of protein stability (**S8 Fig**). These findings indicate that Hal5 catalytic dead mutants are unstable, limiting our ability to draw conclusions about Hal5 mechanism of action using catalytic dead variants.

**Fig 6:**
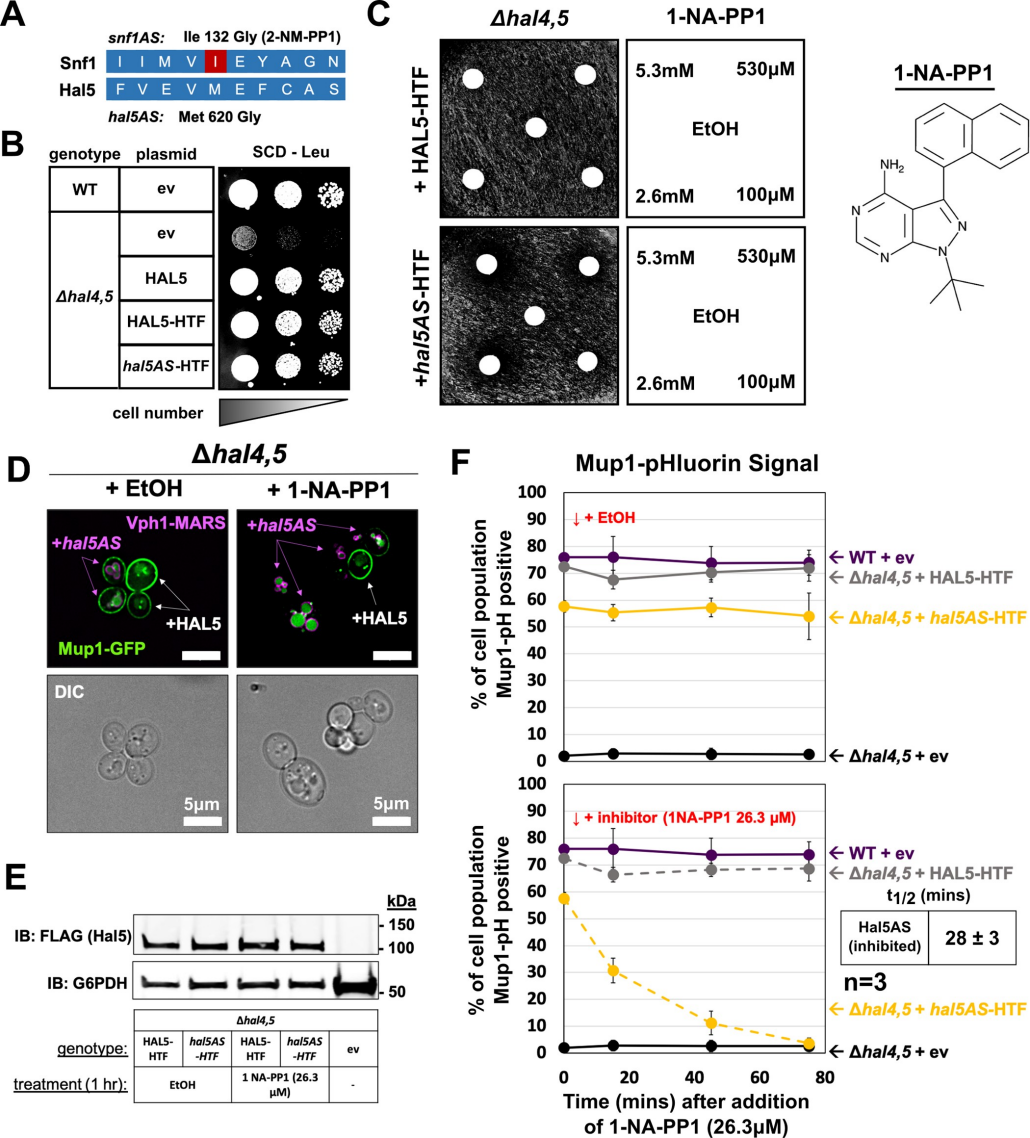
Hal5 catalytic activity antagonizes the endocytic trafficking of Mup1. **(A)** Schematic representation of Hal5 sequence alignment with Snf1 to identify Met620 as the gatekeeper residue. Snf1 is the most-related kinase for which an analog-sensitive allele has been previously characterized (*snf1AS*: I132G inhibited by 2-NM-PP1) **(B)** Cells were serially diluted onto synthetic selective media and grown for 3 days to assay functionality of *hal5AS* by growth. **(C)** Representative image of *Δhal4*,*5* mutant cells expressing either WT (HAL5-HTF) or analog-sensitive (*hal5AS-*HTF) Hal5 spread onto synthetic selective media as a lawn. Whatman paper disks soaked in a solution of 1-NA-PP1 dissolved in vehicle (EtOH) at the indicated concentrations were placed on top of the lawn prior to incubation to establish a concentration gradient of inhibitor. Plates were grown for 3 days to assess cell growth, and therefore inhibition of *hal5AS* by 1-NA-PP1 (structure shown in right of panel). **(D)** Representative images of Mup1-GFP localization in *Δhal4*,*5* mutant cells exogenously expressing either HAL5 (white arrow indicators) or *hal5AS* (magenta arrow indicators, cells marked by Vph1-MARS, a marker of the vacuolar limiting membrane). Cells were co-cultured in synthetic selective media to mid-log phase then treated with vehicle (EtOH) or inhibitor (1-NA-PP1 26.3μM) for 1 hr, then imaged. **(E)** Immunoblot analysis of *Δhal4*,*5* mutant cells exogenously expressing empty vector (ev), WT (HAL5-HTF), or analog-sensitive (*hal5AS-*HTF) Hal5, under indicated conditions to asses Hal5 protein expression. **(F)** Percentage of cell population expressing endogenously tagged Mup1-pHluorin as measured by cells that fall within a defined FITC gate (green fluorescence) by flow cytometry at steady state (10,000 cells counted per condition, n = 3 biological replicates) over time in the presence of *hal5AS* inhibitor 1-NA-PP1 or mock treatment (EtOH). WT or analog-sensitive HAL5 (HAL5-HTF or *hal5AS-*HTF) is exogenously expressed in *Δhal4*,*5* or *5* mutant cells from a centromeric plasmid under native promoter control. EV indicates empty vector.

**Fig 7:**
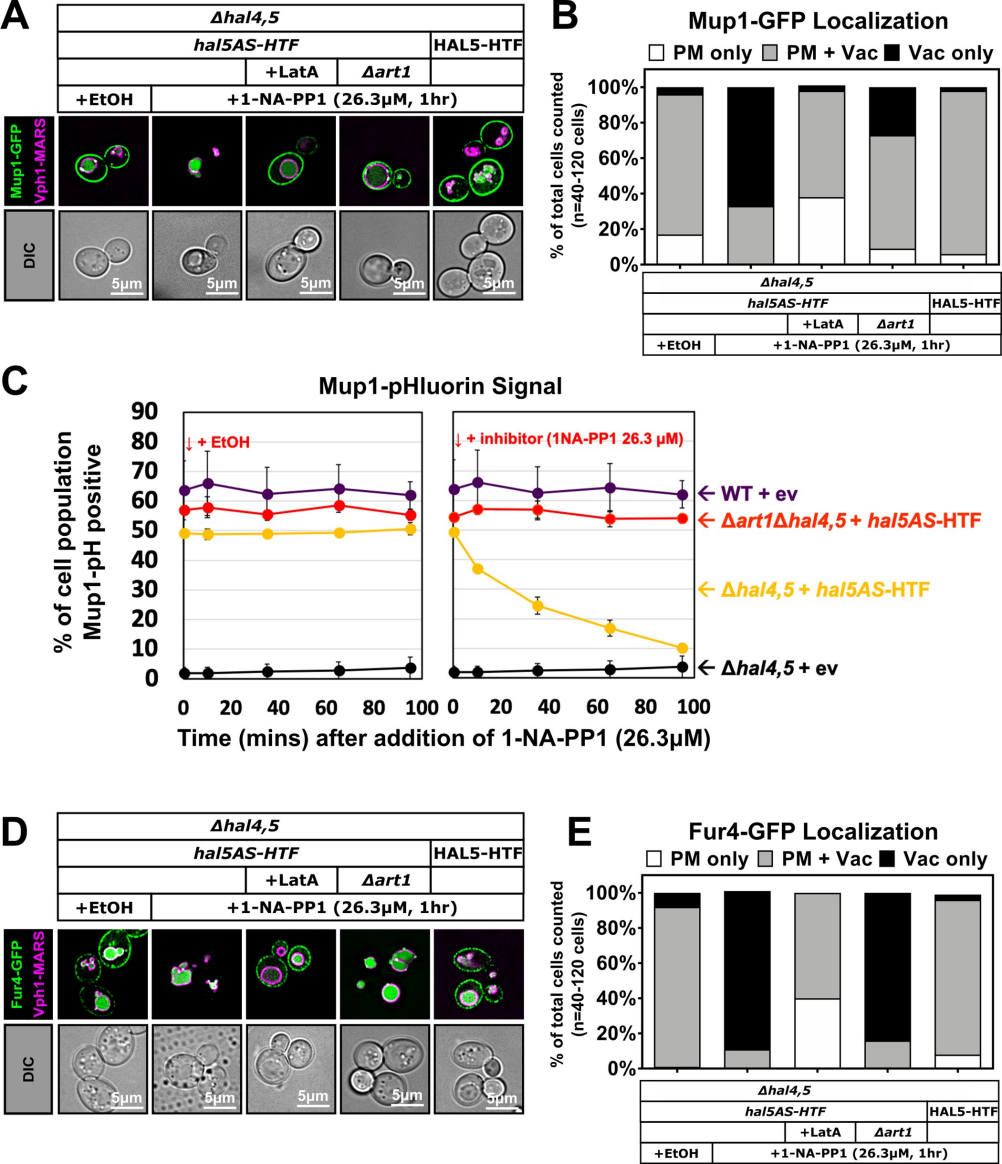
Acute inhibition of Hal5 activity triggers rapid endocytic clearance of nutrient transporters. **(A)** Representative images of Mup1-GFP expressed from a centromeric plasmid in the presence of endogenously-tagged Vph1-MARS, a marker for the limiting membrane of the vacuole. WT or analog-sensitive HAL5 (HAL5-HTF or *hal5AS-*HTF) is exogenously expressed in *Δhal4*,*5* or *Δhal4*,*5Δart1* mutant cells from a centromeric plasmid under native promoter control as indicated. Cells were grown to mid-log phase in selective media and imaged after 1 hr of inhibitor treatment (1-NA-PP1). Where indicated, cells were pre-treated with LatA for 1 hr prior to 1-NA-PP1 inhibition. **(B)** Quantification of Mup1-GFP localization in (A) performed by binning cells into localization categories as indicated. **(C)** Percentage of cell population expressing endogenously tagged Mup1-pHluorin as measured by cells that fall within a defined FITC gate (green fluorescence) by flow cytometry at steady state (10,000 cells counted per condition, n = 3 biological replicates) over time in the presence of *hal5AS* inhibitor 1-NA-PP1. WT or analog-sensitive HAL5 (HAL5-HTF or *hal5AS-*HTF) is exogenously expressed in *Δart1Δhal4*,*5* mutant cells from a centromeric plasmid under native promoter control. EV indicates empty vector. **(D)** Representative images of Fur4-GFP under conditions described previously in (A). **(E)** Quantification of Fur4-GFP localization in (D) performed as described in (B).

To better understand the role of Hal5 catalytic activity in regulation of endocytic trafficking, we adopted a chemical-genetic strategy [62] to develop an analog-sensitive allele of Hal5 (called *hal5AS*) by mutating the conserved gatekeeper residue (M620G) in the ATP binding pocket (based on the corresponding position of the kinase Snf1 [63]) (**S6 Fig** and **Fig 6A**). Importantly, the *hal5AS* allele was functional and complemented the growth defect in *hal* mutant cells (**Fig 6B**) while addition of a PP1 analog (1-NA-PP1) induced a growth defect (**Fig 6C**). Thus, the *hal5AS* variant exhibits functional kinase activity that can be inhibited with 1-NA-PP1. Using the *hal5AS* allele, we analyzed endocytic trafficking following acute inhibition of Hal5 kinase activity and observed that addition of 1-NA-PP1 induced Mup1-GFP trafficking to the vacuole in cells expressing *hal5AS* but not wildtype *HAL5* (**Fig 6D**). Importantly, acute inhibition of Hal5AS by 1-NA-PP1 did not induce protein instability (following a 60 minute treatment) as observed with catalytic dead variants of *Hal5* (**Fig 6E**), demonstrating that induction of vacuolar trafficking is not due to loss of Hal5 protein. To better characterize the kinetics of induced endocytosis following acute inhibition of *Hal5* (in a *Δhal4* background), we analyzed Mup1-pHluorin trafficking and found that 1-NA-PP1 triggered loss of Mup1-pHluorin signal with a half-time of ~28 minutes (**Fig 6F**), which is faster than methionine-induced endocytosis measured in wildtype cells (**Fig 5D**). As expected, Art1 was required for endocytosis of Mup1-pHluorin following acute inhibition of Hal5 (**Fig 7A–7C**). Importantly, acute inhibition of Hal5 (in a *Δhal4* background) also triggered rapid endocytosis and vacuolar trafficking of Fur4-GFP, a response which was inhibited by addition of LatA, but occurred independently of Art1 (**Fig 7D and 7E**).

Taken together, these data indicate that ***(i)*** *Hal5* kinase activity is required for its role in the regulation of endocytic trafficking, ***(ii)*** *Hal5* kinase activity stabilizes PM proteins and inhibition of Hal5 kinase activity (in the absence of Hal4) triggers rapid endocytosis and vacuolar trafficking, and ***(iii)*** induction of endocytosis and vacuolar trafficking is an acute and rapid response to loss of Hal kinase activity, rather than a chronic adaptive condition in *hal* double mutant cells.

### Loss of Hal kinase activity produces no observable effect on Art1

Our genetic evidence that loss of Hal kinases triggers Art1-mediated endocytic events suggests that Hal kinases may directly regulate Art1. Given previous reports that Art1 is regulated by Npr1 [27]—a kinase closely related to Hal4 and Hal5 –we hypothesized that Hal kinases antagonize endocytosis by inhibiting Art1 in a manner similar to Npr1. Unexpectedly, *hal* double mutant cells (but not *Δhal4* or *Δhal5* single mutant cells) exhibited a slight but significant decrease in Art1 abundance (**S9 Fig**). To test if Hal kinases regulate Art1 phosphorylation we performed SILAC-MS quantitative phosphoprofiling analysis of Art1 (**S10 Fig, S11 Fig, S12 Fig** and **S13 Fig**). First, we compared Art1 phosphorylation in wildtype cells to *hal* double mutant cells and detected only minor changes corresponding to slightly elevated phosphorylation at some N-terminal sites in the absence of Hal kinases (**S10 Fig**). Additionally, we compared Art1 phosphorylation in the context of acute Hal5AS inhibition and similarly detected only minor changes corresponding to slightly elevated phosphorylation at some sites in the absence of Hal kinase activity (**S10 Fig**). Indeed, two regulatory modifications recently reported to inhibit Art1 activity–phosphorylation of Thr93 and Thr795 [64]–were either unaffected or slightly elevated in the absence of Hal kinase activity (**S11 Fig, S12 Fig** and **S13 Fig**). Importantly, these experiments also reveal that loss of Hal kinase activity does not impact the interaction between Art1 and Rsp5 (**S10 Fig**). Since Art1 activation is known to involve its translocation to the PM [27], we considered the possibility that Hal kinases might regulate Art1 localization. To test this, we analyzed Art1 subcellular localization and found that, in contrast to *Δnpr1* mutant cells [27], *hal* mutant cells exhibited no observable increase in Art1 localization to the PM (**S14 Fig**). Thus, although we cannot exclude the possibility that Hal kinases antagonize endocytosis by inhibiting Art1 function, the data presented here do not provide evidence that Hal kinases regulate phosphorylation or localization of Art1.

In the course of phosphoprofiling Art1 following acute inhibition of Hal5AS we were also able to acquire similar phosphoprofiling data for Hal5 (since Hal5 was FLAG-tagged in these experiments and thus was captured as a bait). This analysis revealed two phosphorylation events at Ser358 and Ser395 that were reduced following acute inhibition of Hal5 kinase activity (**S15 Fig**), indicating these serine residues may be auto-phosphorylated. To our knowledge this data provides the first evidence of a substrate for Hal5 kinase activity in yeast cells and suggests that Hal5 may auto-regulate the function of an uncharacterized feature in its own N-terminus.

### N-terminal elements of Hal5 are required for regulation of cargo endocytosis

**Fig 8:**
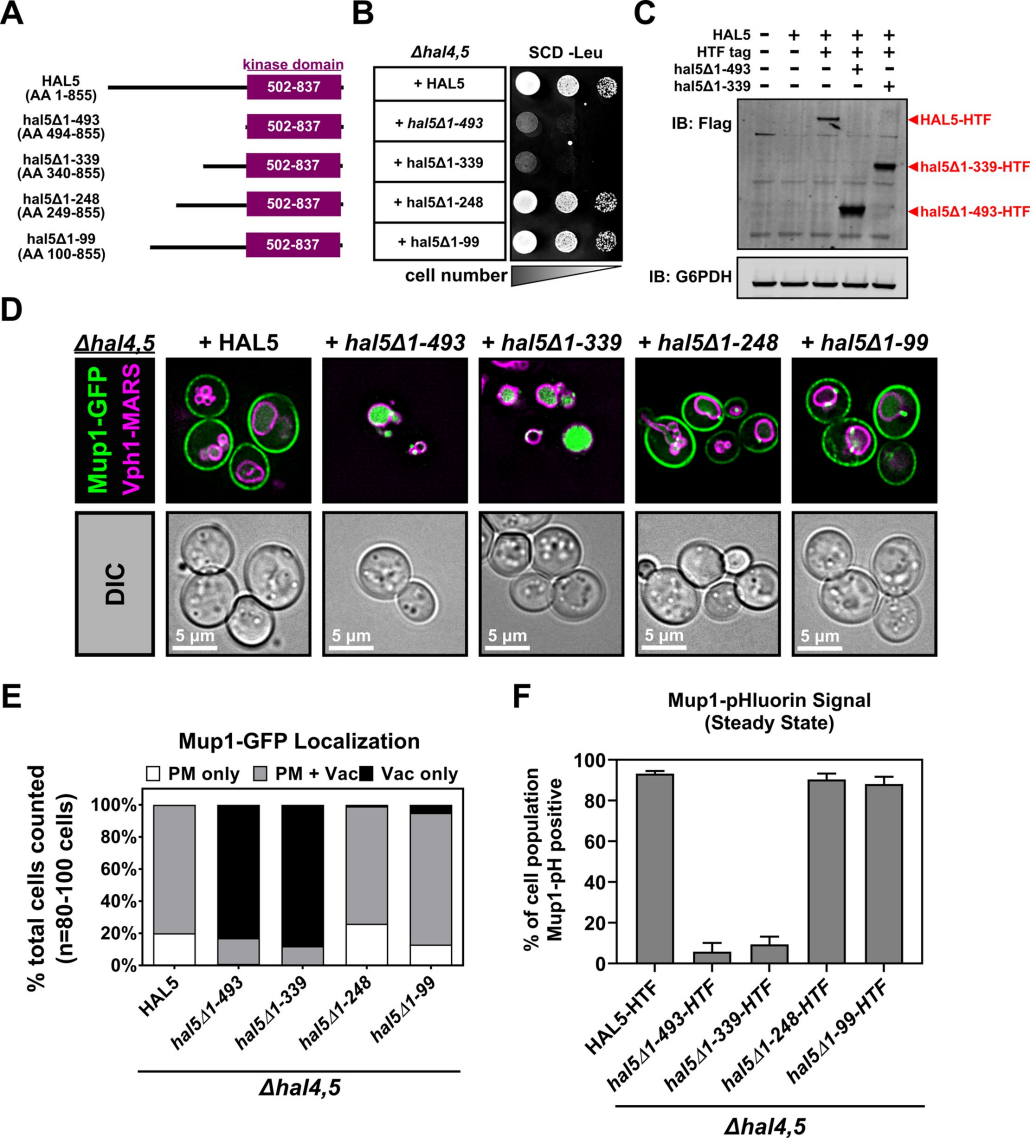
N-terminal elements of Hal5 are critical for antagonizing nutrient transporter endocytosis. **(A)** Schematic representation of Hal5 truncation variants compared to WT Hal5. **(B)** Representative image of cells serially diluted on synthetic selective media and grown for 3 days. **(C)** Immunoblot analysis to examine expression of Hal5 variants that fail to complement growth in *hal* mutant cells. **(D)** Representative images of Mup1-GFP expressed from a centromeric plasmid in the presence of endogenously-tagged Vph1-MARS, a marker for the limiting membrane of the vacuole. Hal5 variants are exogenously expressed in *Δhal4*,*5* mutant cells from a centromeric plasmid under native promoter control. **(E)** Quantification of Mup1-localization in (D) performed by binning cells into localization categories as indicated. **(F)** Percentage of cell population expressing endogenously tagged Mup1-pHluorin as measured by cells that fall within a defined FITC gate (green fluorescence) by flow cytometry at steady state (10,000 cells counted per condition, n = 3 biological replicates).

Hal5 consists of a C-terminal kinase domain (amino acids 502–837) with a large N-terminal region (amino acids 1–501) that has not been characterized (**Fig 8A**). To test for features at the N-terminus of Hal5 that are important for function, we generated a truncation series deleting elements up to the beginning of the kinase domain (**Fig 8A**). Using this truncation series, we determined that *hal5Δ1–493* and *hal5Δ1–339* truncations failed to complement the growth defect in *hal* double mutant cells (**Fig 8B**) despite high levels of protein expression (**Fig 8C**). Furthermore, these truncations failed to complement aberrant Mup1 trafficking to the vacuole observed in *hal* double mutant cells (**Fig 8D–8F**). In contrast, *hal5Δ1–248* and *hal5Δ1–99* truncation variants fully complemented growth and trafficking phenotypes observed in *hal* double mutant cells (**Fig 8B** and **8D–8F**). Together, these data indicate that the kinase domain of Hal5 is not sufficient for regulation of endocytosis and that features of Hal5 upstream of the kinase domain are critical for the regulation of endocytosis.

### Features upstream of the kinase domain regulate Hal5 PM localization

**Fig 9:**
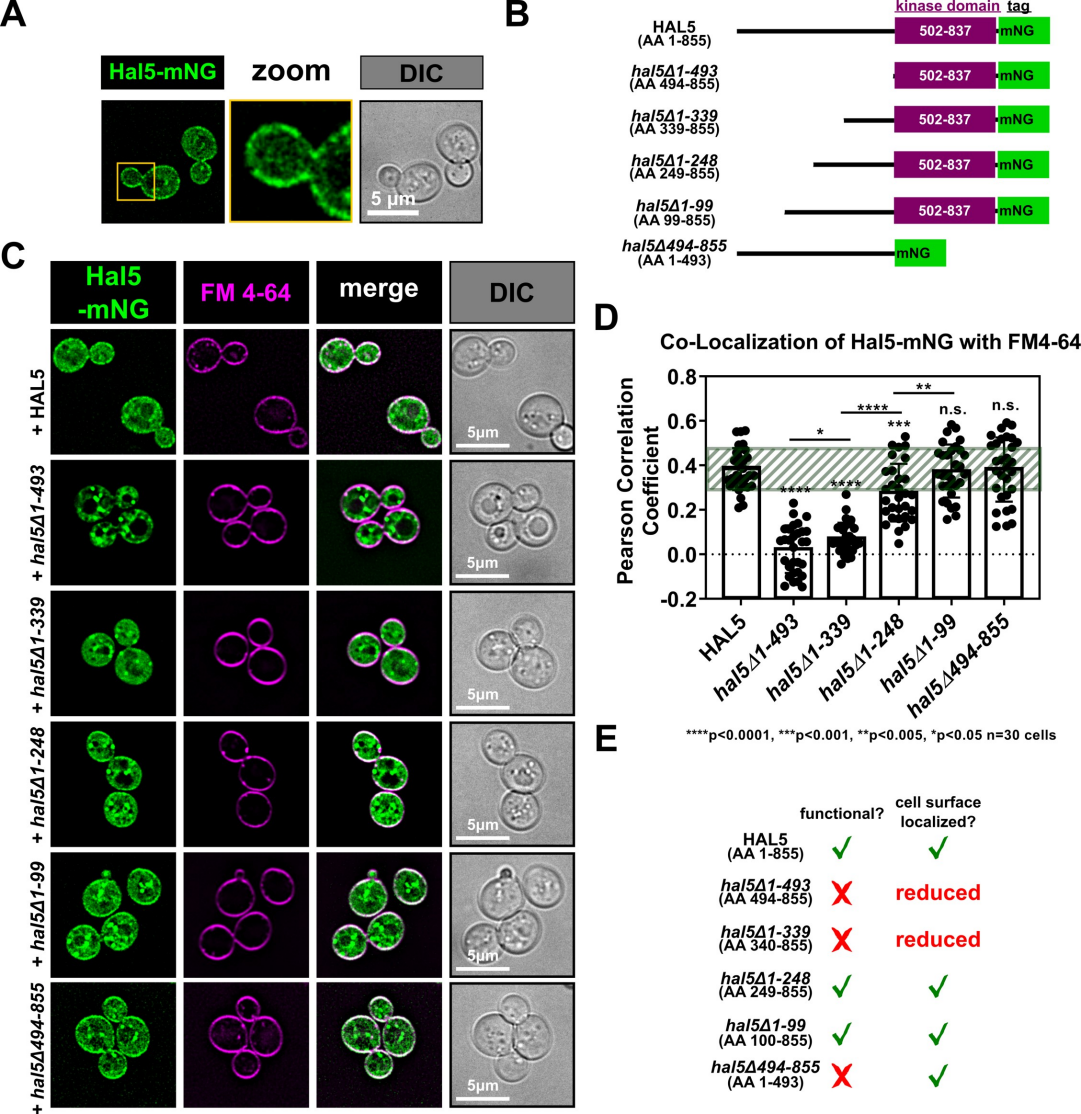
N-terminal elements of Hal5 are critical for localization to the PM. **(A)** Representative image of WT cells grown to mid-log phase in selective media expressing Hal5 C-terminally-tagged with mNeonGreen (Hal5-mNG) from a centromeric plasmid under native promoter control. **(B)** Schematic representation of C-terminally mNeonGreen-tagged Hal5 variants compared to WT Hal5. **(C)** Representative images of WT cells grown to mid-log phase in selective media expressing variants of Hal5-mNG after a brief FM 4–64 pulse to label PM immediately prior to imaging **(D)** Hal5 localization to the PM was quantified in (C) by measuring Pearson correlation coefficient of Hal5-mNG signal with FM 4–64 signal. Standard deviation of cells expressing full-length Hal5-mNG is denoted by the green box. **(E)** Table summarizing each Hal5 variant tested, its functionality, and its localization.

Based on the functional data provided in our truncation analysis (**Fig 8**) we hypothesized that N-terminal elements may contribute to Hal5 subcellular localization. Since Hal5 localization in cells has not been reported, we analyzed the subcellular localization of Hal5 tagged at the C-terminus with mNeonGreen (mNG, [65]) and observed cytosolic and peripheral localization (**Fig 9A** (exogenous expression from a CEN plasmid) and **S16 Fig** (endogenous expression)). Importantly, C-terminal tagging of Hal5 with mNG did not result in loss of function as assayed by growth complementation (**S16 Fig**). To test if peripheral Hal5 localized to the PM, we imaged Hal5-mNG in cells pulse-labelled with FM4-64 (a lipophilic tracer dye that incorporates into the bilayer of the PM [55]) and detected significant co-localization, indicating that the peripheral pool of Hal5 indeed localizes to the PM (**Fig 9B–9D**). To further characterize the PM-localized pool of Hal5, we imaged cells expressing Hal5-mNG along with mCherry-tagged endocytic site components including Ede1, Sla2, Ent1, and Abp1 [13] and found that Hal5 only occasionally co-localized with these structures (**S16 Fig**). Given that endocytic sites are highly dynamic, we cannot exclude the possibility that this limited co-localization may reflect a dynamic association between Hal5 and the endocytic machinery. Despite some co-localization of Hal5-mNG with Mup1-mCherry (which localizes generally to the PM), we did not detect significant co-localization of Hal5-mNG with mCherry-tagged variants of the Rsp5 adaptor Art1 or the eisosome component Pil1 (**S16 Fig**). Interestingly, treatment of cells with LatA increased the extent of Hal5 localization to the PM (**S17 Fig**), indicating that inhibition of endocytosis and/or actin dynamics stabilizes Hal5 at the PM.

To determine whether the N-terminal region of Hal5 contains features critical for PM localization, we analyzed the subcellular location of an N-terminal truncation series of Hal5 (**Fig 9B**) and found that deletion of amino acids 1–493 and 1–339 resulted in reduced detection at the PM, while truncations deleted for amino acids 1–248 and 1–99 were retained at the PM (**Fig 9B–9D**). Importantly, we also observed that deletion of the kinase domain did not alter PM localization–indicating that PM localization is determined by elements upstream of the kinase domain. As with full-length Hal5, treatment of cells with LatA increased the PM localization of the Hal5 N-terminal domain but had no effect on the localization of the kinase domain (**S17 Fig**). Thus, our data reveals a correlation between Hal5 function and localization of the kinase domain to the PM (**Fig 9E**), suggesting that recruitment of Hal5 kinase activity to the PM is critical for its regulation of endocytic trafficking.

### Nutrient availability regulates Hal5 localization

**Fig 10:**
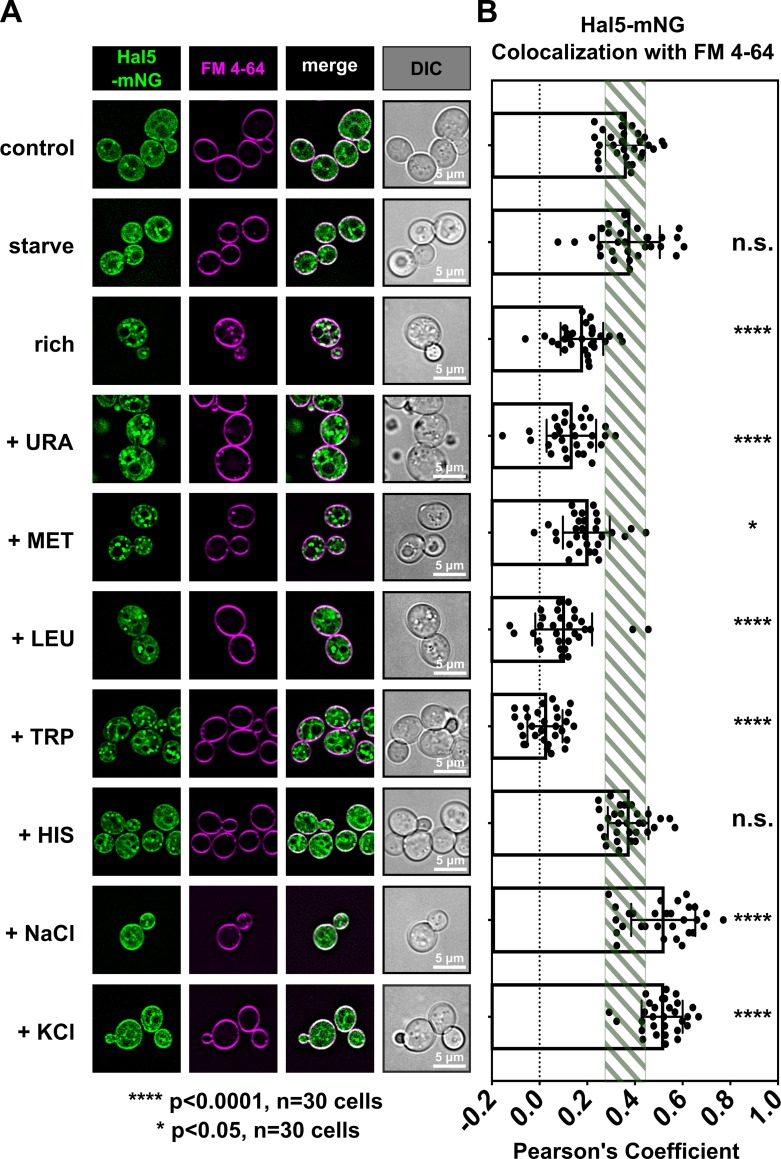
Nutrient availibility regulates Hal5 localization. **(A)** Representative images of WT cells expressing full-length Hal5-mNG from a centromeric plasmid under native promoter control. Cells were grown to mid-log phase in selective media then switched to media with the indicated nutrient conditions (10 μg/mL methionine (+ MET), or 10 μg/mL uracil (+ URA)) for 10 minutes, then briefly pulsed with FM 4–64 to label PM immediately prior to imaging. **(B)** Hal5 localization to the PM was quantified in (A) by measuring Pearson correlation coefficient of Hal5-mNG signal with FM 4–64 signal. Standard deviation of control cells expressing full-length Hal5-mNG is denoted by the green box.

We hypothesized that localization of Hal5 to the PM might be important for its regulation of endocytosis. We observed no effect of acute inhibition on the PM localization of Hal5AS (**S18 Fig**) indicating that Hal5 kinase activity and autophosphorylation do not regulate its PM association. We hypothesized that Hal5 might respond to environmental changes that trigger endocytic downregulation. To test this, we analyzed the subcellular localization of Hal5 (both the N-terminal domain (**S19 Fig**) and full-length Hal5 (**Fig 10**)) following exposure to environmental stimuli known to regulate nutrient transporter stability. First, we tested if shifting from minimal media (SCD) to rich media (YPD) or to starvation media (potassium-acetate-raffinose) altered Hal5 localization. No change in Hal5 localization was observed following a shift from minimal media to starvation media, while shifting to rich media resulted in reduced PM localization of Hal5 (**Fig 10A and 10B**). Based on this result, we hypothesized that specific excess nutrients in YPD might trigger reduced Hal5 PM localization. To test this, we analyzed localization of Hal5 in cells grown in minimal media and stimulated by addition of specific nutrients. We found that stimulation with uracil, methionine, leucine, or tryptophan resulted in reduced localization of Hal5 to the PM (**Fig 10A and 10B** and **S19 Fig**). Stimulation of cells with histidine had no effect on Hal5 localization, while increasing salt concentration increased Hal5 localization to the PM (**Fig 10A and 10B** and **S19 Fig**). Since stimulation with specific nutrients reduced Hal5 levels at the PM, we considered the possibility that Hal5 localization is regulated by TORC1 signaling. In contrast to Npr1, which is responsive to TORC1 signaling output [27], inhibiting TORC1 by treatment with rapamycin or activating TORC1 by treatment with cycloheximide did not result in any detectable changes in Hal5 localization or SDS-PAGE mobility (**S19 Fig**). Taken together, these data indicate that the presence of certain excess nutrients (uracil, methionine, leucine, and tryptophan, but not histidine) reduces Hal5 PM localization, although this response is distinct from the nutrient-sensing TORC1 signaling pathway.

## Discussion

Hal4 and Hal5 are Snf1-related kinases that are most similar to other members of this family that lack a conserved threonine in the activation loop of the kinase catalytic domain, like Npr1. By investigating the role of Hal kinases in stabilization of integral membrane proteins at the PM, we have determined that Hal kinases function to antagonize endocytosis and stabilize many different types of integral plasma membrane proteins. Specifically, we report that ***(i)*** Hal kinase regulation includes both Art1-mediated and Art1-independent endocytic events, ***(ii)*** both Hal5 kinase activity and elements in its uncharacterized N-terminal domain are critical for regulation of endocytosis, and ***(iii)*** Hal5 PM localization is responsive to changes in nutrient availability, with repletion of certain nutrients resulting in decreased Hal5 PM association. Despite their similarities to Npr1, our data indicate that Hal kinases are distinct from Npr1 both in terms of regulation and in terms of mechanism of action. Thus, investigating Hal kinases and other members of this family will likely lead to a broader understanding of how endocytosis and signaling events are coupled to nutrient sensing in order to coordinate complex physiological processes like the adaptive growth response.

### Classification of Snf1-related kinases in the yeast kinome

Our sequence analysis indicates that the family of Snf1-related kinases in yeast is similar to the human family of kinases related to AMPK. Mammalian kinases related to AMPK have been placed into two categories based on multiple criteria, including ***(i)*** degree of relatedness to AMPK, ***(ii)*** domain architecture and position of the catalytic domain, and ***(iii)*** functional regulation by phosphorylation of the activation loop by an upstream kinase activity [66, 67]. Specifically, human AMPK-related kinases (or ARKs) have a higher degree of homology with the AMPK kinase domain, exhibit an N-terminal catalytic domain often in combination with C-terminal accessory domains like UBA and KA1 domains, and require an activating phosphorylation event on a conserved threonine residue in the activation loop (usually by LKB1). By comparison, human Snf1-related kinases (or SRKs) are not as closely related to AMPK as ARKs, are not known to contain additional accessory domains, and are thought to be regulated differently from ARKs (based on the absence of an upstream consensus sequence for LKB1).

The family of yeast kinases related to Snf1 are poorly characterized, but there is mounting evidence that members of this family play roles in both nutrient sensing and in the regulation of endocytic trafficking. Ultimately, a better understanding of this family of kinases will require deeper functional analysis for more family members. In accordance with how the family of AMPK-related kinases have been classified in the human kinome, we propose similar criteria for classifying yeast kinases related to Snf1 into two groups (summarized in **S2 Table**). Specifically, based on the criteria used to classify human ARKs, we propose the following criteria for classification of yeast AMPK-related kinases (or yARKs): ***(i)*** >55% similarity to the catalytic domain of Snf1, ***(ii)*** a domain architecture with an N-terminal kinase catalytic domain and known accessory domains at the C-terminus, and ***(iii)*** the presence of a conserved threonine residue in the kinase domain activation loop. Broadly, the yARKs were predicted to be orthologous to human ARKs based on sequence (**S1 Table**). Consistent with this analysis, the yARKs Kin1 and Kin2 are known to be orthologous to the human MARK kinases (involved in regulation of cell polarity) based on sequence and functional analysis [68]. Additional studies will be needed to determine the extent of functional relatedness between remaining human and yeast ARK family members. By comparison, we propose the following criteria for classification of yeast Snf1-related kinases (ySRKs): ***(i)*** percent similarity with the Snf1 catalytic domain is >40% but <55%, ***(ii)*** kinase catalytic domains that occur in the middle or C-terminus of the protein, and ***(iii)*** the absence of a conserved threonine in the activation loop of the kinase domain. In contrast to yARKs, the ySRKs were predicted to be orthologous to human SRKs based on sequence (**S1 Table**). Ultimately, to refine our understanding of this important clade of kinases, functional characterization of more family members will be required to uncover additional biochemical features that define and distinguish the members of this family.

### Hal kinases antagonize the endocytosis of many different types of integral PM proteins

How eukaryotic cells regulate endocytic site assembly and cargo selection for endocytosis remains poorly understood [12–14]. Phospho-regulation is known to occur at different stages along the endocytic trafficking route, and can involve regulation of cargo selection [27, 64, 69], endocytic site assembly [70, 71], multi-vesicular body (MVB) sorting [72], or recycling from endosomes [73]. One well-characterized example of regulation of endocytic assemblies in yeast involves the casein kinase Hrr25 (CKδ/ε in mammalian cells) which localizes to the PM in a discrete punctate pattern [71]. Hrr25 was found to localize to endocytic sites and arrives concurrently with the early endocytic site protein Ede1, which is itself a substrate for the Hrr25 kinase [71]. Thus, Hrr25 is an example of a kinase that generally regulates endocytosis by direct phosphorylation of endocytic site components. Like Hrr25, Hal5 also localizes to the PM in a punctate pattern, but in contrast to Hrr25 we find that Hal5 does not exhibit significant co-localization with known endocytic site components (**S16 Fig**). Although we cannot exclude the possibility that Hal5 may regulate the assembly of endocytic machinery, our data suggests that Hal kinases operate upstream of endocytic site assembly and instead function in a manner that at least partially involves the activity of Art1.

The ART family of proteins are best characterized for their adaptor functions related to the recruitment of Rsp5 to specific cargo at the PM, resulting in ubiquitylation that directs cargo trafficking to the vacuole [1, 46, 74–79]. In contrast to the cargo-specific functions of associated with individual ART family members, Hal kinases appear to regulate a broad spectrum of cargo [39, 40]. Here, we report that *hal* mutant cells exhibit aberrant vacuolar trafficking of several major facilitator family nutrient transporters, an Nramp family ion transporter (Smf1), a P2-type proton pump (Pma1), and a cell wall integrity sensor (Wsc1) (summarized in **Table 1**). We also report that the aberrant vacuolar trafficking of some of these cargo (Mup1, Pma1, and Wsc1) are dependent (or partially-dependent) on Art1. In the case of Pma1, aberrant vacuolar trafficking previously reported to occur in v-ATPase mutants is Art1-independent but Art9-dependent [47]. Our finding that Pma1 vacuolar trafficking in *hal* mutant cells is Art1-dependent suggests a different recognition mechanism compared to that which occurs in v-ATPase mutant cells. We also observed that Wsc1 traffics to the vacuole with partial Art1-dependence in *hal* mutant cells. Since Wsc1 endocytosis is reported to be ubiquitin-independent [50, 51], our findings may indicate that the role of Art1 in Wsc1 vacuolar trafficking is unrelated to its function as an adaptor for Rsp5. Indeed, one previous study reported a role for Art1, Art3 (Aly2) and Art6 (Aly1) in the clathrin-independent endocytosis of the pheromone receptor Ste3, and Rsp5 interaction was found to be dispensable for this function [76]. Furthermore, Art7 was reported to target the endocytic downregulation of the Ste2 pheromone receptor in the absence of Rsp5 engagement [77]. Consistent with such Rsp5-independent modes of action, we observed that ubiquitin-independent endocytosis of Wsc1 partially requires Art1 in *hal* mutant cells. However, further experimentation will be required to rigorously test if Art1-mediated Wsc1 trafficking in *hal* mutants requires the recruitment and activity of Rsp5.

Given the broad spectrum of cargo affected by the loss of Hal kinases, it is not surprising that many of the cargo examined in this study (Fur4, Can1, and Smf1) traffic to the vacuole independently of Art1 in *hal* mutant cells. We hypothesize that other arrestin-related Rsp5 adaptors may contribute to the aberrant trafficking phenotypes observed in *hal* mutants. Indeed, the aberrant trafficking of diverse cargo observed *hal* mutant cells is consistent with general activation of the broader ART-Rsp5 network, not just Art1. Mechanistically, it remains unclear how acute loss of Hal kinase activity triggers hyper-activation of the ART-Rsp5 network. One possible mechanism of action for Hal kinases could involve direct phosphorylation of endocytic cargo at the PM. One such example involves regulation of the essential yeast plasma membrane proton pump, Pma1, by the kinase Ptk2 [32, 33, 35], which qualifies as a ySRK using the criteria proposed here. Ptk2 localizes to the PM and phosphorylates serine 899 of the C-terminal tail of Pma1 to activate it in response to glucose stimulation [32]. The C-terminal cytoplasmic tail of Pma1 has reported roles in secretory trafficking, stability at the PM, and glucose-activated enzymatic activity [34] so phosphorylation at Ser899 could impact any of these processes. Given the diverse portfolio of PM proteins subject to Hal kinase regulation, a model involving direct phosphorylation of cargo by Hal kinases at the PM would require the activity to lack specificity.

Alternatively, Hal kinases may broadly inhibit endocytosis of many different types of PM proteins by controlling plasma membrane organization. The eukaryotic PM is often described as having a patchwork organization of many different microdomains, each with a unique identity and distinct function marked by enrichment in particular proteins and lipids [80–82]. For example, eisosomes are endocytosis-resistant microdomains of the yeast PM that protect cargo from access by ubiquitylation machinery [6, 83–86]. Some transporters like Can1 and Fur4 organize into eisosomes in the absence of substrate (arginine and uracil, respectively) while stimulation of cells with excess substrate triggers re-organization of nutrient transporters away from eisosomes [6, 86]. This movement may depend on conformational changes induced by substrate binding, as mutations that prevent conformational changes during substrate transport result in increased association with eisosome compartments [86]. Interestingly, structural components of the eisosome undergo extensive phosphorylation, but the physiological significance of such phosphorylation events is not currently understood [82, 86–88]. We did not observe appreciable co-localization of Hal5 with eisosomes (**S16 Fig**) or disruption of eisosome microdomains in *hal* mutant cells (**S4 Fig**), however we cannot exclude the possibility that Hal kinases may antagonize endocytosis by regulating the function of eisosomes at the PM.

Another possible explanation for the phenotypes observed in *hal* mutant cells is that Hal kinases may function to regulate some aspect of PM physiology (e.g., regulation of lipid composition) that broadly impacts the folding of integral membrane proteins at the PM. Indeed, proteotoxic stresses (e.g., heat stress, oxidative stress, etc.) are known to trigger endocytosis of a diverse assortment of PM proteins [74], not unlike what we report here for *hal* mutant cells. Moreover, the induction of endocytosis reported during proteotoxic stress is partly dependent on Art1 and partly dependent on other adaptors in the Rsp5 network [74]. We hypothesize that loss of Hal kinases triggers some alteration to the PM environment that results in misfolding of many different types of PM proteins, thus activating endocytic clearance driven by the PM quality control pathway. Alternatively, metabolic switches in yeast are known to trigger endocytic downregulation of PM proteins, and could also account for the broad PM remodeling observed in *hal* mutant cells. Examples of such metabolic switch-dependent PM remodeling have been reported with glucose signaling [18–20] and TORC1 signaling [27, 31]. Thus, it is possible that loss of Hal kinase activity triggers a metabolic switch that results in endocytic downregulation and PM remodeling. Indeed, *hal* mutants have been reported to exhibit decreased glucose uptake and increased mitochondrial activity relative to wildtype cells [40], but additional experimentation will be required to determine if these metabolic phenotypes are the cause or effect of PM remodeling.

### Hal kinases are PM-localized nutrient-responsive trafficking regulators

Our localization of Hal5 to the PM (**Fig 9A–9C**) and characterization of its response to changing environmental conditions (**Fig 10** and **S19 Fig**) suggests that Hal5 kinase activity at the PM may be inhibited upon changes in the availability of specific nutrients, like methionine and uracil. TORC1 (mTORC1) is a well-characterized nutrient-sensing kinase that signals from the limiting membrane of the vacuole to control catabolic and anabolic decisions (including protein synthesis or autophagy) in response to availability of specific nutrients including nitrogen, glucose, and lipids [89]. Despite a significant cytosolic population of mTORC1, and a relatively small fraction of mTORC1 localized to lysosomal membranes, it is thought that most mTORC1 kinase activity occurs at the lysosomal/vacuolar membrane [90]. High cytoplasmic amino acid concentrations (mainly leucine, arginine, and glutamine) promote the ability of mTORC1 to localize to lysosomal/vacuolar membranes [91–93]. Our data suggests that Hal5 may be regulated in a manner analogous to TORC1, since nutrient availability affects its ability to localize to the PM.

Despite the spatial restriction of active mTORC1 in mammalian cells (as well as yeast), TORC1 has recently been demonstrated to exert regulatory control over endocytosis in yeast. One way in which it does this is through negative regulation of Npr1 (a ySRK) to regulate composition of the PM proteome, and therefore nutrient influx [27, 28, 30, 31]. Importantly, Npr1 is thought to antagonize endocytosis at the level of specific Rsp5 adaptor proteins, including Art1, Bul1, and Bul2, and therefore regulate a specific subset of endocytic cargo [27, 30, 31]. Surprisingly, previous reports indicate that overexpression of Npr1 improves *hal* mutant growth defects through stabilization of nutrient transporters at the PM [94], suggesting that Npr1 may compensate for some Hal functions. However, our data indicates that Hal kinases do not function to regulate Art1 phosphorylation or localization, suggesting that Hal kinases and Npr1 function via distinct mechanisms. Additional experimentation will be required to determine if Hal kinases regulate other players in the ART-Rsp5 network. Given the similarities between Hal kinases and Npr1, we predict that other members of the ySRK family may also function to regulate endocytosis (or other membrane trafficking events) in response to changing environmental conditions.

### Regulation of Hal kinases by elements in the N-terminal domains

The ySRK family of kinases shares conservation restricted to kinase catalytic domains (**Fig 2**). Outside of the catalytic domains, sequences are divergent. For example, the N-terminal regions of Hal4 and Hal5 do not exhibit a considerable degree of sequence conservation with each other or other family members, which have been largely uncharacterized. Snf1 (AMPK in mammalian cells), the best characterized nutrient-sensing kinase that regulates endocytosis, exists as a catalytic subunit (α) in a heterotrimeric complex that is autoinhibited, and this autoinhibition is released as glucose is depleted [15]. Other members of this heterotrimeric complex include Snf4, an invariant stimulatory subunit (γ), and a variable (β) subunit which consists of either Sip1, Sip2, or Gal83 [15]. In Snf1 complexes, the β subunit confers specificity to activated Snf1 by targeting subcellular localization and mediating substrate interactions [16, 95]. Interestingly, inactive Snf1 in complex with any one of the three β subunits localizes to the cytosol, while active Snf1 in complex with Sip1 re-localizes to the vacuole and association with Gal83 re-localizes active Snf1 to the nucleus [16]. Active Snf1 association with Sip2 remains localized to the cytosol [16]. Thus, the nutrient-sensing capabilities, subcellular localization, and substrate interactions of Snf1 are governed by its association with different subunits *in trans*. Our data suggests that nutrient-responsiveness and subcellular localization of Hal5 are controlled by its N-terminal region *in cis*. We propose that regulation of Hal5 activity may be similar to regulation of Snf1, except that *cis*-acting elements in the N-terminus of Hal5 may function in regulation of localization or substrate selection, analogous to the β subunits of Snf1. Alternatively, it is possible that Hal kinases may interact with regulatory subunits that contribute to its regulation or localization, although such interacting proteins are yet to be identified. In contrast to Snf1, it is currently unknown if Hal kinases are capable of directly sensing nutrients or if they indirectly respond to changes in nutrient availability, as has been established for the Npr1 kinase.

Considering the divergence amongst N-terminal domains in ySRK family members, we speculate that the N-terminal regions confer substrate targeting and subcellular localization of these kinases similar to the accessory subunits of the heterotrimeric Snf1 complexes. Consistent with this hypothesis, active Npr1 localizes to the PM, and inhibition of TORC1 signaling with rapamycin treatment alters both Npr1 localization as well as phosphorylation events in the N-terminal region [25–27]. In addition to those identified in this study, high-throughput proteomics studies have identified multiple phosphorylation events on Hal5 that occur throughout the N-terminal region [96–99], but the physiological significance of those events is not currently understood. Thus, similar to Npr1, it is possible that Hal kinases are regulated by phosphorylation events that occur within the N-terminal domain, and such phosphorylation may impact the ability of Hal5 to localize to the PM. Interestingly, Hal4 is predicted to have a mitochondrial targeting sequence and has been found to sediment in mitochondrial subcellular fractions [100]. In contrast, Hal5 is not predicted to have a mitochondrial targeting sequence, and we speculate that differential subcellular localization may be critical for coordinating Hal4 and Hal5 activities. Furthermore, differential subcellular localization of Hal4 and Hal5 may explain why their functions appear to be distinct in some cases (**S5 Fig**). Ultimately, characterizing the subcellular localizations and activities of the broader family of Snf1-related kinases, including Hal4 and Hal5, will contribute to the overall understanding of the regulation of endocytosis and will provide insights into how cells sense and respond to environmental cues.

## Materials and methods

### Plasmids, Yeast strains and culturing conditions

The *SEY6210* strain background (*MATα leu2-3*,*112 ura3-52 his3-Δ200 trp1-Δ901 lys2-801 suc2-Δ9)* was used for most experiments. Genomic tagging and deletion of genes was performed using standard PCR-based homologous recombination, as described previously [1]. Strains with multiple genomic modifications (genomic tags, deletions, or some combination) were generated by mating, sporulation (potassium acetate raffinose media), and subsequent tetrad dissection using a tetrad dissection microscope (MSM System 400, Singer Instruments). For determination of growth phenotypes, yeast cells were cultured in indicated media (SC or YP + dextrose liquid media) overnight at 26°C. 1 OD_600_ equivalent was harvested, serially diluted into sterile water, and plated onto indicated media (SC or YP + dextrose solid media, either control of treated) using a 48-well metal replica plater (Sigma). See **S3 Table** for a list of strains used in this study.

Unless otherwise indicated, all genes were cloned with native promoter sequence from genomic yeast DNA using standard PCR methods, restriction digest and ligation into centromeric (pRS) vector backbones. Constructs with point mutations were generated using PCR site-directed mutagenesis. All constructs generated by PCR in this study were verified by sequencing. See **S4 Table** for a list of plasmids used in this study.

### Fluorescence microscopy analysis of cargo trafficking and Hal localization

Protein trafficking and localization analyses were performed by growing yeast cells expressing fluorescent fusion proteins (GFP, mNG, MARS, or mCherry) to mid-log phase in indicated synthetic liquid media at 26°C and imaged live in synthetic liquid media using a DeltaVision Elite Imaging system (Olympus IX-71 inverted microscope; Olympus 100× oil objective (1.4 NA); DV Elite sCMOS camera, GE Healthcare). In experiments using FM 4–64 as a PM label, cells were incubated on ice for 5 minutes, then spotted onto a slide and mixed with FM 4–64 (final concentration of 12.5x or 125 μg/mL) and imaged within 10 minutes. In experiments examining cargo-GFP trafficking in response to treatment with 1-NA-PP1 (Adooq Bioscience, Irvine, CA), cells were treated for 1 hour and imaged in the same media. In experiments examining Hal5-mNG localization in response to 1-NA-PP1 treatment, cells were treated for 10 minutes, and placed on ice for 5 minutes prior to imaging. In experiments examining Hal5-mNG localization in response to nutrients, cells were either resuspended in a starvation media (potassium-acetate raffinose media), or excess nutrients were added to the media (10μg/mL uracil, 10μg/mL methionine, 500mM NaCl, or 300mM KCl). After 10 minutes of treatment, cultures were placed on ice for 5 minutes prior to imaging. Images were collected and deconvolved, then quantified. The PM:Vac ratio analysis (S5D Fig) was performed as previously described [64]. Specifically, Softworx image analysis software was used to measure the fluorescence signal intensity at the PM and in the vacuole and PM:Vac ratios were computed for a large number of cells (n = 50). In cases where some cells contained no detectable localization to the PM, binning analysis was performed (Figs 3, 4, 5, 7 and 8). Specifically, cells were counted and grouped (binned) into categories defined by the localization of Mup1-GFP, Can1-GFP, or Fur4-GFP. Pearson correlation coefficients were determined by drawing a region of interest around each cell and using the Pearson correlation coefficient function using Softworx software (GE Healthcare). Images were pseudo-colored using the free open-source program Fiji.

### Analysis of endocytic recycling by measurement of FM4-64 efflux

FM 4–64 efflux was measured by growing yeast cells were grown to mid-log phase in liquid media (YP + dextrose) at 30°C then shifted to 22°C for 10 minutes. Cells were pulsed with FM 4–64 (1x or 10μg/mL) for 8 minutes at 22°C then placed on ice for 10 minutes. Cells were washed with ice-cold liquid media (SM + dextrose) three times. Cells were distributed into a 96-well plate in 250 μl aliquots while on ice, and then warmed to room temperature for 3 minutes prior to analysis by flow cytometry using a BD Accuri C6 Plus benchtop flow cytometer (BD Biosciences). Over 10,000 cells per time point were detected and analyzed per condition based on gating in the PE channel, which detects signal from FM 4–64.

### Analysis of Mup1-pHluorin trafficking

Analysis of Mup1-pHluorin trafficking and steady state surface abundance was performed as previously described [64]. Briefly, Mup1-pHluorin trafficking was examined at steady-state or over time in response to stimulus (either 2μg/mL methionine or 26.3μM 1-NA-PP1) by growing yeast cells to mid-log phase in indicated synthetic liquid media at 26°C. Cells were distributed into a 96-well plate in 250 μl aliquots prior to analysis by flow cytometry using a Guava easyCyte benchtop flow cytometer (Millipore). Over 10,000 cells per time point were detected and analyzed per condition based on gating in the FITC channel, which detects signal from pHluorin.

### Analysis of protein expression in cultured yeast cells

Yeast lysates were prepared from mid-log phase cultures grown in the indicated selective synthetic liquid media at 26°C. 5 OD_600_ equivalents were precipitated in 10% trichloroacetic acid (TCA) in TE (10 mM Tris-HCl, 1mM EDTA, pH 8.0) and subsequently washed with acetone, aspirated, dried under vacuum, solubilized in lysis buffer (150 mM NaCl, 50 mM Tris pH7.5, 1 mM EDTA, 1% SDS) and disrupted by vortex with 100 μL of acid-washed glass beads. Urea-sample buffer (150 mM Tris pH 6.8, 6 M Urea, 6% SDS, 10% β-mercaptoethanol, 20% glycerol) was added and samples were heated to 65°C prior to analysis by SDS-PAGE and subsequent immunoblotting. SDS-PAGE gels were transferred to a polyvinylidene fluoride (PVDF) membrane (Immobilon-FL; 0.45μM pore-size; MilliporeSigma), and blocked using 5% milk in TBST (tris buffered saline with tween-20; 10mM tris-HCl, 150 mM NaCl, 0.05% tween-20, pH 7.5). Membranes were incubated with primary antibodies α-FLAG (M2; mouse monoclonal; Sigma; used at 1:2000 dilution) and/or α-G6PDH (rabbit polyclonal; Sigma; used at 1:20,000 dilution), washed using TBST, and incubated with fluorescently labeled secondary antibodies (LI-COR Biosciences; IRDye 680RD Goat anti-Mouse IgG and IRDye 800CW Goat anti-Rabbit IgG; used at 1:10,000 dilution). Fluorescent imaging of immunoblots was performed using an Odyssey infrared imaging system (LI-COR Biosciences) and quantified using the proprietary Odyssey software LI-COR Image Studio (LI-COR Biosciences).

### Analysis of Art1 and Hal5 phosphorylation by SILAC-MS

Quantitative mass spectrometry analysis of Art1-FLAG and Hal5-FLAG by SILAC-MS was performed as previously described [64]. Briefly, lysates were generated from yeast cultures labelled with heavy or light arginine and lysine and FLAG-tagged bait proteins (Art1-FLAG or Hal5-FLAG) were purified using EZView M2 FLAG agarose beads (Sigma). After washing, baits were eluted from beads by boiling in 10% SDS and eluates were collected and precipitated by addition of 50% ethanol, 49.9% acetone and 0.1% acetic acid. Protein pellets were resuspended in 20μL of 8M urea/50mM Tris (pH 8.0) and the suspension was diluted by addition of 50μL of water and digested overnight with 1μg trypsin (Gold, Promega). Resulting phosphopeptides were enriched using immobilized metal affinity chromatography [27] and analyzed on a Q Exactive mass spectrometer (Thermo). Resulting spectra were searched using MaxQuant software (ver. 1.5.3.30) and chromatography was analyzed using Skyline software (MacCoss Lab).

### Bioinformatic analysis

Protein kinase sequences were retrieved from *Saccharomyces* Genome Database (SGD, https://www.yeastgenome.org) and aligned using Clustal Omega (EMBL-EBI, multiple sequence alignment) or EMBOSS Water (EMBL-EBI, pairwise sequence alignment) [41]. Sequence alignments were visualized using iTOL (https://itol.ebl.de.itol.cgi) [101], EvolView v3 (www.evolgenius.info/evolview.html) [102], or JalView (www.jalview.org) [103]. SGD YeastMine (https://yeastmine.yeastgenome.org/yeastmine) was used to search and retrieve *S*. *cerevisiae* data, populated by SGD based on a curated list of protein kinases. Data retrieved through YeastMine for this study includes the number of publications annotated for each protein kinase (as of April 29, 2019) and orthologous across several model organisms. Information about protein kinase domains and architecture was retrieved automatically through the EvolView interface from UniProt (EMBL-EBI, https://www.uniprot.org). Secondary structure prediction of Hal5 was performed using JPred [104]. A pairwise sequence alignment of Hal5 and Snf1 catalytic domains was used to generate a structural model for the Hal5 catalytic domain using MODELLER (https://salilab.org/modeller/) [105] through the Chimera interface (UCSF, https://www.cgi.ucsf.edu/chimera/) [106].

## Supporting information

S1 Fig A multiple sequence alignment of all 130 known protein kinases in yeast was performed using Clustal Omega and visualized as a scaled, unrooted phylogenetic tree using iTOL. The protein kinases cluster into 6 major clades, which have been arbitrarily numbered and color-coded for simplicity and ease of viewing across different figures. The 5^th^ clade in teal contains Snf1. Kinases clustering with Snf1 include many kinases originally described as the NPR/HAL5 family, denoted by black asterisks (PTK1, PTK2, NPR1, PRR2, RTK1, HRK1, HAL5, KKQ8, and HAL4) GCN2 and CHK1, also originally described as NPR/HAL5 family members, are clustering with groups 3 and 6, respectively.
(TIF)

S2 Fig Every predicted ortholog for kinases clustering with Snf1 by multiple sequence alignment was tallied and identified as AMPK (red), AMPK-related (ARKs, orange), Snf1-related (SRKs, blue), or Other (black).
(TIF)

S3 Fig A multiple sequence alignment, performed using Clustal Omega and visualized in JalView, of the activation loops (DFG…APE) in kinases clustering with Snf1 by phylogenetic analysis. The amino acid position aligning with T210, critical threonine of the Snf1 activation loop [108], is denoted by the black indicator.
(TIF)

S4 Fig **(A)** Representative images of Can11-GFP expressed from a centromeric plasmid under native promoter control in the presence of endogenously MARS tagged Vph1, a marker for the limiting membrane of the vacuole. WT, *Δhal4Δhal5* cells, or *Δhal4Δhal5Δart1* cells were cultured to mid-log phase in selective media. **(B)** Quantification of Can1-GFP localization in (A) performed by binning cells into localization categories as indicated. **(C)** Representative images of Smf1-GFP expressed from a centromeric plasmid under native promoter control in the presence of endogenously MARS tagged Vph1, a marker for the limiting membrane of the vacuole. WT, *Δhal4Δhal5* cells, or *Δhal4Δhal5Δart1* cells were cultured to mid-log phase in selective media. **(D)** Quantification of Smf1-GFP localization in (C) performed by binning cells into localization categories as indicated. **(E)** Representative images of Pil1-GFP expressed from a centromeric plasmid under native promoter control in the presence of endogenously-tagged Vph1-MARS, a marker for the limiting membrane of the vacuole. WT and *Δhal4*,*5* mutant cells were imaged after being cultured to mid-log phase in selective media. **(F)** Representative images of Snc1-GFP expressed from a centromeric plasmid under native promoter control in WT and *Δhal4*,*5* cells in the presence of endogenously MARS tagged Vph1, a marker for the limiting membrane of the vacuole. **(G)** Percentage of cell population positive for FM 4–64 fluorescence as measured by cells that fall within a defined PE gate (red fluorescence) as measured by flow cytometry (10,000 cells counted per condition, n = 3 biological replicates) in WT, *Δhal4*,*5*, *or Δrcy1* cell populations (grown to mid-log phase in rich media). This assay is an indirect measure of endosomal lipid recycling by monitoring loss of membrane-bound FM 4–64 due to efflux into the media over time. **(H)** Representative images of Cps1-GFP under conditions previously described in (C). **(I)** Representative image of cells serially diluted on synthetic complete media and grown for 3 days to assess growth of various mutants.
(TIF)

S5 Fig **(A)** Representative image of cells serially diluted on synthetic complete media and grown for 3 days to assess growth of various *hal* mutants **(B)** Growth of cells seeded at 0.05 OD from mid-log phase and monitored over time for OD_600nm_ in synthetic complete liquid media. **(C)** Representative images of Can11-GFP expressed from a centromeric plasmid under native promoter control in the presence of endogenously MARS tagged Vph1, a marker for the limiting membrane of the vacuole. WT and *Δhal4* or *Δhal5* single mutant cells were cultured to mid-log phase in selective media. **(D)** Quantification of Can1-GFP localization in (C) was performed by measuring the ratio of GFP signal at the PM compared to the vacuole (PM:VAC). Double mutants are excluded from this analysis due to lack of signal at the PM. **(E)** Representative image of indicated cells serially diluted on synthetic complete media to assess sensitivity (or resistance) to growth in the presence of the indicated concentration of canavanine, a toxic arginine analog. **(F)** Representative image of cells serially diluted onto indicated media and grown for 3 (YPD) or 5 (SCD) days to assess growth of Δ*hal4* and Δ*hal5* single mutants under Tunicamycin, an ER protein folding stress, low glucose (0.2% glucose compared to 2% in control), manganese, lithium, or caffeine stresses.
(TIF)

S6 Fig **(A)** A pairwise sequence alignment, performed using EMBOSS (EMBL-EBI) and visualized using JalView, of the Hal5 and Snf1 catalytic domains to identify important conserved residues of Hal5 (including K546, M620, and D688 as well as lack of a conserved threonine in the activation loop at Snf1 T210) **(B)** The pairwise alignment of Snf1 and Hal5 catalytic domains was then used to model Hal5 (pink) onto Snf1(cyan) structure using MODELLER through the Chimera interface. In the panel at the top-right is a zoomed-in view of the conserved catalytic aspartate residues in the active sites. In the panel at the bottom-right is a zoomed-in view of the conserved ATP-coordinating lysine residues (in red) and the gatekeeper residues (in light blue) in the ATP-binding pockets.
(TIF)

S7 Fig *Δhal5* mutant cells expressing endogenously-tagged Mup1-pHluorin and exogenously expressed (A) native Hal5 (HAL5), (B) C-terminally-tagged Hal5 (HAL5-HTF) or (C) C-terminally-tagged catalytic dead Hal5 (D688A-HTF). Percentage of cell population expressing endogenously tagged Mup1-pHluorin as measured by cells that fall within a defined FITC gate (green fluorescence) by flow cytometry (10,000 cells counted per condition, n = 3 biological replicates) over time in response to methionine, an endocytic stimulant. Mup1-pH PM half-time (t1/2) estimated based on initial and final time points and elapsed time.
(TIF)

S8 Fig **(A)** Representative images of Mup1-GFP expressed from a centromeric plasmid in the presence of endogenously MARS tagged Vph1, a marker for the limiting membrane of the vacuole. HAL5 is exogenously expressed in the absence of endogenous Hal5 from a centromeric plasmid under native promoter control with either no tag (HAL5), a C-terminal 6xHIS-TEV-3xFLAG tag (HAL5-HTF), or a C-terminally-tagged catalytic dead variant (*D688A-*HTF). **(B)** Quantification of (A) by measuring the ratio of Mup1-GFP signal at the PM compared to the vacuole (PM:VAC). **(C)** Immunoblot analysis of C-terminally-tagged Hal5 variants described in (A) as well as an additional C-terminally-tagged catalytic dead variant (*hal5*-*K546R-HTF*).
(TIF)

S9 Fig Quantitative immunoblot analysis, with a representative immunoblot, of WT, *Δhal4*, *Δhal5*, or *Δhal4*,*5* cells expressing Art1 endogenously tagged with 6X-HIS-TEV-3XFLAG at the c-terminus (ART1-HTF). Total art1 (orange bars) was quantified by measuring signal of both bands corresponding to Art1, and normalizing to G6PDH levels. Unmodified Art1 (yellow bars) was quantified by measuring signal of the bottom band, and normalizing to G6PDH levels. Ubiquitin-modified (Ub modified) Art1 (gray bars) was quantified by measuring signal of the top band, and normalizing to G6PDH levels. N = 3
(TIF)

S10 Fig **(A)** Schematic of the domain architecture of the Art1 protein, with known phosphorylation sites indicated. **(B)** Description of SILAC-MS experiments performed to profile Art1 phosphorylation with and without Hal4 and Hal5 kinase activity. **(C-L)** Analysis of experiments described in **(B)**. **(C,F,I)** 2% of prepared samples were analyzed by immunoblot to confirm Art1-HTF bait purification. Samples were subsequently submitted for mass spectrometry analysis. **(D,G,K)** The H:L ratio for all peptides of the indicated proteins were averaged to compute a measurement of the H:L ratio for the protein in the indicated experiment. Additional cell material was collected from *hal* mutant cells to compensate for the observed loss in Art1 abundance (S5 Fig) (i.e. 1L of 0.5 OD WT cells vs 0.75 OD *hal* cells). **(E,H,L)** The LOG_2_(H:L ratio) (normalized to total Art1) for each phosphorylation event detected is plotted and color-coded to correspond to the region of the Art1 protein as indicated in (A). **(J)** For experiment #3, following treatment of cultures with 1-NA-PP1 (and just prior to sample collection) 10mL of each culture was removed from the sample and cultured for an additional 24 hours in order to confirm inhibition by the compound.
(TIF)

S11 Fig Chromatography data used for quantification of phosphopeptides from Experiment #1 described in S6B Fig. Filtered chromatography data is shown for the indicated peptides (light peptide in red and heavy peptide in blue). Phosphopeptides identifying phosphorylation at Thr93 (top right) and Thr795 (bottom right) and the corresponding unmodified peptides (top left and bottom left, respectively) are depicted.
(TIF)

S12 Fig Chromatography data used for quantification of phosphopeptides from Experiment #2 described in S6B Fig. Filtered chromatography data is shown for the indicated peptides (light peptide in red and heavy peptide in blue). Phosphopeptides identifying phosphorylation at Thr93 (top right) and Thr795 (bottom right) and the corresponding unmodified peptides (top left and bottom left, respectively) are depicted.
(TIF)

S13 Fig Chromatography data used for quantification of phosphopeptides from Experiment #3 described in S6B Fig. Filtered chromatography data is shown for the indicated peptides (light peptide in red and heavy peptide in blue). Phosphopeptides identifying phosphorylation at Thr93 (top right) and Thr795 (bottom right) and the corresponding unmodified peptides (top left and bottom left, respectively) are depicted.
(TIF)

S14 Fig (A) Representative images of WT, *Δhal4*,*5*, *Δnpr1*, cells expressing Art1-mNG from a plasmid were grown to mid-log phase in selective media and imaged after a brief FM 4–64 pulse to label PM immediately prior to imaging. In the far right column of the panel, WT cells were treated with methionine (2μg/mL) for 10 minutes prior to FM 4–64 pulse. **(B)** Art1 localization to the PM in (A) was quantified by measuring Pearson’s correlation coefficient of Hal5-mNG signal with FM 4–64 signal.
(TIF)

S15 Fig Analysis of Hal5 phosphorylation from data collected in Experiment #3 from S6 Fig. **(A)** Quantitative phosphoprofiling analysis Hal5 based on SILAC-MS data. Schematic of the domain architecture of Hal5 is shown at the top. **(B)** Fingerprinting and quantification of individual phosphorylation events resolved for Hal5. MS2 spectra for individual phosphopeptides (left panels) and filtered chromatograms for quantification of light (red) and heavy (blue) peptides (right panels) are shown for Ser353 (top), Ser358 (middle) and Ser395 (bottom).
(TIF)

S16 Fig **(A)** Representative images of cells expressing endogenously-tagged Hal5-mNG grown to mid-log phase in rich media imaged near the cell middle (left) or cell surface (right) to asses Hal5 localization. **(B)** Cells expressing either empty vector (ev) or Hal5-mNG serially diluted onto synthetic selective media and grown for 5 days to assess functionality of C-terminally-tagged Hal5-mNG. **(C)** Cells co-expressing endogenous Hal5-mNG and mCherry-tagged components of endocytic site machinery corresponding to either early (top), mid/late (middle), or invagination (bottom) events. **(D)** Cells co-expressing endogenous Hal5-mNG and mCherry-tagged Pil1 (eisosomes), Mup1 (nutrient transporter, broad PM marker), or Art1 (Rsp5 adaptor).
(TIF)

S17 Fig **(A)** Representative images of WT cells grown to mid-log phase in selective media expressing Hal5-mNG (with or without 1 hour LatA treatment) after brief FM 4–64 pulse to label PM immediately prior to imaging **(B)** Localization of Hal5 to the PM was quantified in (A) by measuring Pearson correlation coefficient of Hal5-mNG signal with FM 4–64 signal. **(C)** Representative images using conditions described in (A) for WT cells expressing either a Hal5 variant deleted for the N-terminal region (*hal5Δ1-493-mNG*) or a Hal5 variant deleted for the kinase domain (*hal5Δ494-855-mNG*). **(D)** Quantification of Hal5 localization to the PM in (C) performed as described in (B).
(TIF)

S18 Fig **(A)** Representative images of WT cells expressing WT or analog-sensitive variants of Hal5-mNG (wt or *hal5AS*) from a centromeric plasmid under native promoter control. Cells were grown to mid-log phase in selective media. Cells were untreated (control), treated with vehicle (DMSO), or inhibitor (1-NA-PP1 26.3μM) for 10 minutes, then briefly pulsed with FM 4–64 to label PM immediately prior to imaging. **(B)** Hal5 localization to the PM was quantified in (A) by measuring Pearson correlation coefficient of Hal5-mNG signal with FM 4–64 signal. Standard deviation of control WT cells denoted by green box.
(TIF)

S19 Fig **(A)** Representative images of WT cells expressing a C-terminally mNG-tagged variant of Hal5 deleted for the kinase domain from a centromeric plasmid under native promoter control. Cells were grown to mid-log phase in selective media then switched to media with the indicated nutrient conditions (10 μg/mL methionine (+ MET), 10 μg/mL uracil (+URA)) for 10 minutes, then briefly pulsed with FM 4–64 to label PM immediately prior to imaging. **(B)** Hal5 localization to the PM was quantified in (A) by measuring Pearson correlation coefficient of Hal5-mNG signal with FM 4–64 signal. Standard deviation of WT cells is denoted by the green box. **(C)** Representative images of WT cells expressing full-length Hal5-mNG as described in (A). Prior to pulsing with FM 4–64 to label the PM, cells were treated with either DMSO (mock), 50 μg/mL of cycloheximide (+ CHX) or 200 ng/mL of rapamycin (+ RAP) for 15 minutes. **(D)** Quantification of (C) as described in (B). **(E)** Immunoblot analysis of whole cell lysates collected from WT cells expressing full-length c-terminally tagged Hal5-HTF, treated with either DMSO (mock), Cycloheximide (50 μg/mL), or Rapamycin (200 ng/mL) for 15 minutes. EV indicates empty vector.
(TIF)

S1 Table SGD YeastMine was used to search and retrieve *S*. *cerevisiae* data, populated by SGD and powered by InterMine by using a gene list of kinases clustering with Snf1 to predict orthologs across evolution. Red text = predicted AMPK ortholog, blue text = predicted Snf1-related (SRK) ortholog, and orange text = predicted AMPK-related (ARK) ortholog.
(PDF)

S2 Table Sub-classification of the human and yeast families of kinases related to AMPK and Snf1, respectively. (PDF)

S3 Table Strains generated and/or used in this study including: strain designation, background, genotype, and source. (PDF)

S4 Table Plasmids generated and/or used in this study including: plasmid designation, backbone, genotype, and source. (PDF)

S1 Data Raw data and statistical reporting for Fig 3. (XLSX)

S2 Data Raw data and statistical reporting for Fig 4. (XLSX)

S3 Data Raw data and statistical reporting for Fig 5. (XLSX)

S4 Data Raw data and statistical reporting for Fig 6. (XLSX)

S5 Data Raw data and statistical reporting for Fig 7. (XLSX)

S6 Data Raw data and statistical reporting for Fig 8. (XLSX)

S7 Data Raw data and statistical reporting for Fig 9. (XLSX)

S8 Data Raw data and statistical reporting for Fig 10. (XLSX)

S9 Data Raw data and statistical reporting for S4 Fig. (XLSX)

S10 Data Raw data and statistical reporting For S5 Fig. (XLSX)

S11 Data Raw data and statistical reporting For S7 Fig. (XLSX)

S12 Data Raw data and statistical reporting for S8 Fig. (XLSX)

S13 Data Raw data and statistical reporting for S9 Fig. (XLSX)

S14 Data Raw data and statistical reporting for S14 Fig. (XLSX)

S15 Data Raw data and statistical reporting for S17 Fig. (XLSX)

S16 Data Raw data and statistical reporting for S18 Fig. (XLSX)

S17 Data Raw data and statistical reporting for S19 Fig. (XLSX)
