# Supplementary figures and images for: A Snf1-related nutrient-responsive kinase antagonizes endocytosis in yeast
Source: PLoS Genet. 2020 Mar 19;16(3):e1008677. doi: 10.1371/journal.pgen.1008677 (PMC7176151; doi:10.1371/journal.pgen.1008677)

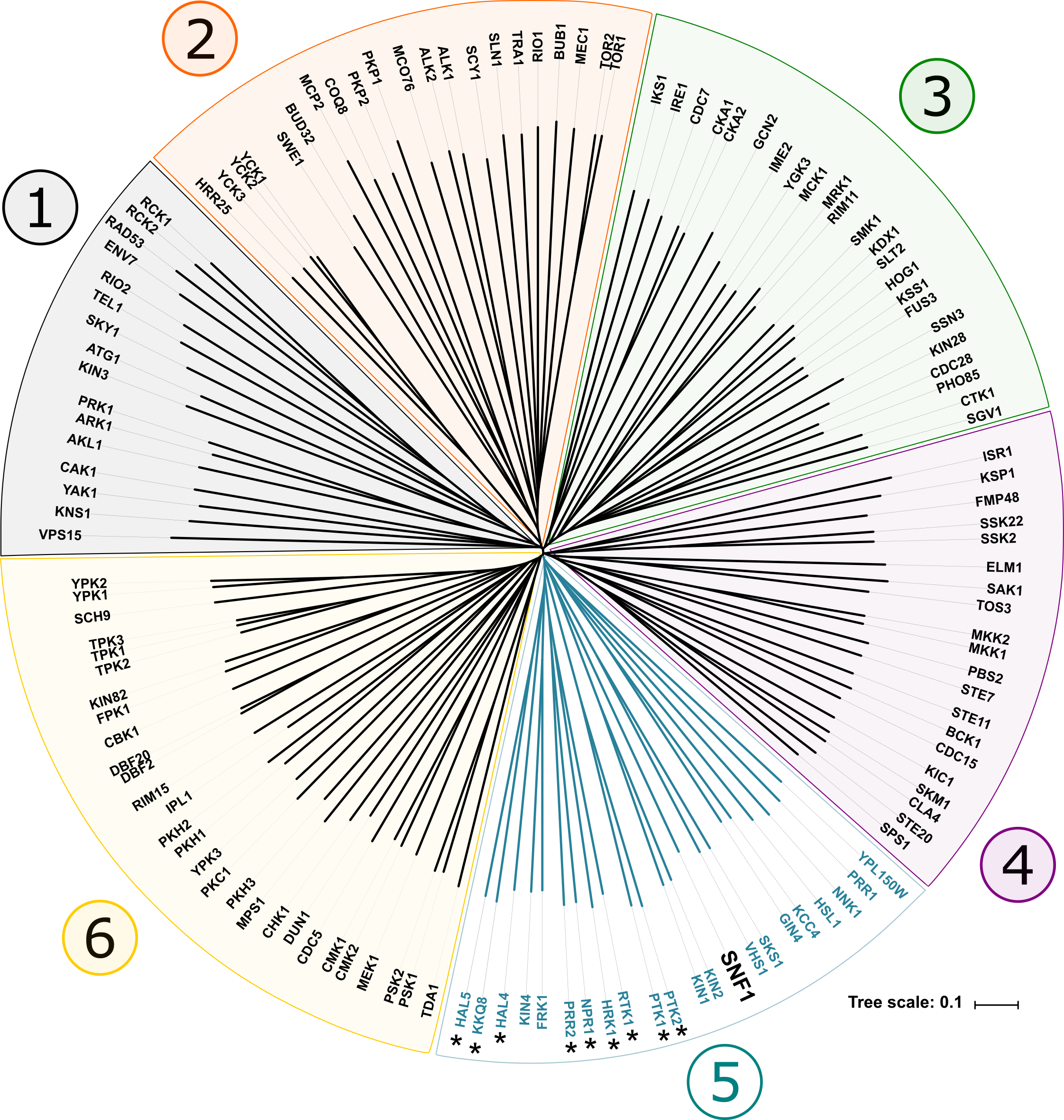

Supplement: S1 Fig — The protein kinases cluster into 6 major clades, which have been arbitrarily numbered and color-coded for simplicity and ease of viewing across different figures. The 5th clade in teal contains Snf1. Kinases clustering with Snf1 include many kinases originally described as the NPR/HAL5 family, denoted by black asterisks (PTK1, PTK2, NPR1, PRR2, RTK1, HRK1, HAL5, KKQ8, and HAL4) GCN2 and CHK1, also originally described as NPR/HAL5 family members, are clustering with groups 3 and 6, respectively. (TIF) [file pgen.1008677.s001.tif]

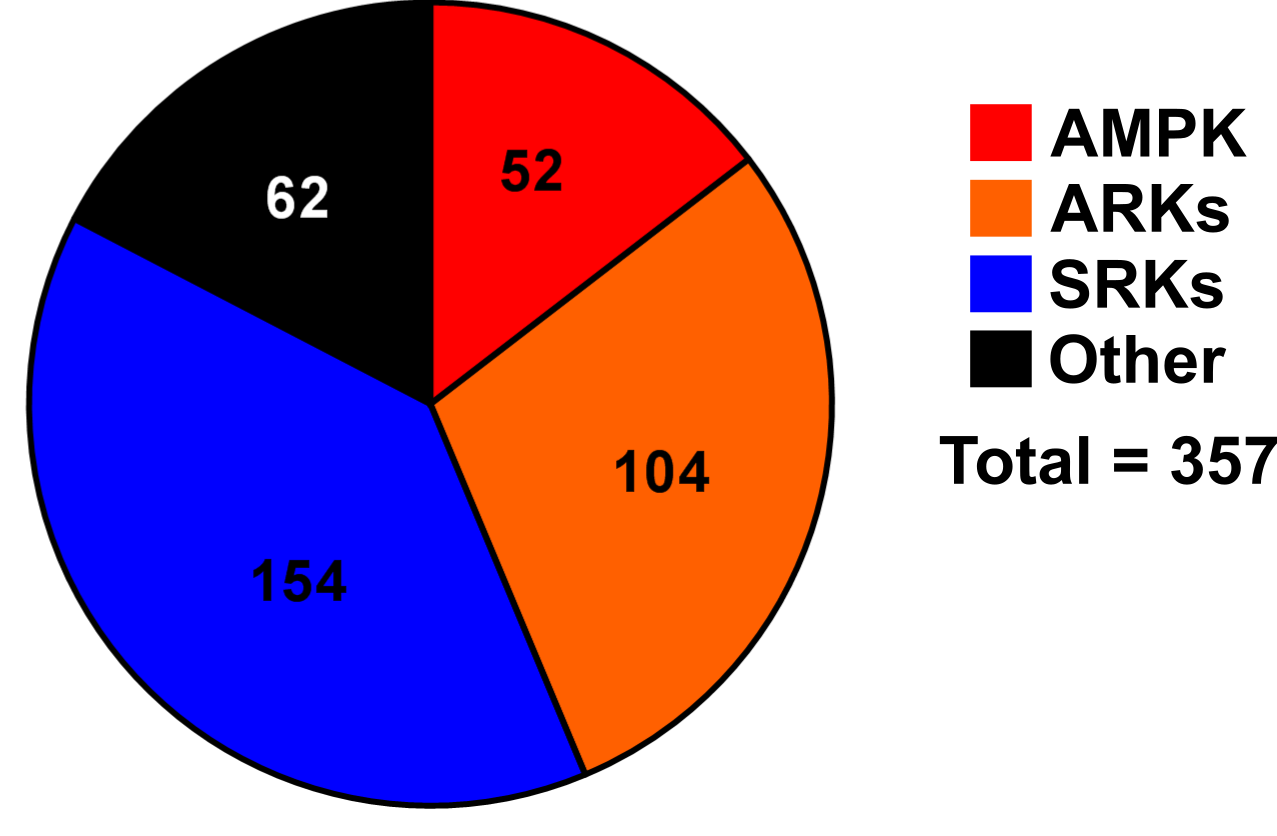

Supplement: S2 Fig — Every predicted ortholog for kinases clustering with Snf1 by multiple sequence alignment was tallied and identified as AMPK (red), AMPK-related (ARKs, orange), Snf1-related (SRKs, blue), or Other (black). (TIF) [file pgen.1008677.s002.tif]

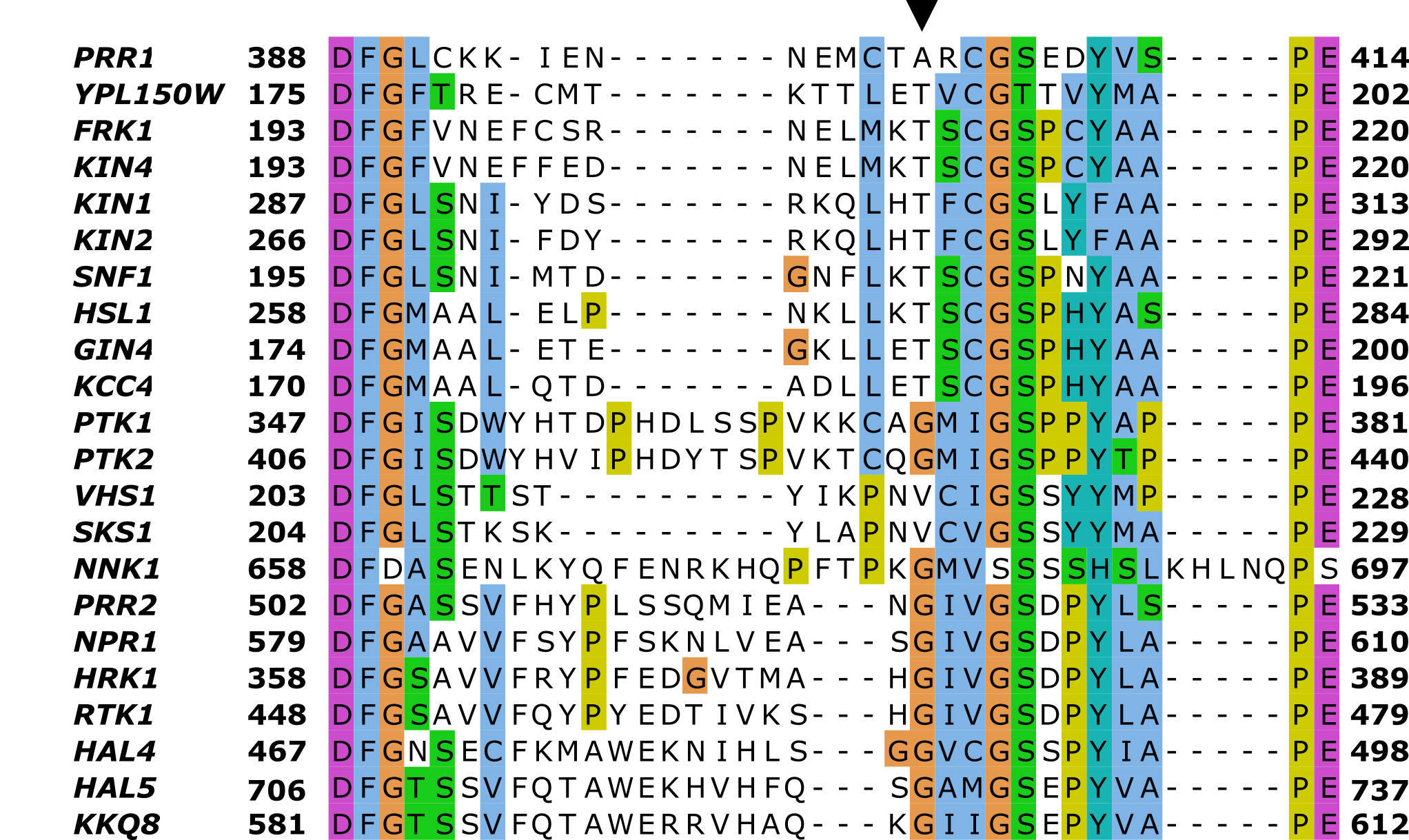

Supplement: S3 Fig — The amino acid position aligning with T210, critical threonine of the Snf1 activation loop [108], is denoted by the black indicator. (TIF) [file pgen.1008677.s003.tif]

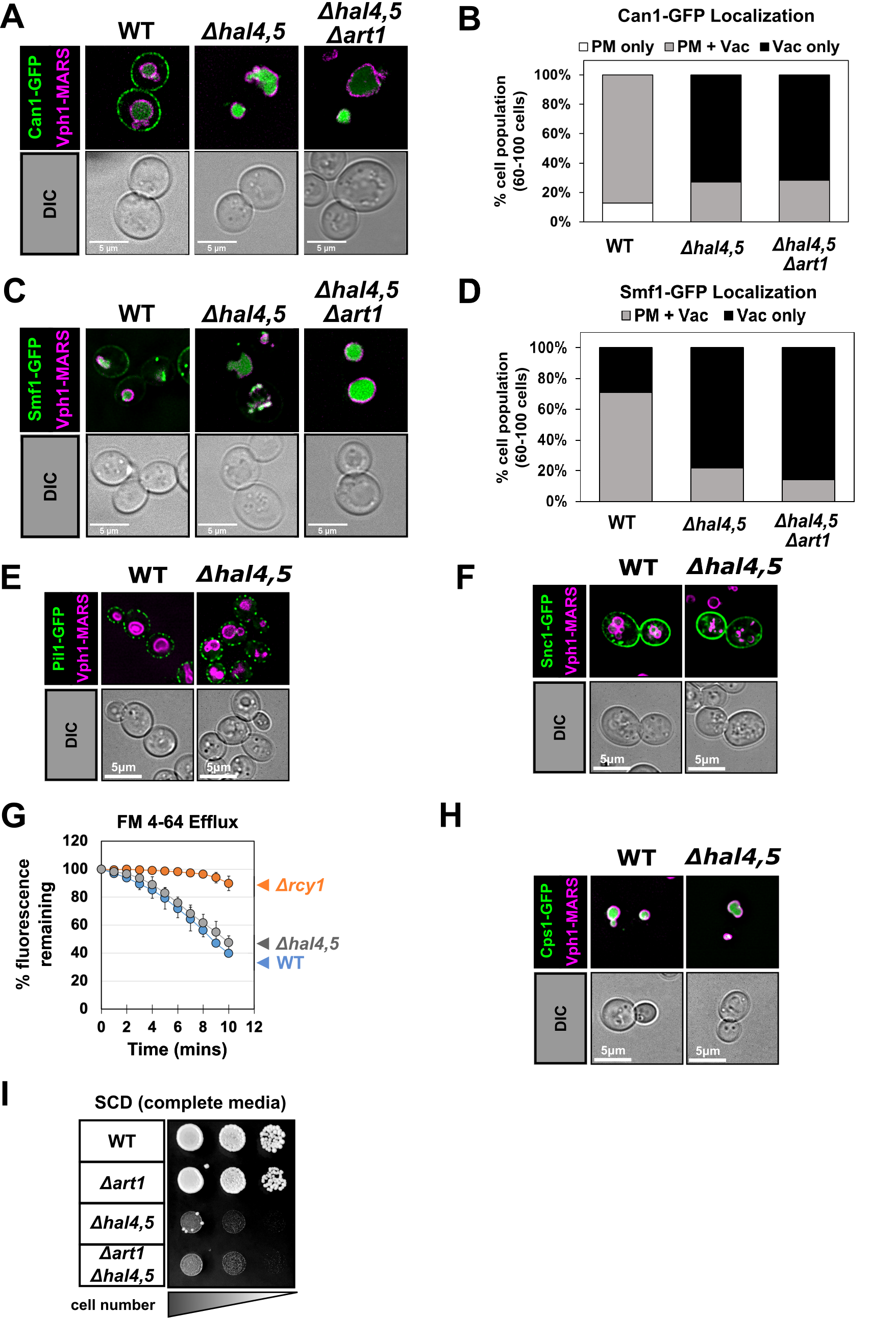

Supplement: S4 Fig — (A) Representative images of Can11-GFP expressed from a centromeric plasmid under native promoter control in the presence of endogenously MARS tagged Vph1, a marker for the limiting membrane of the vacuole. WT, Δhal4Δhal5 cells, or Δhal4Δhal5Δart1 cells were cultured to mid-log phase in selective media. (B) Quantification of Can1-GFP localization in (A) performed by binning cells into localization categories as indicated. (C) Representative images of Smf1-GFP expressed from a centromeric plasmid under native promoter control in the presence of endogenously MARS tagged Vph1, a marker for the limiting membrane of the vacuole. WT, Δhal4Δhal5 cells, or Δhal4Δhal5Δart1 cells were cultured to mid-log phase in selective media. (D) Quantification of Smf1-GFP localization in (C) performed by binning cells into localization categories as indicated. (E) Representative images of Pil1-GFP expressed from a centromeric plasmid under native promoter control in the presence of endogenously-tagged Vph1-MARS, a marker for the limiting membrane of the vacuole. WT and Δhal4,5 mutant cells were imaged after being cultured to mid-log phase in selective media. (F) Representative images of Snc1-GFP expressed from a centromeric plasmid under native promoter control in WT and Δhal4,5 cells in the presence of endogenously MARS tagged Vph1, a marker for the limiting membrane of the vacuole. (G) Percentage of cell population positive for FM 4–64 fluorescence as measured by cells that fall within a defined PE gate (red fluorescence) as measured by flow cytometry (10,000 cells counted per condition, n = 3 biological replicates) in WT, Δhal4,5, or Δrcy1 cell populations (grown to mid-log phase in rich media). This assay is an indirect measure of endosomal lipid recycling by monitoring loss of membrane-bound FM 4–64 due to efflux into the media over time. (H) Representative images of Cps1-GFP under conditions previously described in (C). (I) Representative image of cells serially diluted on syntheti [file pgen.1008677.s004.tif]

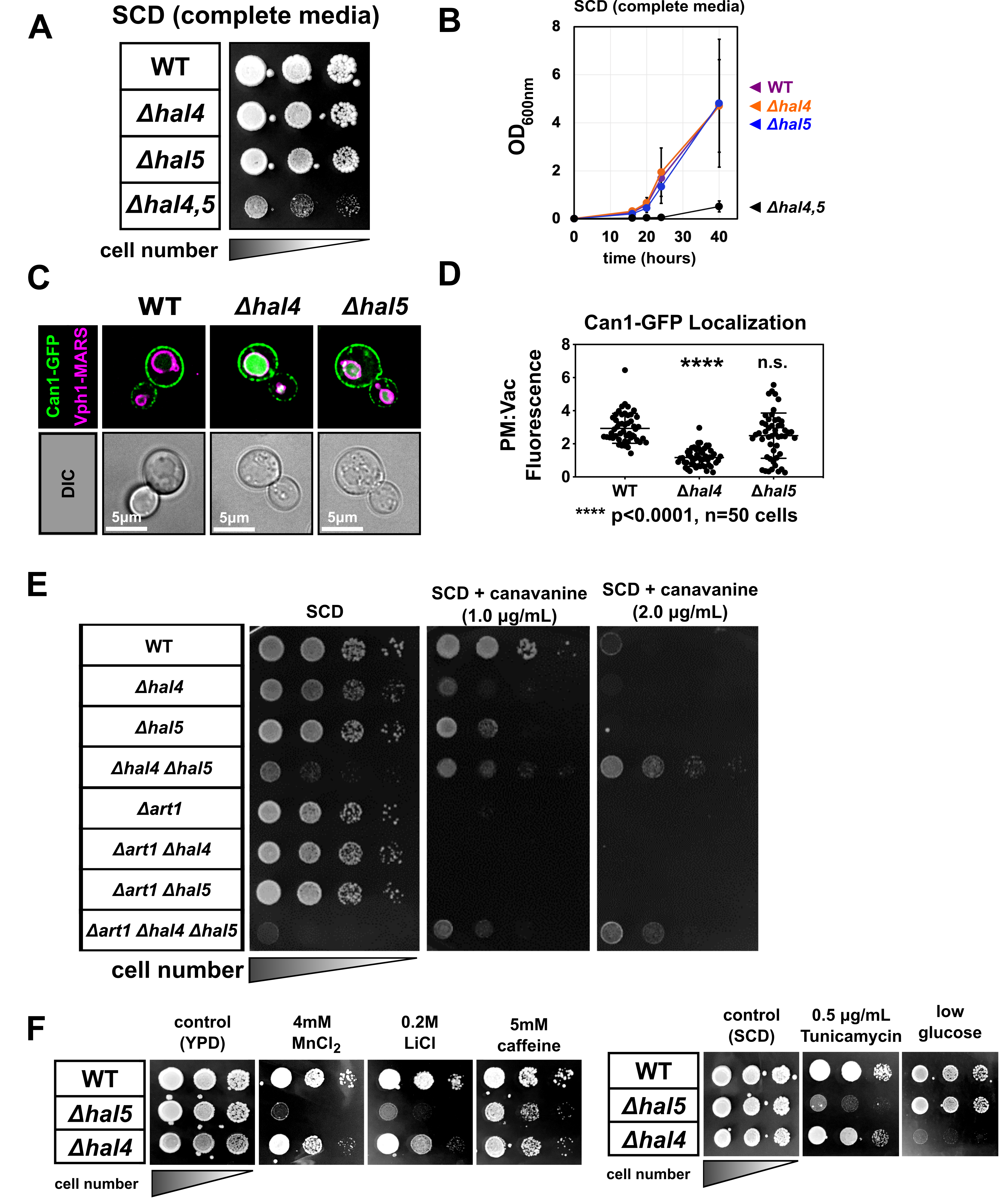

Supplement: S5 Fig — (A) Representative image of cells serially diluted on synthetic complete media and grown for 3 days to assess growth of various hal mutants (B) Growth of cells seeded at 0.05 OD from mid-log phase and monitored over time for OD600nm in synthetic complete liquid media. (C) Representative images of Can11-GFP expressed from a centromeric plasmid under native promoter control in the presence of endogenously MARS tagged Vph1, a marker for the limiting membrane of the vacuole. WT and Δhal4 or Δhal5 single mutant cells were cultured to mid-log phase in selective media. (D) Quantification of Can1-GFP localization in (C) was performed by measuring the ratio of GFP signal at the PM compared to the vacuole (PM:VAC). Double mutants are excluded from this analysis due to lack of signal at the PM. (E) Representative image of indicated cells serially diluted on synthetic complete media to assess sensitivity (or resistance) to growth in the presence of the indicated concentration of canavanine, a toxic arginine analog. (F) Representative image of cells serially diluted onto indicated media and grown for 3 (YPD) or 5 (SCD) days to assess growth of Δhal4 and Δhal5 single mutants under Tunicamycin, an ER protein folding stress, low glucose (0.2% glucose compared to 2% in control), manganese, lithium, or caffeine stresses. (TIF) [file pgen.1008677.s005.tif]

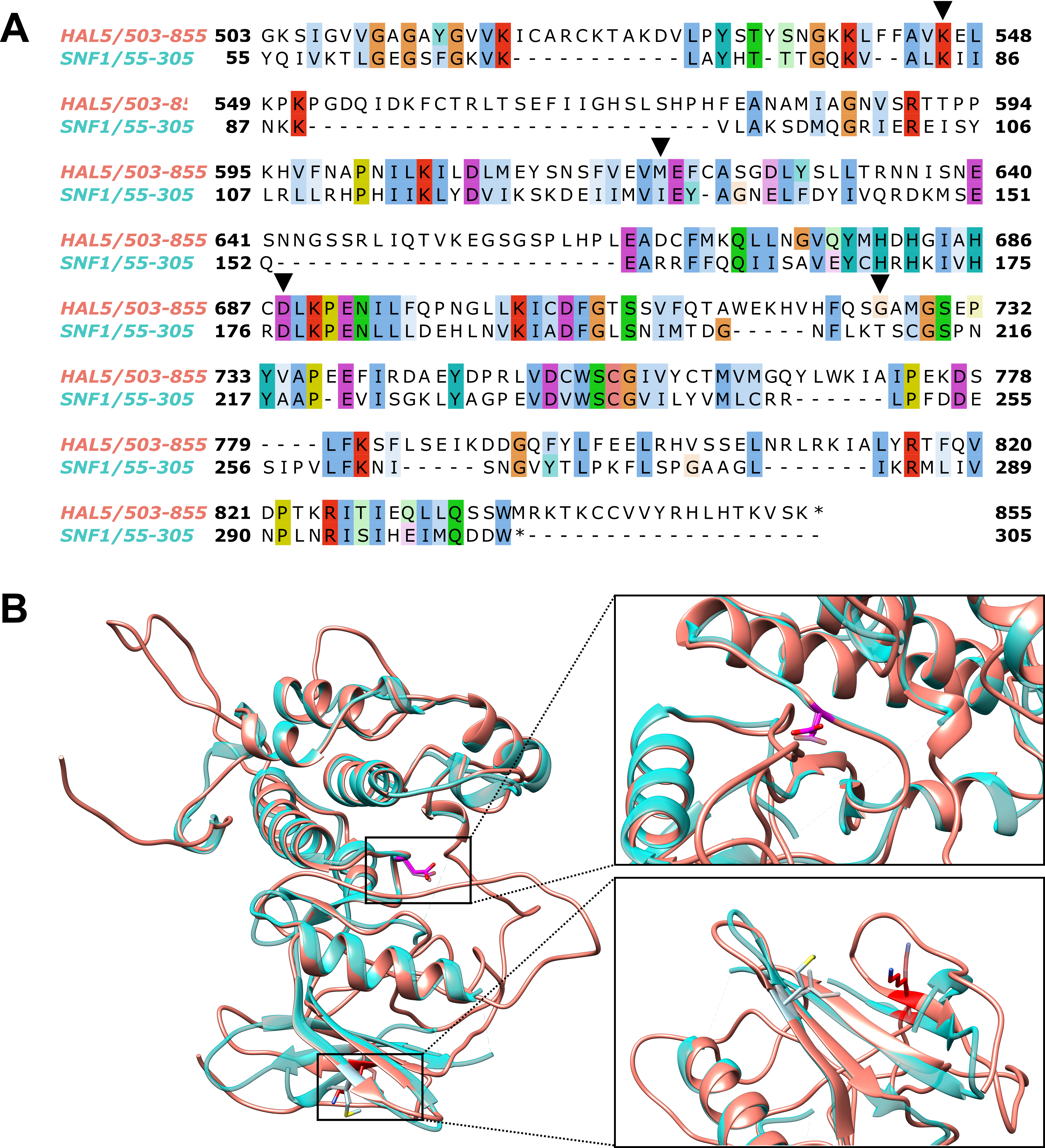

Supplement: S6 Fig — (A) A pairwise sequence alignment, performed using EMBOSS (EMBL-EBI) and visualized using JalView, of the Hal5 and Snf1 catalytic domains to identify important conserved residues of Hal5 (including K546, M620, and D688 as well as lack of a conserved threonine in the activation loop at Snf1 T210) (B) The pairwise alignment of Snf1 and Hal5 catalytic domains was then used to model Hal5 (pink) onto Snf1(cyan) structure using MODELLER through the Chimera interface. In the panel at the top-right is a zoomed-in view of the conserved catalytic aspartate residues in the active sites. In the panel at the bottom-right is a zoomed-in view of the conserved ATP-coordinating lysine residues (in red) and the gatekeeper residues (in light blue) in the ATP-binding pockets. (TIF) [file pgen.1008677.s006.tif]

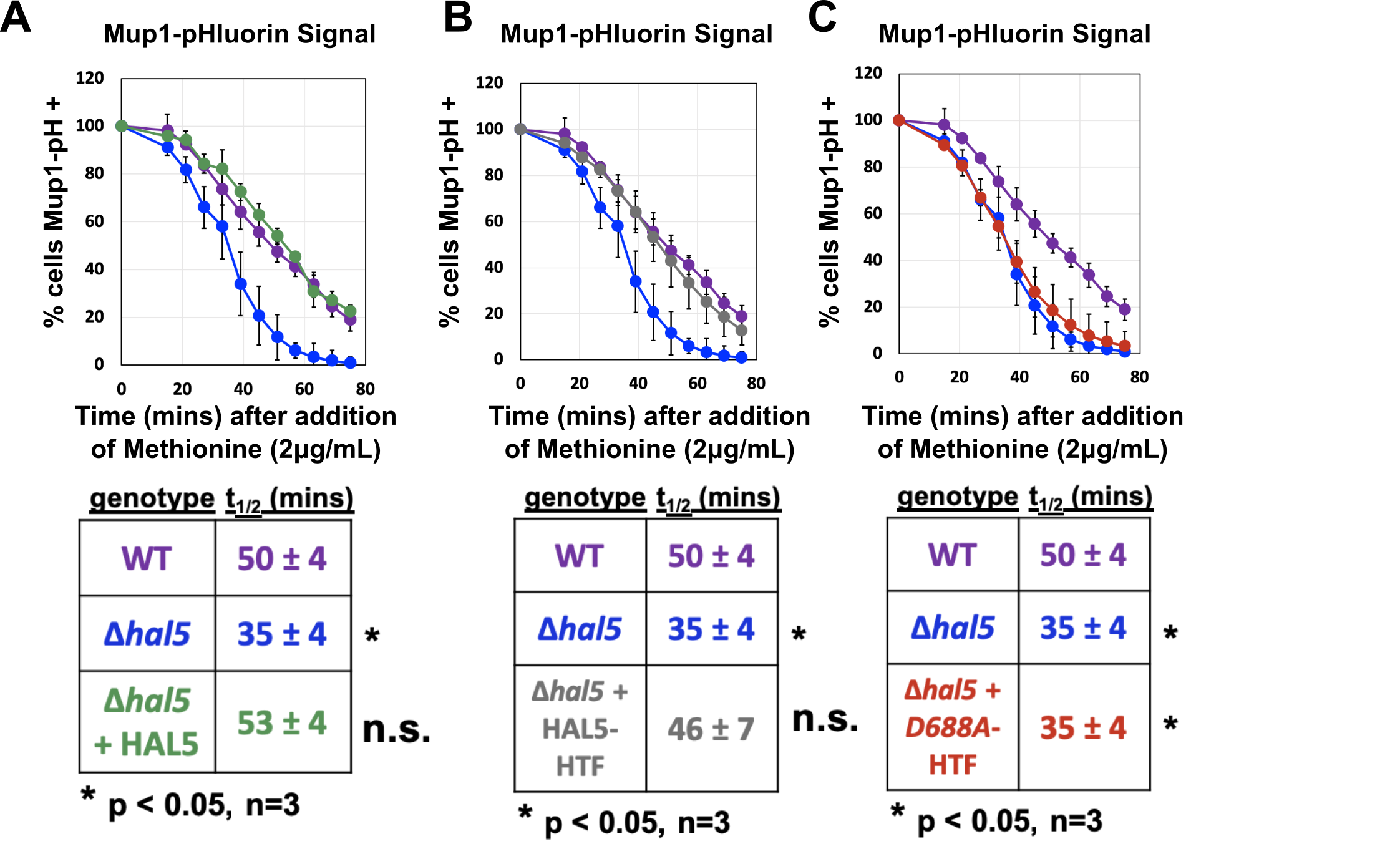

Supplement: S7 Fig — Δhal5 mutant cells expressing endogenously-tagged Mup1-pHluorin and exogenously expressed (A) native Hal5 (HAL5), (B) C-terminally-tagged Hal5 (HAL5-HTF) or (C) C-terminally-tagged catalytic dead Hal5 (D688A-HTF). Percentage of cell population expressing endogenously tagged Mup1-pHluorin as measured by cells that fall within a defined FITC gate (green fluorescence) by flow cytometry (10,000 cells counted per condition, n = 3 biological replicates) over time in response to methionine, an endocytic stimulant. Mup1-pH PM half-time (t1/2) estimated based on initial and final time points and elapsed time. (TIF) [file pgen.1008677.s007.tif]

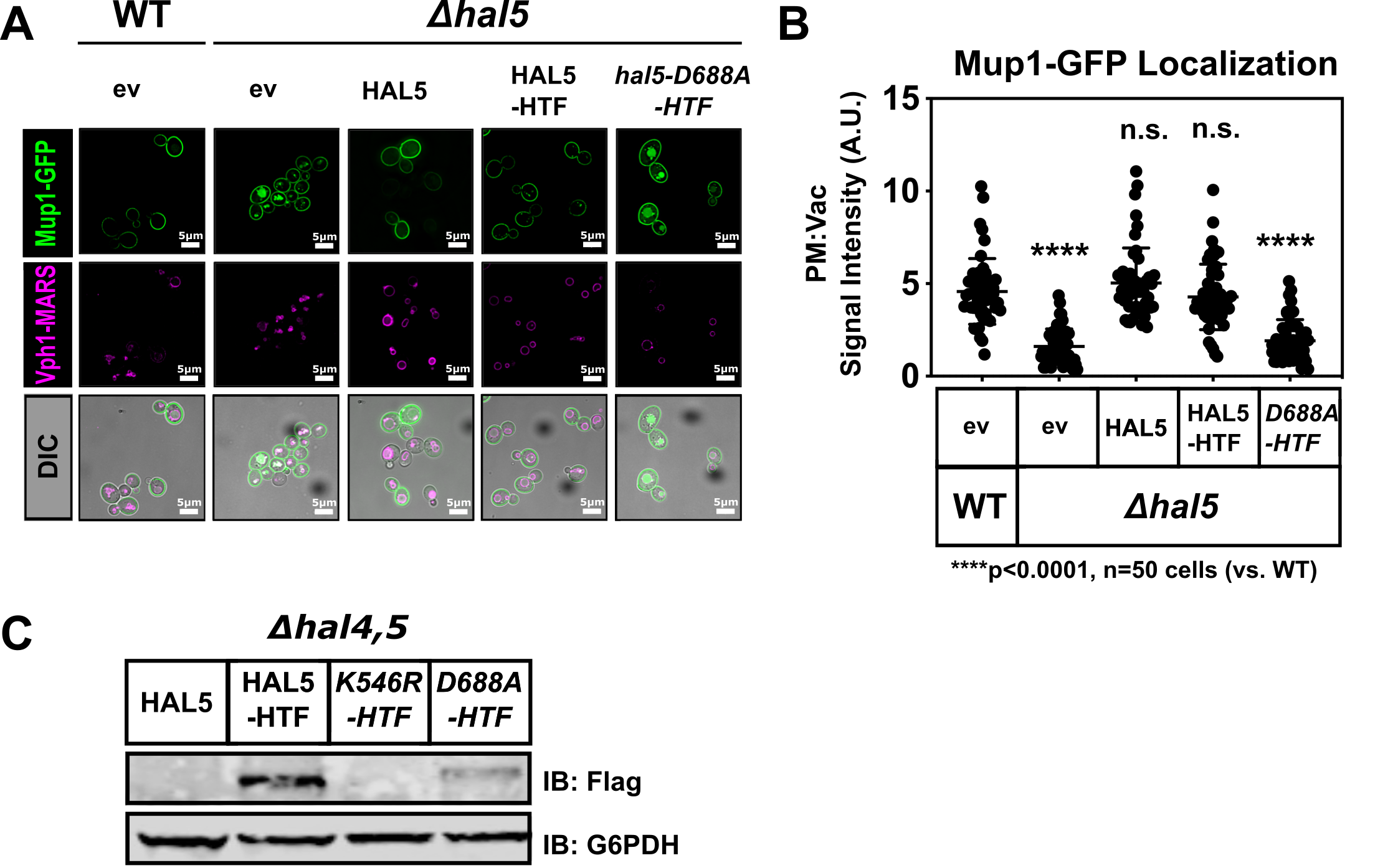

Supplement: S8 Fig — (A) Representative images of Mup1-GFP expressed from a centromeric plasmid in the presence of endogenously MARS tagged Vph1, a marker for the limiting membrane of the vacuole. HAL5 is exogenously expressed in the absence of endogenous Hal5 from a centromeric plasmid under native promoter control with either no tag (HAL5), a C-terminal 6xHIS-TEV-3xFLAG tag (HAL5-HTF), or a C-terminally-tagged catalytic dead variant (D688A-HTF). (B) Quantification of (A) by measuring the ratio of Mup1-GFP signal at the PM compared to the vacuole (PM:VAC). (C) Immunoblot analysis of C-terminally-tagged Hal5 variants described in (A) as well as an additional C-terminally-tagged catalytic dead variant (hal5-K546R-HTF). (TIF) [file pgen.1008677.s008.tif]

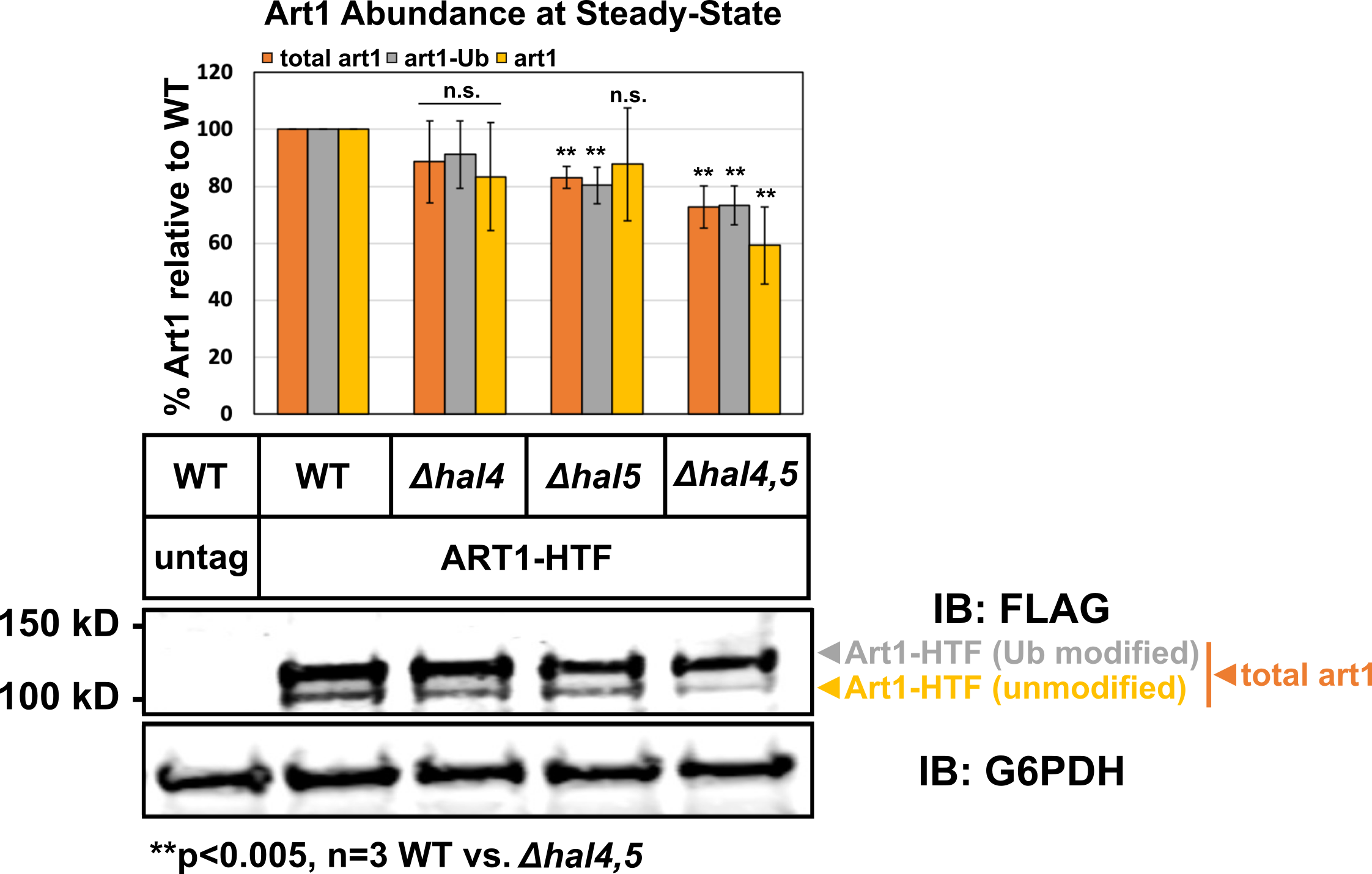

Supplement: S9 Fig — Total art1 (orange bars) was quantified by measuring signal of both bands corresponding to Art1, and normalizing to G6PDH levels. Unmodified Art1 (yellow bars) was quantified by measuring signal of the bottom band, and normalizing to G6PDH levels. Ubiquitin-modified (Ub modified) Art1 (gray bars) was quantified by measuring signal of the top band, and normalizing to G6PDH levels. N = 3 (TIF) [file pgen.1008677.s009.tif]

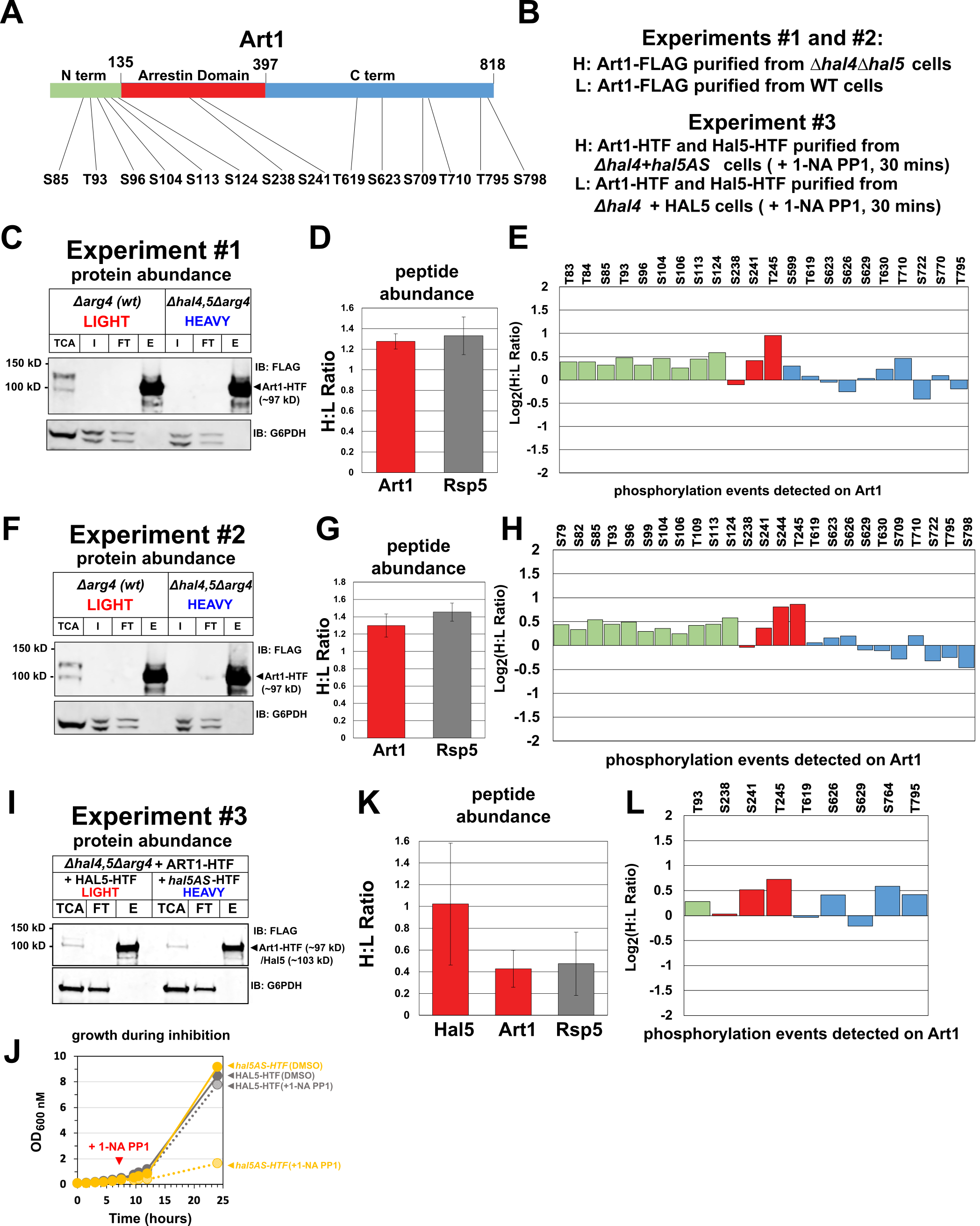

Supplement: S10 Fig — (A) Schematic of the domain architecture of the Art1 protein, with known phosphorylation sites indicated. (B) Description of SILAC-MS experiments performed to profile Art1 phosphorylation with and without Hal4 and Hal5 kinase activity. (C-L) Analysis of experiments described in (B). (C,F,I) 2% of prepared samples were analyzed by immunoblot to confirm Art1-HTF bait purification. Samples were subsequently submitted for mass spectrometry analysis. (D,G,K) The H:L ratio for all peptides of the indicated proteins were averaged to compute a measurement of the H:L ratio for the protein in the indicated experiment. Additional cell material was collected from hal mutant cells to compensate for the observed loss in Art1 abundance (S5 Fig) (i.e. 1L of 0.5 OD WT cells vs 0.75 OD hal cells). (E,H,L) The LOG2(H:L ratio) (normalized to total Art1) for each phosphorylation event detected is plotted and color-coded to correspond to the region of the Art1 protein as indicated in (A). (J) For experiment #3, following treatment of cultures with 1-NA-PP1 (and just prior to sample collection) 10mL of each culture was removed from the sample and cultured for an additional 24 hours in order to confirm inhibition by the compound. (TIF) [file pgen.1008677.s010.tif]

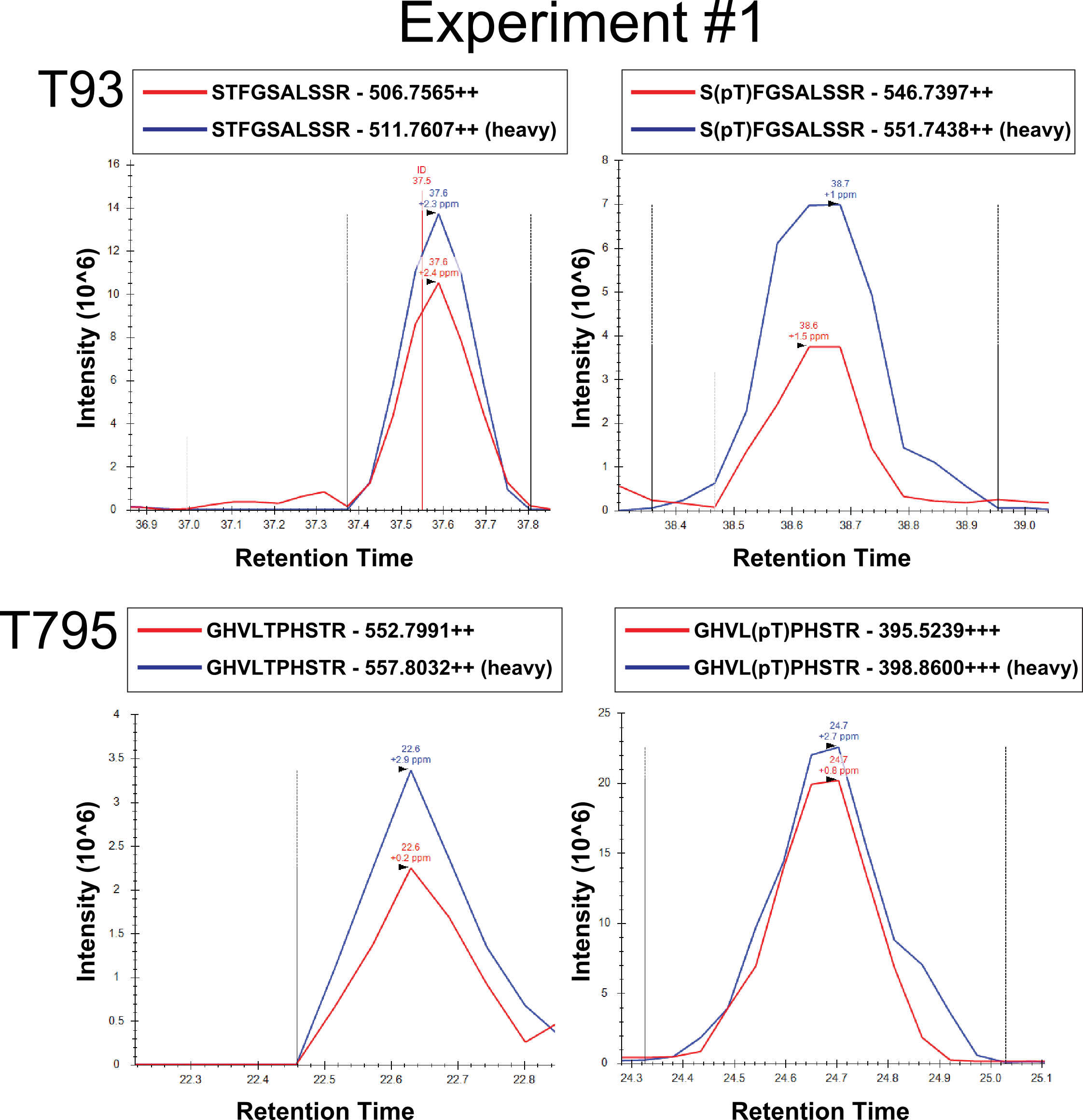

Supplement: S11 Fig — Filtered chromatography data is shown for the indicated peptides (light peptide in red and heavy peptide in blue). Phosphopeptides identifying phosphorylation at Thr93 (top right) and Thr795 (bottom right) and the corresponding unmodified peptides (top left and bottom left, respectively) are depicted. (TIF) [file pgen.1008677.s011.tif]

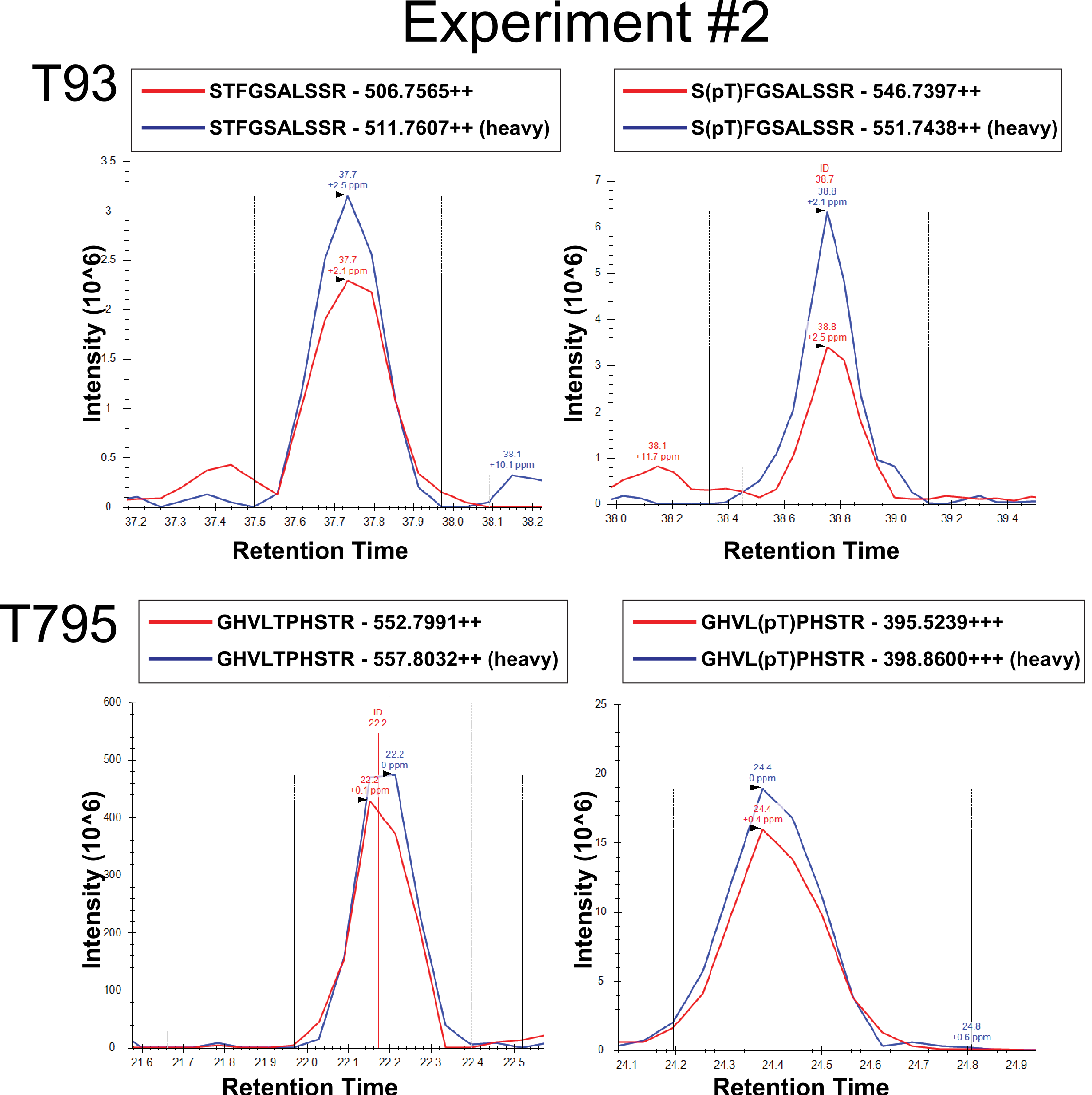

Supplement: S12 Fig — Filtered chromatography data is shown for the indicated peptides (light peptide in red and heavy peptide in blue). Phosphopeptides identifying phosphorylation at Thr93 (top right) and Thr795 (bottom right) and the corresponding unmodified peptides (top left and bottom left, respectively) are depicted. (TIF) [file pgen.1008677.s012.tif]

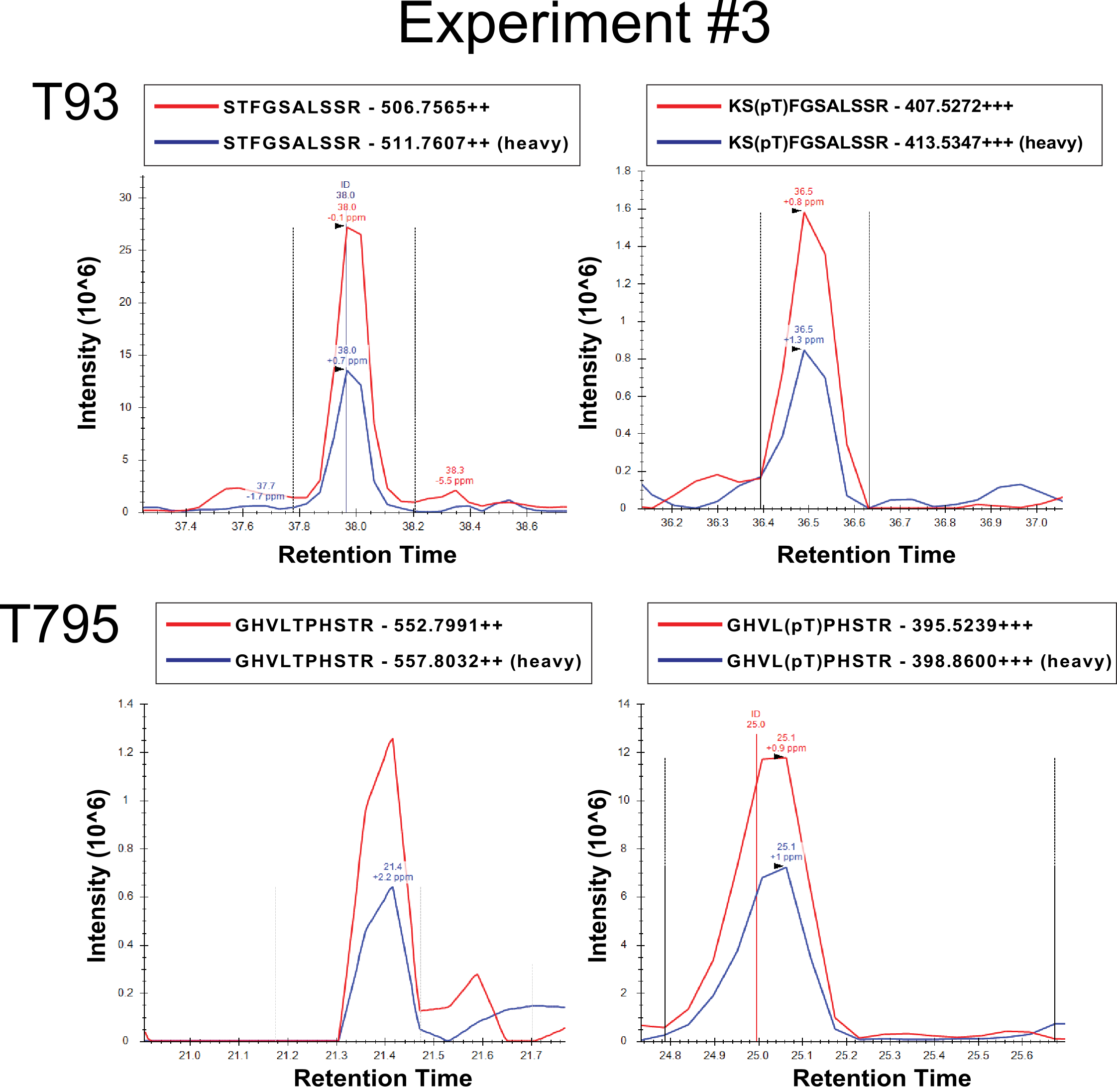

Supplement: S13 Fig — Filtered chromatography data is shown for the indicated peptides (light peptide in red and heavy peptide in blue). Phosphopeptides identifying phosphorylation at Thr93 (top right) and Thr795 (bottom right) and the corresponding unmodified peptides (top left and bottom left, respectively) are depicted. (TIF) [file pgen.1008677.s013.tif]

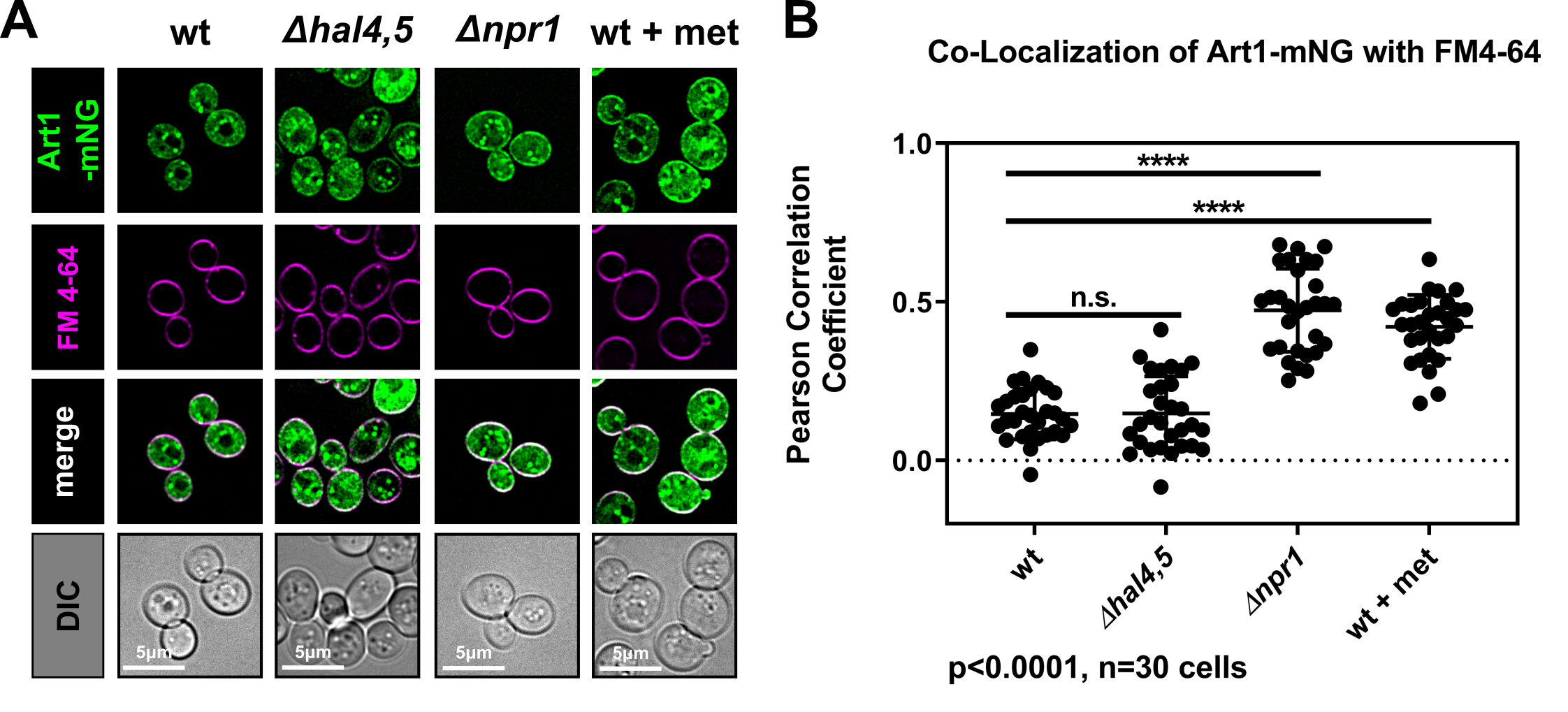

Supplement: S14 Fig — In the far right column of the panel, WT cells were treated with methionine (2μg/mL) for 10 minutes prior to FM 4–64 pulse. (B) Art1 localization to the PM in (A) was quantified by measuring Pearson’s correlation coefficient of Hal5-mNG signal with FM 4–64 signal. (TIF) [file pgen.1008677.s014.tif]

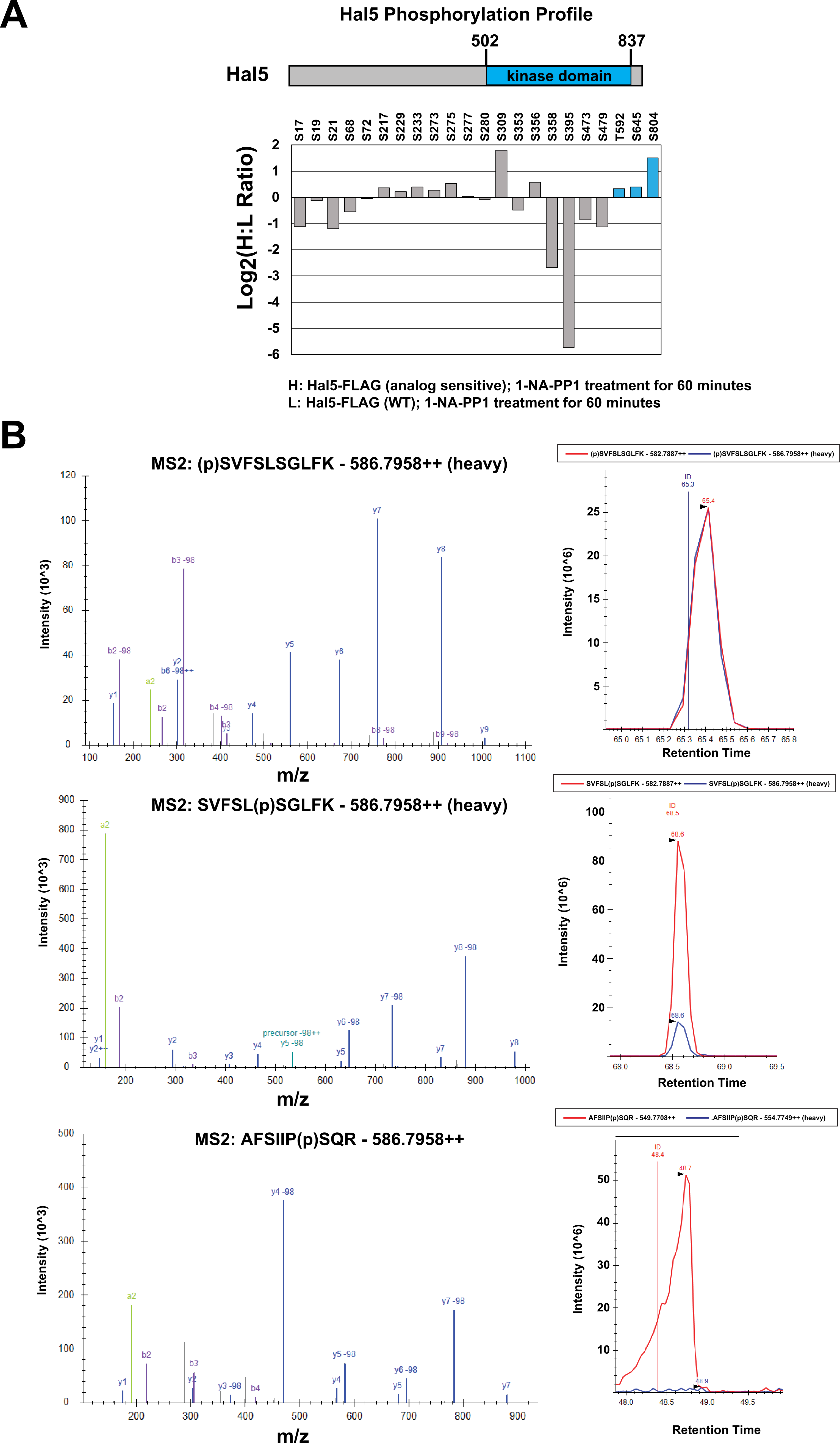

Supplement: S15 Fig — (A) Quantitative phosphoprofiling analysis Hal5 based on SILAC-MS data. Schematic of the domain architecture of Hal5 is shown at the top. (B) Fingerprinting and quantification of individual phosphorylation events resolved for Hal5. MS2 spectra for individual phosphopeptides (left panels) and filtered chromatograms for quantification of light (red) and heavy (blue) peptides (right panels) are shown for Ser353 (top), Ser358 (middle) and Ser395 (bottom). (TIF) [file pgen.1008677.s015.tif]

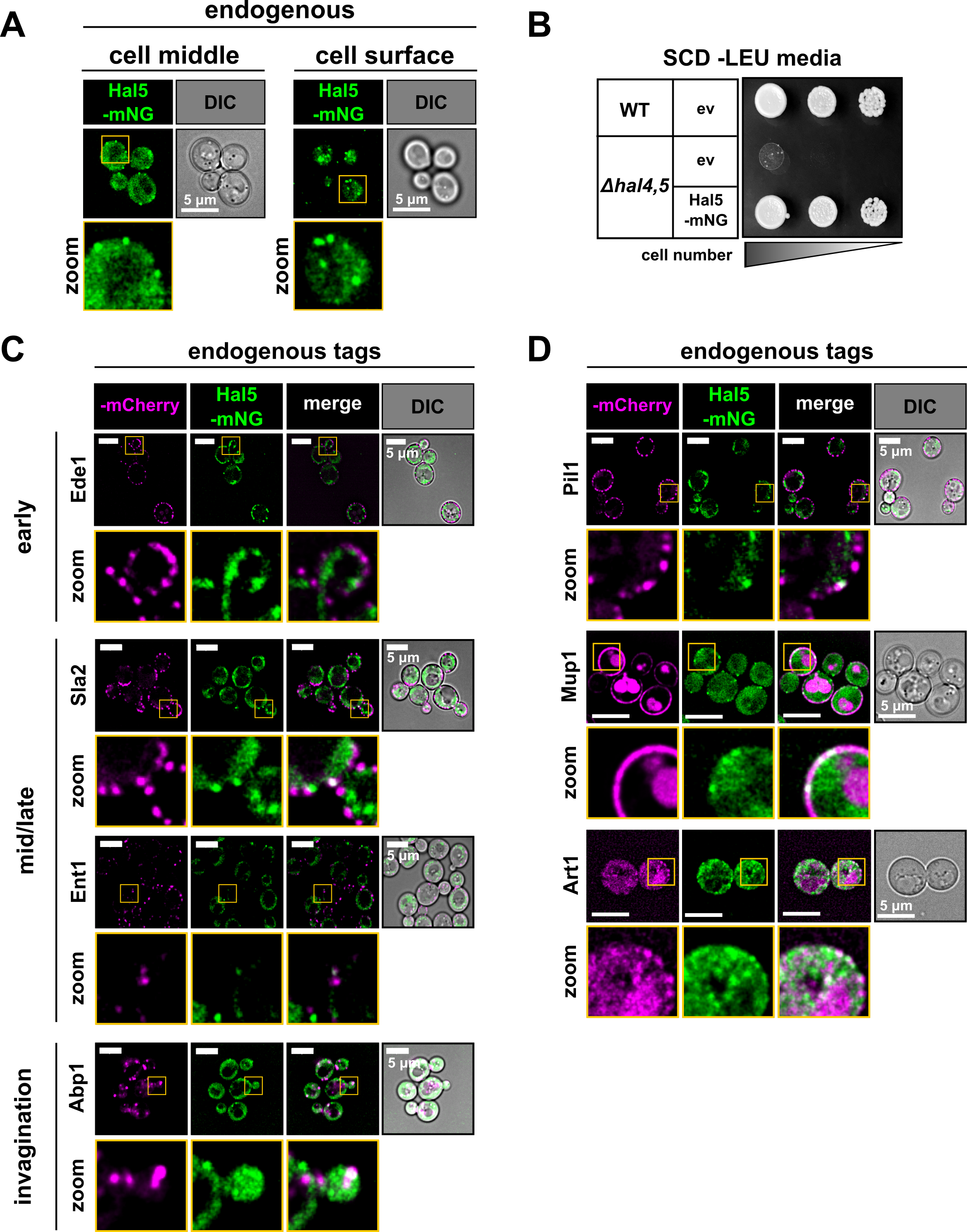

Supplement: S16 Fig — (A) Representative images of cells expressing endogenously-tagged Hal5-mNG grown to mid-log phase in rich media imaged near the cell middle (left) or cell surface (right) to asses Hal5 localization. (B) Cells expressing either empty vector (ev) or Hal5-mNG serially diluted onto synthetic selective media and grown for 5 days to assess functionality of C-terminally-tagged Hal5-mNG. (C) Cells co-expressing endogenous Hal5-mNG and mCherry-tagged components of endocytic site machinery corresponding to either early (top), mid/late (middle), or invagination (bottom) events. (D) Cells co-expressing endogenous Hal5-mNG and mCherry-tagged Pil1 (eisosomes), Mup1 (nutrient transporter, broad PM marker), or Art1 (Rsp5 adaptor). (TIF) [file pgen.1008677.s016.tif]

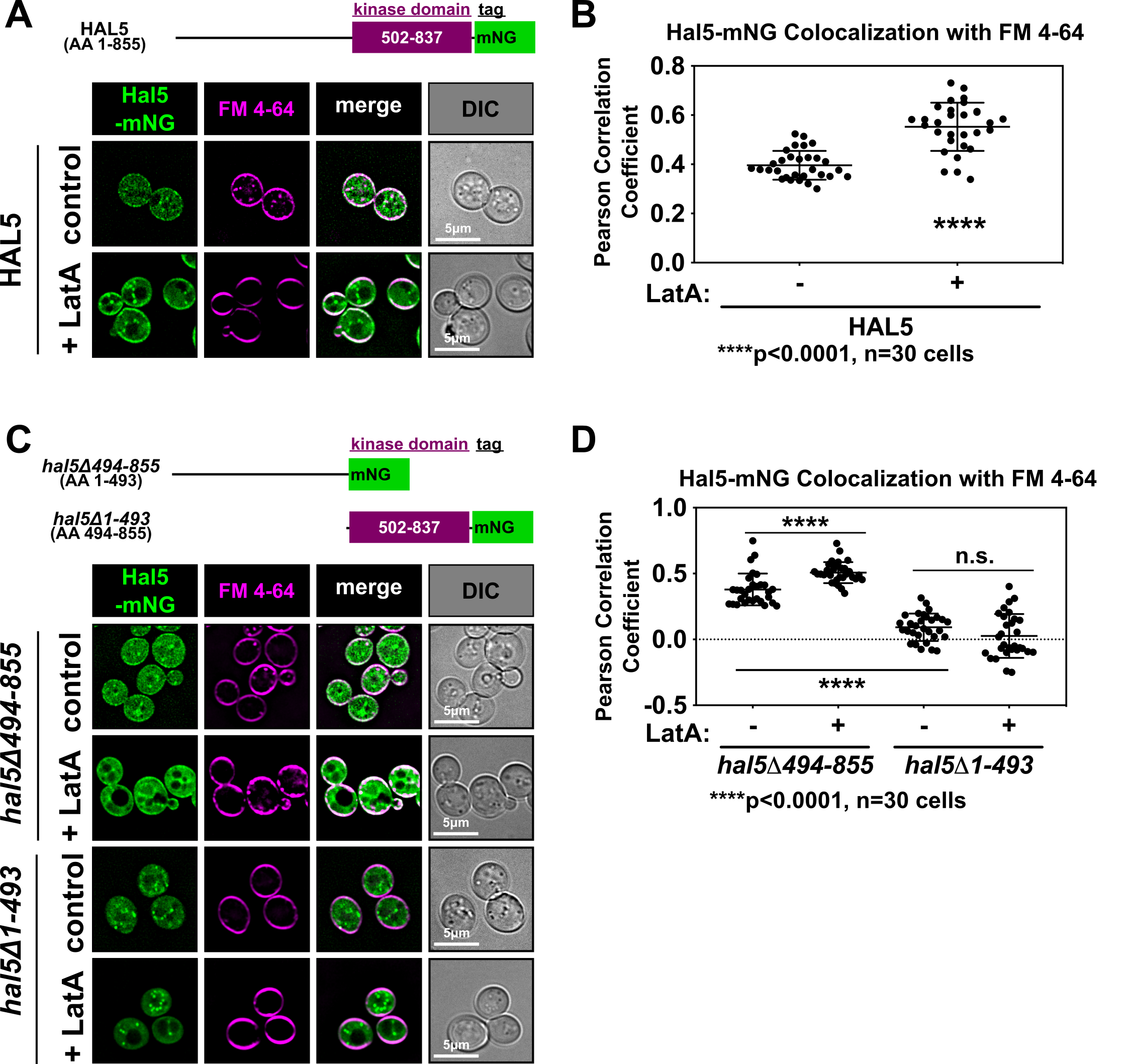

Supplement: S17 Fig — (A) Representative images of WT cells grown to mid-log phase in selective media expressing Hal5-mNG (with or without 1 hour LatA treatment) after brief FM 4–64 pulse to label PM immediately prior to imaging (B) Localization of Hal5 to the PM was quantified in (A) by measuring Pearson correlation coefficient of Hal5-mNG signal with FM 4–64 signal. (C) Representative images using conditions described in (A) for WT cells expressing either a Hal5 variant deleted for the N-terminal region (hal5Δ1-493-mNG) or a Hal5 variant deleted for the kinase domain (hal5Δ494-855-mNG). (D) Quantification of Hal5 localization to the PM in (C) performed as described in (B). (TIF) [file pgen.1008677.s017.tif]

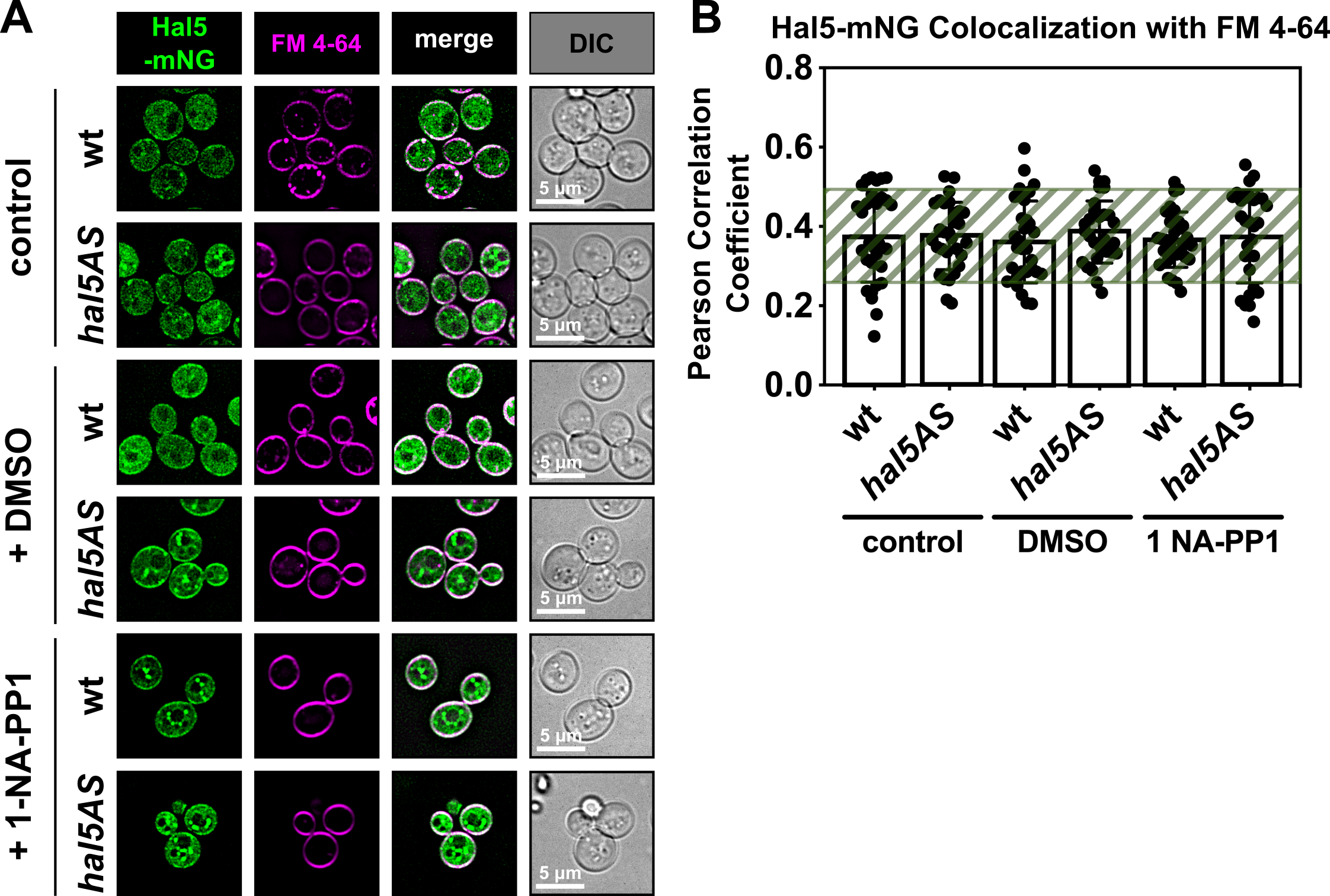

Supplement: S18 Fig — (A) Representative images of WT cells expressing WT or analog-sensitive variants of Hal5-mNG (wt or hal5AS) from a centromeric plasmid under native promoter control. Cells were grown to mid-log phase in selective media. Cells were untreated (control), treated with vehicle (DMSO), or inhibitor (1-NA-PP1 26.3μM) for 10 minutes, then briefly pulsed with FM 4–64 to label PM immediately prior to imaging. (B) Hal5 localization to the PM was quantified in (A) by measuring Pearson correlation coefficient of Hal5-mNG signal with FM 4–64 signal. Standard deviation of control WT cells denoted by green box. (TIF) [file pgen.1008677.s018.tif]

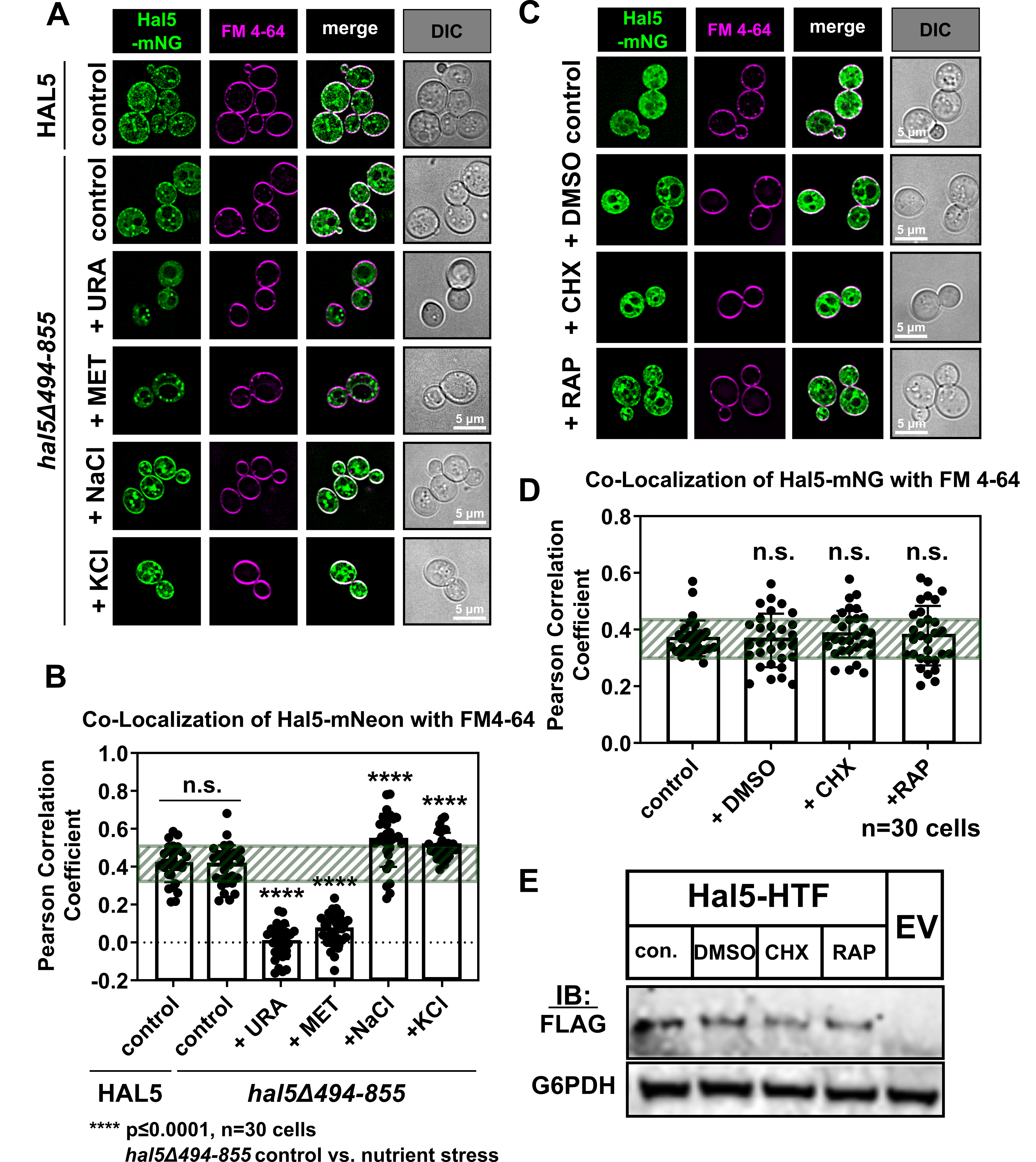

Supplement: S19 Fig — (A) Representative images of WT cells expressing a C-terminally mNG-tagged variant of Hal5 deleted for the kinase domain from a centromeric plasmid under native promoter control. Cells were grown to mid-log phase in selective media then switched to media with the indicated nutrient conditions (10 μg/mL methionine (+ MET), 10 μg/mL uracil (+URA)) for 10 minutes, then briefly pulsed with FM 4–64 to label PM immediately prior to imaging. (B) Hal5 localization to the PM was quantified in (A) by measuring Pearson correlation coefficient of Hal5-mNG signal with FM 4–64 signal. Standard deviation of WT cells is denoted by the green box. (C) Representative images of WT cells expressing full-length Hal5-mNG as described in (A). Prior to pulsing with FM 4–64 to label the PM, cells were treated with either DMSO (mock), 50 μg/mL of cycloheximide (+ CHX) or 200 ng/mL of rapamycin (+ RAP) for 15 minutes. (D) Quantification of (C) as described in (B). (E) Immunoblot analysis of whole cell lysates collected from WT cells expressing full-length c-terminally tagged Hal5-HTF, treated with either DMSO (mock), Cycloheximide (50 μg/mL), or Rapamycin (200 ng/mL) for 15 minutes. EV indicates empty vector. (TIF) [file pgen.1008677.s019.tif]
