# Supplementary material for: A Snf1-related nutrient-responsive kinase antagonizes endocytosis in yeast
Source: PLoS Genet. 2020 Mar 19;16(3):e1008677. doi: 10.1371/journal.pgen.1008677 (PMC7176151; doi:10.1371/journal.pgen.1008677)
Supplement: S1 Table — Red text = predicted AMPK ortholog, blue text = predicted Snf1-related (SRK) ortholog, and orange text = predicted AMPK-related (ARK) ortholog. (PDF) [file pgen.1008677.s020.pdf]

Saccharomyces cerevisiae (budding yeast)	*Caenorhabditis elegans* (worm)		*Drosophila melanogaster* (fly)		*Danio rerio* (zebrafish)		*Mus musculus* (mouse)		*Homo sapiens* (human)	
PRR2	**aak-2** (human PRKAA1, PRKAA2) (AMP-Activated Kinase) [WBGene00020142]	ortholog	**Dmel/AMPKα** (human PRKAA2, PRKAA1) [FBgn0023169]	ortholog	**hunk** (human HUNK) [ZDB-GENE-050309-240]	ortholog	**Prkaa2** (human PRKAA2) [MGI:1336173]	ortholog	**HUNK** [30811]	ortholog
			**Dmel/CG10177** (human STK33) [FBgn0039083]	ortholog	**prkaa2** (human PRKAA2) [ZDB-GENE-081120-5]	ortholog	**Hunk** (human HUNK) [MGI:1347352]	ortholog	**PRKAA2** [5563]	ortholog
NPR1	**aak-2** (human PRKAA1, PRKAA2) (AMP-Activated Kinase) [WBGene00020142]	ortholog	**Dmel/AMPKα** (human PRKAA2, PRKAA1) [FBgn0023169]	ortholog	**hunk** (human HUNK) [ZDB-GENE-050309-240]	ortholog	**Prkaa2** (human PRKAA2) [MGI:1336173]	ortholog	**HUNK** [30811]	ortholog
			**Dmel/CG10177** (human STK33) [FBgn0039083]	ortholog	**prkaa2** (human PRKAA2) [ZDB-GENE-081120-5]	ortholog	**Hunk** (human HUNK) [MGI:1347352]	ortholog		
HRK1	**aak-2** (human PRKAA1, PRKAA2) (AMP-Activated Kinase) [WBGene00020142]	ortholog	**Dmel/AMPKα** (human PRKAA2, PRKAA1) [FBgn0023169]	ortholog	**hunk** (human HUNK) [ZDB-GENE-050309-240]	ortholog	**Prkaa2** (human PRKAA2) [MGI:1336173]	ortholog	none	
			**Dmel/CG10177** (human STK33) [FBgn0039083]	ortholog	**prkaa2** (human PRKAA2) [ZDB-GENE-081120-5]	ortholog	**Hunk** (human HUNK) [MGI:1347352]	ortholog		
RTK1	**aak-2** (human PRKAA1, PRKAA2) (AMP-Activated Kinase) [WBGene00020142]	ortholog	**Dmel/AMPKα** (human PRKAA2, PRKAA1) [FBgn0023169]	ortholog	**hunk** (human HUNK) [ZDB-GENE-050309-240]	ortholog	**Prkaa2** (human PRKAA2) [MGI:1336173]	ortholog	**HUNK** [30811]	ortholog
			**Dmel/CG10177** (human STK33) [FBgn0039083]	ortholog	**prkaa2** (human PRKAA2) [ZDB-GENE-081120-5]	ortholog	**Hunk** (human HUNK) [MGI:1347352]	ortholog	**PRKAA2** [5563]	ortholog
HAL4	**aak-2** (human PRKAA1, PRKAA2) (AMP-Activated Kinase) [WBGene00020142]	ortholog	**Dmel/AMPKα** (human PRKAA2, PRKAA1) [FBgn0023169]	ortholog	**hunk** (human HUNK) [ZDB-GENE-050309-240]	ortholog	**Prkaa2** (human PRKAA2) [MGI:1336173]	ortholog	**HUNK** [30811]	ortholog
			**Dmel/CG10177** (human STK33) [FBgn0039083]	ortholog	**prkaa2** (human PRKAA2) [ZDB-GENE-081120-5]	ortholog	**Hunk** (human HUNK) [MGI:1347352]	ortholog	**PRKAA2** [5563]	ortholog
HAL5	**aak-2** (human PRKAA1, PRKAA2) (AMP-Activated Kinase) [WBGene00020142]	ortholog	**Dmel/AMPKα** (human PRKAA2, PRKAA1) [FBgn0023169]	ortholog	**hunk** (human HUNK) [ZDB-GENE-050309-240]	ortholog	**Prkaa2** (human PRKAA2) [MGI:1336173]	ortholog	**HUNK** [30811]	ortholog
			**Dmel/CG10177** (human STK33) [FBgn0039083]	ortholog	**prkaa2** (human PRKAA2) [ZDB-GENE-081120-5]	ortholog	**Hunk** (human HUNK) [MGI:1347352]	ortholog	**PRKAA2** [5563]	ortholog
KKQ8	**aak-2** (human PRKAA1, PRKAA2) (AMP-Activated Kinase) [WBGene00020142]	ortholog	**Dmel/AMPKα** (human PRKAA2, PRKAA1) [FBgn0023169]	ortholog	**hunk** (human HUNK) [ZDB-GENE-050309-240]	ortholog	**Prkaa2** (human PRKAA2) [MGI:1336173]	ortholog	**HUNK** [30811]	ortholog
			**Dmel/CG10177** (human STK33) [FBgn0039083]	ortholog	**prkaa2** (human PRKAA2) [ZDB-GENE-081120-5]	ortholog	**Hunk** (human HUNK) [MGI:1347352]	ortholog	**PRKAA2** [5563]	ortholog
PTK1	**W02B12.12** (human TSSK6) [WBGene00012207],	ortholog	**Dmel/CG9222** (human TSSK4) [FBgn0031784]	ortholog	**tssk6** (human TSSK6) [ZDB-GENE-060216-3]	ortholog	**Tssk1** (human TSSK1A) [MGI:1347557]	ortholog	**TSSK2** [23617]	ortholog
	**Y38H8A.4** (human TSSK1B, TSSK2, TSSK6) [WBGene00012638]	ortholog	**Dmel/CG14305** (human TSSK1B) [FBgn0038630]	ortholog			**Tssk2** (human TSSK2) [MGI:1347559]	ortholog	**TSSK4** [283629]	ortholog
	**tag-344** (human TSSK1B, TSSK2, TSSK6) [WBGene00015230]	ortholog					**Tssk4** (human TSSK4) [MGI:1918349]	ortholog	**TSSK3** [81629]	ortholog
	**F23C8.8** [WBGene00017737]	ortholog					**Tssk3** (human TSSK3) [MGI:1929914]	ortholog	**TSSK1B** [83942]	ortholog
	**C27D6.11** (human TSSK1B, TSSK2, TSSK6) [WBGene00044388]	ortholog					**Tssk6** (human TSSK6) [MGI:2148775]	ortholog	**TSSK6** [83983]	ortholog
PTK2	**W02B12.12** (human TSSK6) [WBGene00012207],	ortholog	**Dmel/CG9222** (human TSSK4) [FBgn0031784]	ortholog	**tssk6** (human TSSK6) [ZDB-GENE-060216-3]	ortholog	**Tssk1** (human TSSK1A) [MGI:1347557]	ortholog	**TSSK2** [23617]	ortholog
	**Y38H8A.4** (human TSSK1B, TSSK2, TSSK6) [WBGene00012638]	ortholog	**Dmel/CG14305** (human TSSK1B) [FBgn0038630]	ortholog			**Tssk2** (human TSSK2) [MGI:1347559]	ortholog	**TSSK4** [283629]	ortholog
	**tag-344** (human TSSK1B, TSSK2, TSSK6) [WBGene00015230]	ortholog					**Tssk4** (human TSSK4) [MGI:1918349]	ortholog	**TSSK3** [81629]	ortholog
	**F23C8.8** [WBGene00017737]	ortholog					**Tssk3** (human TSSK3) [MGI:1929914]	ortholog	**TSSK1B** [83942]	ortholog
	**C27D6.11** (human TSSK1B, TSSK2, TSSK6) [WBGene00044388]	ortholog					**Tssk6** (human TSSK6) [MGI:2148775]	ortholog	**TSSK6** [83983]	ortholog

SUPPLEMENTAL TABLE S1. SGD YeastMine was used to search and retrieve *S. cerevisiae* data, populated by SGD and powered by InterMine by using a gene list of kinases clustering with Snf1 to predict orthologs across evolution. Red text = predicted AMPK ortholog, blue text = predicted Snf1-related (SRK) ortholog, and orange text = predicted AMPK-related (ARK) ortholog.

Saccharomyces cerevisiae (budding yeast)	*Caenorhabditis elegans* (worm)		*Drosophila melanogaster* (fly)		*Danio rerio* (zebrafish)		*Mus musculus* (mouse)		*Homo sapiens* (human)	
**VHS1**	**ZK524.4** (human SNRK) [WBGene00013994]	ortholog	**Dmel/CG8485** (human SNRK) [FBgn0033915]	ortholog	**snrkb** (human SNRK) [ZDB-GENE-040426-1724]	ortholog	**Snrk** (human SNRK) [MGI:108104]	ortholog	**SNRK** [54861]	ortholog
					**snrka** (human SNRK) [ZDB-GENE-040718-84]	ortholog				
**SKS1**	**ZK524.4** (human SNRK) [WBGene00013994]	ortholog	**Dmel/CG8485** (human SNRK) [FBgn0033915]	ortholog	**snrkb** (human SNRK) [ZDB-GENE-040426-1724]	ortholog	**Snrk** (human SNRK) [MGI:108104]	ortholog	none	
					**snrka** (human SNRK) [ZDB-GENE-040718-84]	ortholog				
**FRK1**	**W02B12.12** (human TSSK6) [WBGene00012207]	homolog	**Dmel/CG9222** (human TSSK4) [FBgn0031784]	ortholog	**tssk6** (human TSSK6) [ZDB-GENE-060216-3]	ortholog	**Tssk2** (human Tssk2) [MGI:1347559]	homolog	none	
	**Y38H8A.4** (human TSSK1B, TSSK2, TSSK6) [WBGene00012638]	homolog	**Dmel/CG14305** (human TSSK1B) [FBgn0038630]	ortholog			**Tssk4** (human Tssk4) [MGI:1918349]	homolog		
	**tag-344** (human TSSK1B, TSSK2, TSSK6) [WBGene00015230]	homolog, least diverged ortholog, ortholog					**Tssk5** (human Tssk5) [MGI:1920792]	homolog		
	**F23C8.8** [WBGene00017737]	homolog					**Tssk6** (human Tssk6) [MGI:2148775]	homolog		
	**C27D6.11** (human TSSK1B, TSSK2, TSSK6)[WBGene00044388]	homolog								
**KIN4**	**W02B12.12** (human TSSK6) [WBGene00012207]	homolog	**Dmel/CG9222** (human TSSK4) [FBgn0031784]	ortholog	**tssk6** (human TSSK6) [ZDB-GENE-060216-3]	ortholog	**Tssk2** (human Tssk2) [MGI:1347559]	homolog	none	
	**Y38H8A.4** (human TSSK1B, TSSK2, TSSK6) [WBGene00012638]	homolog	**Dmel/CG14305** (human TSSK1B) [FBgn0038630]	ortholog			**Tssk4** (human Tssk4) [MGI:1918349]	homolog		
	**tag-344** (human TSSK1B, TSSK2, TSSK6) [WBGene00015230]	homolog					**Tssk5** (human Tssk5) [MGI:1920792]	homolog		
	**F23C8.8** [WBGene00017737]	homolog					**Tssk6** (human Tssk6) [MGI:2148775]	homolog		
	**C27D6.11** (human TSSK1B, TSSK2, TSSK6)[WBGene00044388]	homolog								
**HSL1**	**kin-29** (human SIK1, SIK2) [WBGene00002210]	homolog	**Dmel/Sik2** (human SIK2) [FBgn0025625]	homolog	**mark2a** (human MARK2) [ZDB-GENE-030131-4145]	homolog	**Sik1** (human SIK1) [MGI:104754]	homolog	[102724428]	
	**par-1** (human MARK1) [WBGene00003916]	homolog	**Dmel/KP78b** (human MARK1) [FBgn0026063]	homolog	**mark3a** (human MARK3) [ZDB-GENE-030131-6232]	homolog	**Snrk** (human SNRK) [MGI:108104]	homolog	**SIK1** [150094]	homolog
	**sad-1** (human BRSK1, BRSK2) [WBGene00004719]	homolog, least diverged ortholog	**Dmel/KP78a** (human MARK1) [FBgn0026064]	homolog	**sik1** (human SIK1) [ZDB-GENE-030131-9446]	homolog	**Mark3** (human MARK3) [MGI:1341865]	homolog	**NIM1K** [167359]	homolog
	**F49C5.4** (human NIM1K) [WBGene00009867]	homolog	**Dmel/CG4629** (human NIM1K) [FBgn0031299]	homolog	**snrkb** (human SNRK) [ZDB-GENE-040426-1724]	homolog	**Mark4** (human MARK4) [MGI:1920955]	homolog	**MARK2** [2011]	homolog
	**ZK524.2** (human SNRK)[WBGene00013994]	homolog	**Dmel/CG8485** (human SNRK) [FBgn0033915]	ortholog	**snrka** (human SNRK) [ZDB-GENE-040718-84]	homolog	**Brsk2** (human BRSK2) [MGI:1923020]	homolog, ortholog	**SIK3** [23387]	homolog
			**Dmel/sff** (human BRSK1) [FBgn0036544]	homolog, least diverged ortholog	**mark4a** (human MARK4) [ZDB-GENE-060531-156]	homolog	**Nim1k** (human NIM1K) [MGI:2442399]	homolog	**MARK1** [4139]	homolog
			**Dmel/par-1** (human MARK3) [FBgn0260934]	homolog	**mark3b** (human MARK3) [ZDB-GENE-060929-80]	homolog	**Sik2** (human SIK2) [MGI:2445031]	homolog	**MARK3** [4140]	homolog
			**Dmel/Sik3** (human SIK3) [FBgn0262103]	homolog	**sik2a** (human SIK2) [ZDB-GENE-070705-451]	homolog	**Sik3** (human SIK3) [MGI:2446296]	homolog	**SNRK** [54861]	homolog
					**sik2b** (human SIK2) [ZDB-GENE-071012-1]	homolog	**Mark1** (human MARK1) [MGI:2664902]	homolog	**MARK4** [57787]	homolog
					**si:ch211-22d5.2** [ZDB-GENE-091204-283]	homolog	**Brsk1** (human BRSK1) [MGI:2685946]	homolog, least diverged ortholog	**BRSK1** [84446]	homolog, least diverged ortholog
					**si:ch211-255p10.4** [ZDB-GENE-121214-354]	homolog, least diverged ortholog	**Mark2** (human MARK2) [MGI:99638]	homolog	**BRSK2** [9024]	homolog, ortholog
					**si:dkey-31m14.7** [ZDB-GENE-121214-92]	homolog				
					**nim1k** (human NIM1K) [ZDB-GENE-130530-744]	homolog				
					**si:dkey-16p21.7** [ZDB-GENE-131122-54]	homolog, ortholog				

SUPPLEMENTAL TABLE S1 Continued

***Saccharomyces cerevisiae* (budding yeast)**	***Caenorhabditis elegans* (worm)**		***Drosophila melanogaster* (fly)**		***Danio rerio* (zebrafish)**		***Mus musculus* (mouse)**		***Homo sapiens* (human)**	
GIN4	**kin-29** (human SIK1, SIK2) [WBGene00002210]	homolog	**Dmel/Sik2** (human SIK2) [FBgn0025625]	homolog	**mark2a** (human MARK2) [ZDB-GENE-030131-4145]	homolog	**Sik1** (human SIK1) [MGI:104754]	homolog	none	
	**par-1** (human MARK1) [WBGene00003916]	homolog	**Dmel/KP78b** (human MARK1) [FBgn0026063]	homolog	**mark3a** (human MARK3) [ZDB-GENE-030131-6232]	homolog	**Snrk** (human SNRK) [MGI:108104]	homolog, ortholog		
	**sad-1** (human BRSK1, BRSK2) [WBGene00004719]	homolog	**Dmel/KP78a** (human MARK1) [FBgn0026064]	homolog	**sik1** (human SIK1) [ZDB-GENE-030131-9446]	homolog	**Mark3** (human MARK3) [MGI:1341865]	homolog		
	**F49C5.4** (human NIM1K) [WBGene00009867]	homolog	**Dmel/CG4629** (human NIM1K) [FBgn0031299]	homolog	**snrkb** (human SNRK) [ZDB-GENE-040426-1724]	homolog, least diverged ortholog	**Mark4** (human MARK4) [MGI:1920955]	homolog		
	**ZK524.2** (human SNRK)[WBGene00013994]	homolog, least diverged ortholog	**Dmel/CG8485** (human SNRK) [FBgn0033915]	homolog, least diverged ortholog	**snrka** (human SNRK) [ZDB-GENE-040718-84]	homolog, ortholog	**Brsk2** (human BRSK2) [MGI:1923020]	homolog		
			**Dmel/sff** (human BRSK1) [FBgn0036544]	homolog	**mark4a** (human MARK4) [ZDB-GENE-060531-156]	homolog	**Nim1k** (human NIM1K) [MGI:2442399]	homolog		
			**Dmel/par-1** (human MARK3) [FBgn0260934]	homolog	**mark3b** (human MARK3) [ZDB-GENE-060929-80]	homolog	**Sik2** (human SIK2) [MGI:2445031]	homolog		
			**Dmel/Sik3** (human SIK3) [FBgn0262103]	homolog	**sik2a** (human SIK2) [ZDB-GENE-070705-451]	homolog	**Sik3** (human SIK3) [MGI:2446296]	homolog		
					**sik2b** (human SIK2) [ZDB-GENE-071012-1]	homolog	**Mark1** (human MARK1) [MGI:2664902]	homolog		
					**si:ch211-22d5.2** [ZDB-GENE-091204-283]	homolog	**Brsk1** (human BRSK1) [MGI:2685946]	homolog		
					**si:ch211-255p10.4** [ZDB-GENE-121214-354]	homolog	**Mark2** (human MARK2) [MGI:99638]	homolog		
					**si:dkey-31m14.7** [ZDB-GENE-121214-92]	homolog				
					**nim1k** (human NIM1k) [ZDB-GENE-130530-744]	homolog				
					**si:dkey-16p21.7** [ZDB-GENE-131122-54]	homolog				
KCC4	**kin-29** (human SIK1, SIK2) [WBGene00002210]	homolog	**Dmel/Sik2** (human SIK2) [FBgn0025625]	homolog	**mark2a** (human MARK2) [ZDB-GENE-030131-4145]	homolog	**Sik1** (human SIK1) [MGI:104754]	homolog	[102724428]	homolog
	**par-1** (human MARK1) [WBGene00003916]	homolog	**Dmel/KP78b** (human MARK1) [FBgn0026063]	homolog	**mark3a** (human MARK3) [ZDB-GENE-030131-6232]	homolog	**Snrk** (human SNRK) [MGI:108104]	homolog, ortholog	**SIK1** [150094]	homolog
	**sad-1** (human BRSK1, BRSK2) [WBGene00004719]	homolog	**Dmel/KP78a** (human MARK1) [FBgn0026064]	homolog	**sik1** (human SIK1) [ZDB-GENE-030131-9446]	homolog	**Mark3** (human MARK3) [MGI:1341865]	homolog	**NIM1K** [167359]	homolog
	**F49C5.4** (human NIM1K) [WBGene00009867]	homolog	**Dmel/CG4629** (human NIM1K) [FBgn0031299]	homolog	**snrkb** (human SNRK) [ZDB-GENE-040426-1724]	homolog, ortholog	**Mark4** (human MARK4) [MGI:1920955]	homolog	**MARK2** [2011]	homolog
	**ZK524.2** (human SNRK)[WBGene00013994]	homolog, ortholog	**Dmel/CG8485** (human SNRK) [FBgn0033915]	homolog, ortholog	**snrka** (human SNRK) [ZDB-GENE-040718-84]	homolog, ortholog	**Brsk2** (human BRSK2) [MGI:1923020]	homolog	**SIK3** [23387]	homolog
			**Dmel/sff** (human BRSK1) [FBgn0036544]	homolog	**mark4a** (human MARK4) [ZDB-GENE-060531-156]	homolog	**Nim1k** (human NIM1K) [MGI:2442399]	homolog	**MARK1** [4139]	homolog
			**Dmel/par-1** (human MARK3) [FBgn0260934]	homolog	**mark3b** (human MARK3) [ZDB-GENE-060929-80]	homolog	**Sik2** (human SIK2) [MGI:2445031]	homolog	**MARK3** [4140]	homolog
			**Dmel/Sik3** (human SIK3) [FBgn0262103]	homolog	**sik2a** (human SIK2) [ZDB-GENE-070705-451]	homolog	**Sik3** (human SIK3) [MGI:2446296]	homolog	**SNRK** [54861]	homolog, ortholog
					**sik2b** (human SIK2) [ZDB-GENE-071012-1]	homolog	**Mark1** (human MARK1) [MGI:2664902]	homolog	**MARK4** [57787]	homolog
					**si:ch211-22d5.2** [ZDB-GENE-091204-283]	homolog	**Brsk1** (human BRSK1) [MGI:2685946]	homolog	**BRSK1** [84446]	homolog
					**si:ch211-255p10.4** [ZDB-GENE-121214-354]	homolog	**Mark2** (human MARK2) [MGI:99638]	homolog	**BRSK2** [9024]	homolog
					**si:dkey-31m14.7** [ZDB-GENE-121214-92]	homolog				
					**nim1k** (human NIM1k) [ZDB-GENE-130530-744]	homolog				
					**si:dkey-16p21.7** [ZDB-GENE-131122-54]	homolog				

SUPPLEMENTAL TABLE S1 Continued

***Saccharomyces cerevisiae* (budding yeast)**	***Caenorhabditis elegans* (worm)**		***Drosophila melanogaster* (fly)**		***Danio rerio* (zebrafish)**		***Mus musculus* (mouse)**		***Homo sapiens* (human)**	
**SNF1**	**aak-2** (AMP-Activated Kinase) [WBGene00020142]	homolog, least diverged ortholog, ortholog	**Dmel/AMPKα** (human PRKAA2, PRKAA1) [FBgn0023169]	homolog, least diverged ortholog, ortholog	**trib3** (human TRIB3) [ZDB-GENE-040426-2609]	homolog	**Melk** (human MELK) [MGI:106924]	homolog	**TRIB1** [10221]	homolog
	**nipi-3** (human TRIB1, TRIB2, TRIB3) [WBGene00010700]	homolog	**Dmel/trbl** (human TRIB2) [FBgn0028978]	homolog	**hunk** (human HUNK) [ZDB-GENE-050309-240]	homolog, ortholog	**Prkaa2** (human PRKAA2) [MGI:1336173]	homolog, least diverged ortholog, ortholog	**TRIB2** [28951]	homolog
	**aak-1** (human PRKAA1, PRKAA2) (AMP-Activated Kinase)[WBGene00019801]	homolog, ortholog	**Dmel/CG10177** (human STK33) [FBgn0039083]	ortholog	**prkaa2** (human PRKAA2) [ZDB-GENE-081120-5]	ortholog, least diverged ortholog	**Trib3** (human TRIB3) [MGI:1345675]	homolog	**HUNK** [30811]	homolog, orhtolog
					**trib2** (human TRIB2) [ZDB-GENE-091207-3]	homolog	**Hunk** (human HUNK) [MGI:1347352]	homolog, ortholog	**PRKAA1** [5562]	homolog, ortholog
					**trib1** (human TRIB1)[ZDB-GENE-091207-4]	homolog	**Stk40** (human STK40) [MGI:1921428]	homolog	**PRKAA2** [5563]	homolog, least diverged ortholog, ortholog
					**melk** (human MELK) [ZDB-GENE-990603-5]	homolog	**Trib2** (human TRIB2) [MGI:2145021]	homolog	**TRIB3** [57761]	homolog
							**Prkaa1** (human PRKAA1) [MGI:2145955]	homolog, ortholog	**STK40** [83931]	homolog
							**Trib1** (human TRIB1) [MGI:2443397]	homolog	**MELK** [9833]	homolog
**PRR1**	**W02B12.12** (human TSSK6) [WBGene00012207]	ortholog	**Dmel/CG9222** (human TSSK4) [FBgn0031784]	ortholog	**tssk6** (human TSSK6) [ZDB-GENE-060216-3]	ortholog	**Tssk4** (human Tssk4) [MGI:1918349]	ortholog	**ULK3** [25989]	ortholog
	**Y38H8A.4** (human TSSK1B, TSSK2, TSSK6) [WBGene00012638]	ortholog	**Dmel/CG14305** (human TSSK1B) [FBgn0038630]	ortholog			**Tssk5** (human Tssk5) [MGI:1920792]	ortholog	**TSSK4** [283629]	ortholog
	**tag-344** (human TSSK1B, TSSK2, TSSK6) [WBGene00015230]	ortholog					**Tssk6** (human Tssk6) [MGI:2148775]	ortholog	**TSSK6** [83983]	ortholog
	**C27D6.11** (human TSSK1B, TSSK2, TSSK6)[WBGene00044388]	ortholog								
**YPL150W**	**aak-2** (AMP-Activated Kinase) [WBGene00020142]	ortholog	**Dmel/AMPKα** (human PRKAA2, PRKAA1) [FBgn0023169]	ortholog	**hunk** (human HUNK) [ZDB-GENE-050309-240]	ortholog	**Prkaa2** (human PRKAA2) [MGI:1336173]	ortholog	none	
			**Dmel/CG10177** (human STK33) [FBgn0039083]	ortholog	**prkaa2** (human PRKAA2) [ZDB-GENE-081120-5]	ortholog	**Hunk** (human HUNK) [MGI:1347352]	ortholog		
							**Smok2a** [MGI:1351487]	homolog, ortholog		
							**1810024B03Rik** [MGI:1925560]	homolog		
							**4921509C19R** [MGI:2685851]	homolog, ortholog		
							[MGI:3036233]	ortholog		
							**Smok2b** [MGI:3037705]	homolog, ortholog		
							**4932414J04Rik** [MGI:3605619]	ortholog		
							**4932415M13Rik** [MGI:3608328]	orhtolog		
							**Smok3b** [MGI:3615348]	homolog, ortholog		
							**Gm10668** [MGI:3642587]	ortholog		
							**Gm10662** [MGI:3642760]	ortholog		
							**Gm7168** [MGI:3643198]	ortholog		
							[MGI:3643324]	ortholog		
							**Gm4922** [MGI:3644318]	ortholog		
							**Gm6176** [MGI:3644439]	ortholog		
							[MGI:3647238]	ortholog		
							**Smok3c** [MGI:3647925]	homolog, ortholog		
							[MGI:3648804]	ortholog		
							**Gm5891** [MGI:3649014]	ortholog		
							**Gm14151** [MGI:3651016]	ortholog		
							**Smok3a** [MGI:3693943]	homolog		
							**Gm4567** [MGI:3809658]	homolog, ortholog		

SUPPLEMENTAL TABLE S1 Continued
