# Supplementary material for: A Snf1-related nutrient-responsive kinase antagonizes endocytosis in yeast
Source: PLoS Genet. 2020 Mar 19;16(3):e1008677. doi: 10.1371/journal.pgen.1008677 (PMC7176151; doi:10.1371/journal.pgen.1008677)
Supplement: S3 Table — (PDF) [file pgen.1008677.s022.pdf]

strain designation	strain background	genotype	source
**WT**	BY4741	*MATa his3Δ0 leu2Δ0 met15Δ0 ura3Δ0*	ResGen™ Collection
**WT**	SEY6210	*MATα leu2-3,112 ura3-52 his3-Δ200 trp1-Δ901 lys2-801 suc2-Δ9*	Robinson et al., 1988
**Nat 1#36**	BY4741	*Δhal4::clonNATR*	This study
**Nat 1#32**	BY4741	*Δhal5::clonNATR*	This study
**Nat 3#86**	SEY6210	*Δhal4::KanMx*	This study
**Nat 3#88**	SEY6210	*Δhal5::KanMx*	This study
**CLY461**	SEY6210	*Δart1::HIS3*	Lin et al., 2008
**JMY1811**	SEY6210	*MUP1-pHluorin::KanMx*	Lee et. al., 2019
**JMY909**	SEY6210	*VPH1-MARS::TRP1*	Lee et. al., 2017
**JTY220**	SEY6210	*HAL5-mNG::KanMx*	This study
**JTY195**	SEY6210	*Δhal4::KanMx VPH1-MARS::TRP1*	This study
**JTY150**	SEY6210	*Δhal5::KanMx VPH1-MARS::TRP1*	This study
**JTY158**	SEY6210	*Δart1::HIS3 VPH1-MARS::TRP1*	This study
**Nat 5#178**	SEY6210	*Δhal4::KanMx MUP1-pHluorin::KanMx*	This study
**Nat 5#180**	SEY6210	*Δhal5::KanMx MUP1-pHluorin::KanMx*	This study
**JTY245**	SEY6210	*Δart1::HIS3 MUP1-pHluorin::KanMx*	Lee et.al. 2019
**Nat 5#170**	SEY6210	*Δhal4::KanMxΔhal5::KanMx*	This study
**JTY229**	SEY6210	*Δhal4::KanMxΔhal5::KanMx VPH1-MARS::TRP*	This study
**JTY154**	SEY6210	*Δhal4::KanMx Δhal5::KanMx MUP1-pHluorin::KanMx*	This study
**JTY247**	SEY6210	*Δart1::HISΔhal4::KanMxΔhal5::KanMx*	This study
**JTY314**	SEY6210	*Δart1::HISΔhal4::KanMx Δhal5::KanMx VPH1-MARS::TRP1*	This study
**JTY306**	SEY6210	*Δart1::HIS3Δhal4::KanMxΔhal5::KanMx MUP1-pHluorin::KanMx*	This study
**JTY285**	SEY6210	*HAL5-mNG::KanMx ART1-mCherry::TRP1*	This study
**JTY287**	SEY6210	*HAL5-mNG::KanMx EDE1-mCherry::TRP1*	This study
**JTY290**	SEY6210	*HAL5-mNG::KanMx SLA2-mCherry::TRP1*	This study
**JTY292**	SEY6210	*HAL5-mNG::KanMx ENT1-mCherry::TRP1*	This study
**JTY294**	SEY6210	*HAL5-mNG::KanMx PIL1-mCherry::TRP1*	This study
**JTY295**	SEY6210	*HAL5-mNG::KanMx ABP1-mCherry::TRP1*	This study
**JTY308**	SEY6210	*HAL5-mNG::KanMx MUP1-MARS::TRP1*	This study
**JTY283**	SEY6210	*Δhal4::KanMxΔhal5::KanMxΔarg4::KanMx ART1-HTF::TRP*	This study
**JTY255**	SEY6210	*Δarg4::KanMx ART1-HTF::TRP*	This study
**JTY254**	SEY6210	*Δhal4::KanMxΔhal5::KanMxΔarg4::KanMx*	This study
**JTY259**	SEY6210	*Δhal4::KanMx Δarg4::KanMx*	This study
**JTY260**	SEY6210	*Δhal5::KanMxΔarg4::KanMx*	This study

SUPPLEMENTAL TABLE S3. Strains generated and/or used in this study including: strain designation, background, genotype, and source.
