# Supplementary material for: A Snf1-related nutrient-responsive kinase antagonizes endocytosis in yeast
Source: PLoS Genet. 2020 Mar 19;16(3):e1008677. doi: 10.1371/journal.pgen.1008677 (PMC7176151; doi:10.1371/journal.pgen.1008677)
Supplement: S4 Table — (PDF) [file pgen.1008677.s023.pdf]

plasmid designation	plasmid backbone	genotype	source
**pCHL642**	pRS416	MUP1-GFP	Lin et. al., 2008
**pSR21**	pRS416	FUR4-GFP	Lin et. al., 2008
**pCHL571**	pRS416	CAN1-GFP	Lin et. al., 2008
**Pil1-GFP**	pRS416	PIL1-GFP	This study
**Snc1-GFP**	pRS416	SNC1-GFP	Lewis et al., 2000
**Cps-GFP**	pRS416	CPS-GFP	This study
**pJAM1292**	pRS415	native Hal5	This study
**pJAM1368**	pRS415	HAL5-adh1terminator-HTF	This study
**pJMT4**	pRS415	*hal5K546R*-adh1 terminator-HTF	This study
**pJMT5**	pRS415	*hal5D688A*-adh1 terminator-HTF	This study
**pJMT6**	pRS415	*hal5M620G*-adh1 terminator-HTF	This study
**pJMT10**	pRS415	*hal5Δ1-493*	This study
**pJMT11**	pRS415	*hal5Δ1-493*-adh1 terminator-HTF	This study
**pJAM1583**	pRS415	HAL5-mNG	This study
**pJAM1585**	pRS415	*hal5Δ494-855*-mNG	This study
**pJMT13**	pRS415	*hal5Δ1-493*-mNG	This study
**pJMT14**	pRS415	*hal5M620G*-mNG	This study
**pJAM1621**	pRS415	*hal5Δ1-339*	This study
**pJAM1622**	pRS415	*hal5Δ1-248*	This study
**pJAM1623**	pRS415	*hal5Δ1-99*	This study
**pJMT18**	pRS415	*hal5Δ1-339-mNG*	This study
**pJMT19**	pRS415	*hal5Δ1-339*-adh1 terminator-HTF	This study
**pJMT21**	pRS415	*hal5Δ1-248-mNG*	This study
**pJMT23**	pRS415	*hal5Δ1-99-mNG*	This study
**pJAM1547**	pRS415	ART1-mNG	This study

SUPPLEMENTAL TABLE S4. Plasmids generated and/or used in this study including: plasmid designation, backbone, genotype, and source.
