# Supplementary material for: A Snf1-related nutrient-responsive kinase antagonizes endocytosis in yeast
Source: PLoS Genet. 2020 Mar 19;16(3):e1008677. doi: 10.1371/journal.pgen.1008677 (PMC7176151; doi:10.1371/journal.pgen.1008677)
Supplement: S2 Table — (PDF) [file pgen.1008677.s021.pdf]

organism	AMPK			ARKs	SRKs
Human	α	β	γ	BRSKs<br>NUAKs<br>QIK<br>QSK<br>SIK<br>MARKs<br>MELK	NIM<br>SNRK<br>TSSKs<br>SSTK<br>HUNK
	PRKAA1<br>PRKAA2	PRKAB1<br>PRKAB2	PRKAG1<br>PRKAG2<br>PRKAG3		
	(12 distinct isoforms)				
Yeast	α	β	γ	Frk1<br>Hsl1<br>Kcc4<br>YPL150W<br>Kin1<br>Kin2<br>Kin4<br>Gin4	Prr1<br>Prr2<br>Npr1<br>Hrk1<br>Rtk1<br>Hal4<br>Hal5<br>Kkq8<br>Ptk1<br>Ptk2<br>Vhs1<br>Sks1<br>Nnk1
	Snf1	Gal83<br>Sip1<br>Sip2	Snf4		
	(3 distinct isoforms)				

SUPPLEMENTAL TABLE S2. Sub-classification of the human and yeast families of kinases related to AMPK and Snf1, respectively.
